# Decoding herbal materials of TCM preparations with the multi-barcode sequencing approach

**DOI:** 10.1038/s41598-022-09979-z

**Published:** 2022-04-09

**Authors:** Qi Yao, Xue Zhu, Maozhen Han, Chaoyun Chen, Wei Li, Hong Bai, Kang Ning

**Affiliations:** 1 Key Laboratory of Molecular Biophysics of the Ministry of Education, Hubei Key Laboratory of Bioinformatics and Molecular-Imaging, Center of AI Biology, Department of Bioinformatics and Systems Biology, College of Life Science and Technology, Huazhong University of Science and Technology, Wuhan, 430074 Hubei China; 2 Faculty of Pharmaceutical Sciences, Toho University, Tokyo, 1438540 Japan

**Keywords:** Computational biology and bioinformatics, Molecular biology

## Abstract

With the rapid development of high-throughput sequencing technology, approaches for assessing biological ingredients in Traditional Chinese Medicine (TCM) preparations have also advanced. Using a multi-barcode sequencing approach, all biological ingredients could be identified from TCM preparations in theory, as long as their DNA is present. The biological ingredients of several classical TCM preparations were analyzed successfully based on this approach in previous studies. However, the universality, sensitivity and reliability of this approach on a diverse set of TCM preparations remain unclear. In this study, we selected four representative TCM preparations, namely Bazhen Yimu Wan, Da Huoluo Wan, Niuhuang Jiangya Wan, and You Gui Wan, for concrete assessment of the multi-barcode sequencing approach. Based on ITS2 and *trnL* biomarkers, we have successfully detected the prescribed herbal materials (PHMs) in these representative TCM preparations (minimum sensitivity: 77.8%, maximum sensitivity: 100%). The results based on ITS2 have also shown higher reliability than *trnL* at species level, while their combination could provide higher sensitivity and reliability. The multi-barcode sequencing approach has shown good universality, sensitivity and reliability in decoding these four representative TCM preparations. In the omics big-data era, this work has undoubtedly made one step forward for applying multi-barcode sequencing approach in PHMs analysis of TCM preparation, towards better digitization and modernization of drug quality control.

## Introduction

Traditional Chinese Medicine (TCM) preparation has been used in clinics in China for at least 3000 years^1,2^. It has been utilized to prevent and cure various diseases in China, and has become more popular worldwide during the last decades. TCM preparation is composed of numerous plants, animal-derived and/or mineral materials. According to the guidance of Chinese medicine theory and Chinese Pharmacopoeia (ChP)^3^, different medicinal materials were crushed into powder, or boiled, then mixed and molded into pills together with honey or water to get a TCM preparation (also called patented drug). Although TCM preparations have been extensively used in recent years, many problems remain to be resolved, such as quality control (QC), in which particular attention should be focused on its materials and production process to ensure its safety and efficacy. The TCM quality assessment mainly includes the qualitative and quantitative analysis of chemical ingredients and biological ingredients^4^. Current methods for TCM preparations QC have been mainly assessed based on chemical profiling^4^ (e.g. thin-layer chromatography (TLC)^5^, high-performance liquid chromatography ultraviolet (HPLC–UV)^6^, high-performance liquid chromatography-mass spectrometry (HPLC–MS)^7^). In comparison to reference herbal materials or targeted compounds, TLC and HPLC methods can retrieve species information but are not precise enough, especially for identifying the hybrid species of genetics, which might occur the incorrect identification, and introduce biological pollution and adulteration during the herbal materials collection and manufacturing process. However, the utilization of DNA, a fragment that stably exists in all tissues^8^, could accurately identify herbal materials at species level, providing a higher level of sensitivity and reliability, and thus complement the drawback of chemical analysis^9,10^.

The concept of biological ingredient analysis based on DNA barcodes was proposed by Hebert^11^. Chen et al. have first applied several candidate DNA barcodes to identify medicinal plants and their closely related species^12^. Coghlan et al., for the first time, have used DNA barcodes to determine whether TCM preparations contain derivatives of endangered, trade-restricted species of plants and animals^2^. In 2014, Cheng et al. have first reported the biological ingredients analysis for Liuwei Dihuang Wan (LDW) using the metagenomic-based method based on ITS2 and *trnL* biomarkers^13^. After that, the reports on the herbs of TCM preparations based on DNA biomarkers have been sprung up, such as Yimu Wan (YMW)^14^, Longdan Xiegan Wan (LXW)^15^ and Jiuwei Qianghuo Wan (JQW)^16^. Interestingly, recent studies have reported several TCM preparations that might be effective in the prevention and treatment for COVID-19^17,18^, such as Lianhua Qingwen capsule^19^, Jinhua Qinggan granules^19^, Yiqi Qingjie herbal compound^20^, etc. Helped by the DNA barcode technology, it was reported that Lianhua Qingwen capsule might be effective in preventing or treating COVID-19, which might be due to its biological ingredients, such as Glycyrrhizae Radix Et Rhizoma and Rhei Radix Et Rhizome^3^. The same principle applies to Jinhua Qinggan granules and Yiqi Qingjie herbal compounds. These findings again emphasized the importance of biological ingredient analysis of TCM preparations using DNA barcode approach.

A TCM preparation can be regarded as a “synthesized mixture of species”, which resembles the analytical target of the metagenomic approach. Based on suitable DNA biomarkers, the genetic information of all DNA-contained ingredients could be obtained most effectively and cost-effectively via high-throughput sequencing. Due to the conservation of ITS2^21^ and its high inter-specific and intra-specific divergence power^22–24^, as well as the convenience of amplification DNA from heavily degraded samples based on a short fragment *trnL*^25–27^, these two fragments are usually chosen as biomarkers for herbal species identification. Such an approach based on multiple barcodes for herbal ingredient analysis is referred to as the “multi-barcoding approach”, or “multi-barcode sequencing approach”.

Despite scientific advances of recent studies, the solidity (i.e., universality, sensitivity and reliability) of the multi-barcode sequencing approach on identifying various biological ingredients of TCM preparations remains unclear. Therefore, we selected four representative TCM preparations, including three pervasively used TCM preparations Niuhuang Jiangya Wan (NJW), Bazhen Yimu Wan (BYW), and Yougui Wan (YGW) with simple compositions, as well as Da Huoluo Wan (DHW) with much more complicated components, as targets for herbal materials assessment by using ITS2 and *trnL* biomarkers. Based on assessing the prescribed herbal species (PHS) of the prescribed herbal materials (PHMs), the universality, sensitivity and reliability of the multi-barcode sequencing approach have been evaluated, which confirmed its power in PHMs assessment for TCM preparations.

## Results

### Profiling the PHMs for all TCM preparations in Chinese pharmacopoeia

**Figure 1:**
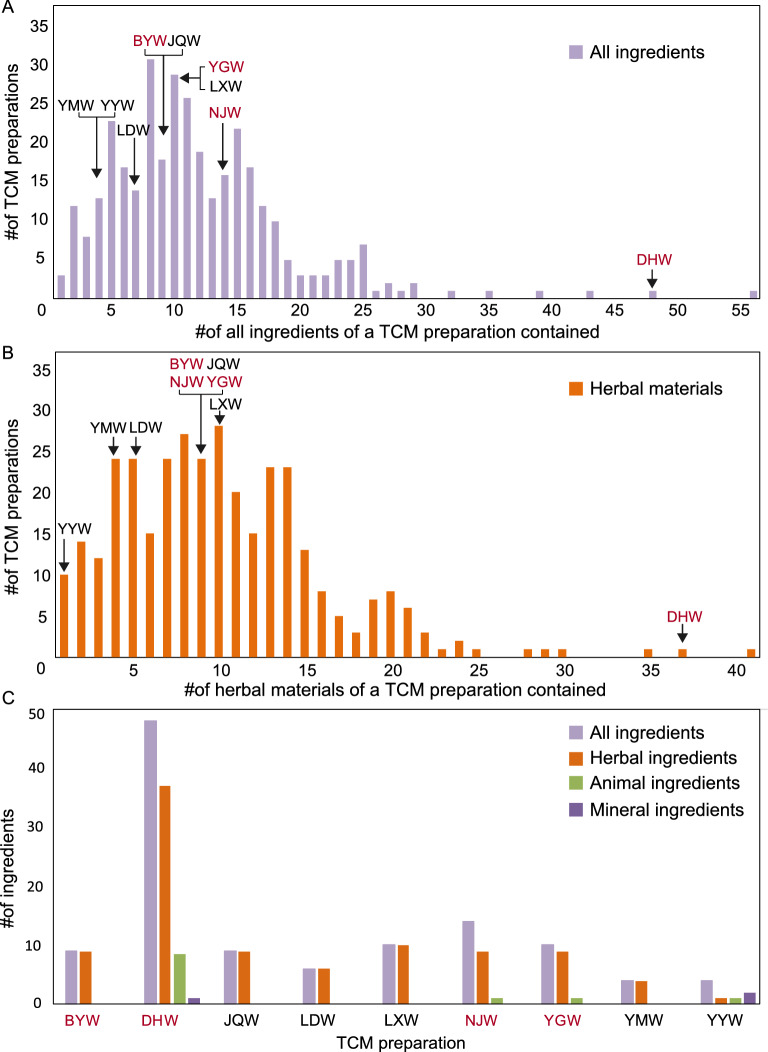
The distribution of all ingredients and the herbal materials only of all TCM preparations listed in the Chinese pharmacopoeia. (**A**) All ingredients of TCM preparation, including herbal, animal and mineral materials. (**B**) The herbal materials of a TCM preparation contained. The x-axis represents the number of all/herbal ingredients of a TCM preparation contained, y-axis means the corresponding number of the TCM preparations. The abbreviations are shown, from left to right, Yatong Yili Wan (YYW), Yimu Wan (YMW), Liuwei Dihuang Wan (LDW), Bazhen Yimu Wan (BYW), Yougui Wan (YGW), Niuhuang Jiangya Wan (NJW), Jiuwei Qianghuo Wan (JQW), Longdan Xiegan Wan (LXW) and Da Huoluo Wan (DHW), respectively. (**C**) The distribution of all ingredients and the detailed ingredient of nine TCM preparations. The abbreviations are shown, from left to right, Bazhen Yimu Wan (BYW), Da Huoluo Wan (DHW), Jiuwei Qianghuo Wan (JQW), Liuwei Dihuang Wan (LDW), Longdan Xiegan Wan (LXW), Niuhuang Jiangya Wan (NJW), Yougui Wan (YGW), Yimu Wan (YMW) and Yatong Yili Wan (YYW), respectively. The words marked in black are those already reported in previous studies, while the words marked in red represent the research preparations used in this work.

Though several widely-used TCM preparations, including LDW^13^, YMW^14^, LXW^15^, and JQW^16^, have their herbal materials assessed recently, it is important to choose representative preparations for a deeper understanding of the performance of multi-barcode sequencing approach. Thus, we examined all ingredients (including herbs, animals, and minerals) and herbal materials only for the TCM preparations recorded in ChP (2015 version) (Fig. 1A,B)^3^. Most TCM preparations have less than 25 ingredients and 20 herbal materials, respectively. Therefore, we selected TCM preparations with pervasive application from simple compositions to complex compositions, for assessing the multi-barcode sequencing approach. Among them, BYW, NJW, and YGW have simple compositions, and DHW has much more complex ingredients (Fig. 1C and Supplementary Table S1).

### Overview of the herbal materials from TCM preparations

For these four representative TCM preparations, after preliminary quality control (QC) (see more details in “Methods” section), we obtained 25,271,042 ITS2 and 27,599,145 *trnL* sequencing reads. An average of 48,493 ITS2 sequencing reads for BYW, 87,911 for DHW, 161,025 for NJW, and 58,501 for YGW were detected in each sample. An average of 57,954 *trnL* reads for BYW, 139,521 for DHW, 129,560 for NJW, and 61,685 for YGW were detected (Table 1). The length (maximum, average, and minimum length) of each sequence obtained from these TCM preparation samples was shown in Supplementary Table S2. Then rarefaction analysis was performed for each sample to detect the sequencing depth. All rarefaction curves reached saturated at around 10,000 sequences per sample (Supplementary Fig. S1), suggesting that the sequencing depth was enough to capture all species information in all samples for the four TCM preparations. Considering the *trnL* database was smaller compared with ITS2, we filtered the species detected by ITS2 barcode with the relative abundance below 0.002, and the species detected by *trnL* barcode with the relative abundance below 0.001, respectively. After that, an average sequence of 47,533 for BYW samples, 86,642 for DHW samples, 160,712 for NJW samples and 58,008 for YGW samples were obtained based on ITS2, and 56,367 (BYW), 130,330 (DHW), 129,012 (NJW) and 59,709 (YGW) were obtained based on *trnL*, respectively (Table 1).

**Table 1:** The average number of reads of each sample after preliminary quality control and threshold filtration for the four TCM preparations.

	Biomarker	BYW	DHW	NJW	YGW
Preliminary QC	ITS2 (150–510 bp)	48,493	87,911	161,025	58,501
	*trnL* (≥ 75 bp)	57,954	139,521	129,560	61,685
Threshold selection	ITS2 (≥ 0.002)	47,553	86,642	160,712	58,008
	*trnL* (≥ 0.001)	56,367	130,330	129,012	59,709

Note that, QC means quality control, we removed the reads that were below 150 bp or over 510 bp for ITS2, and the reads less than 75 bp for *trnL*, or the sequences that had an average quality score < 20 in each 5 bp-window rolling along with the whole read. Then we filtered out the species whose relative abundance was less than 0.002 for ITS2 and 0.001 for *trnL.*

In general, several herbal materials have more than one PHS. For example, licorice has three species: *Glycyrrhiza uralensis*, *Glycyrrhiza inflate*, and *Glycyrrhiza glabra*. Consequently, anyone original species of PHMs should be regarded as PHS. For instance, BYW contains eight PHMs, NJW and YGW have nine PHMs, and DHW contains 36 PHMs, while they include 11, 15, 10, and 57 PHS, respectively (Table 2 and Supplementary Table S3).

**Table 2:** The abbreviation of prescribed herbal materials and their corresponding prescribed herbal species of Yougui Wan (YGW) recorded in the Chinese pharmacopoeia.

TCM preparation	Prescribed herbal material (PHM)	Prescribed herbal species (PHS)
Yougui Wan (YGW)	Aconitum carmichaelii Debx	*Aconitum carmichaeli*
	Angelica sinensis (Oliv.) Diels	*Angelica sinensis*
	Cinnamomum cassia Presl	*Cinnamomum cassia*
	Cornus officinalis Sieb. et Zucc	*Cornus officinalis*
	Cuscuta australis R. Br	*Cuscuta australis*
		*Cuscuta chinensis*
	Dioscorea opposita Thunb	*Dioscorea opposita*
	Eucommia ulmoides Oliv	*Eucommia ulmoides*
	Lycium barbarum L.	*Lycium barbarum*
	Rehmanniae radix praeparata	*Rehmannia glutinosa*

Un-prescribed herbal materials mainly include the substituted herbal species (SHS) and contaminated herbal species (CHS).

**Figure 2:**
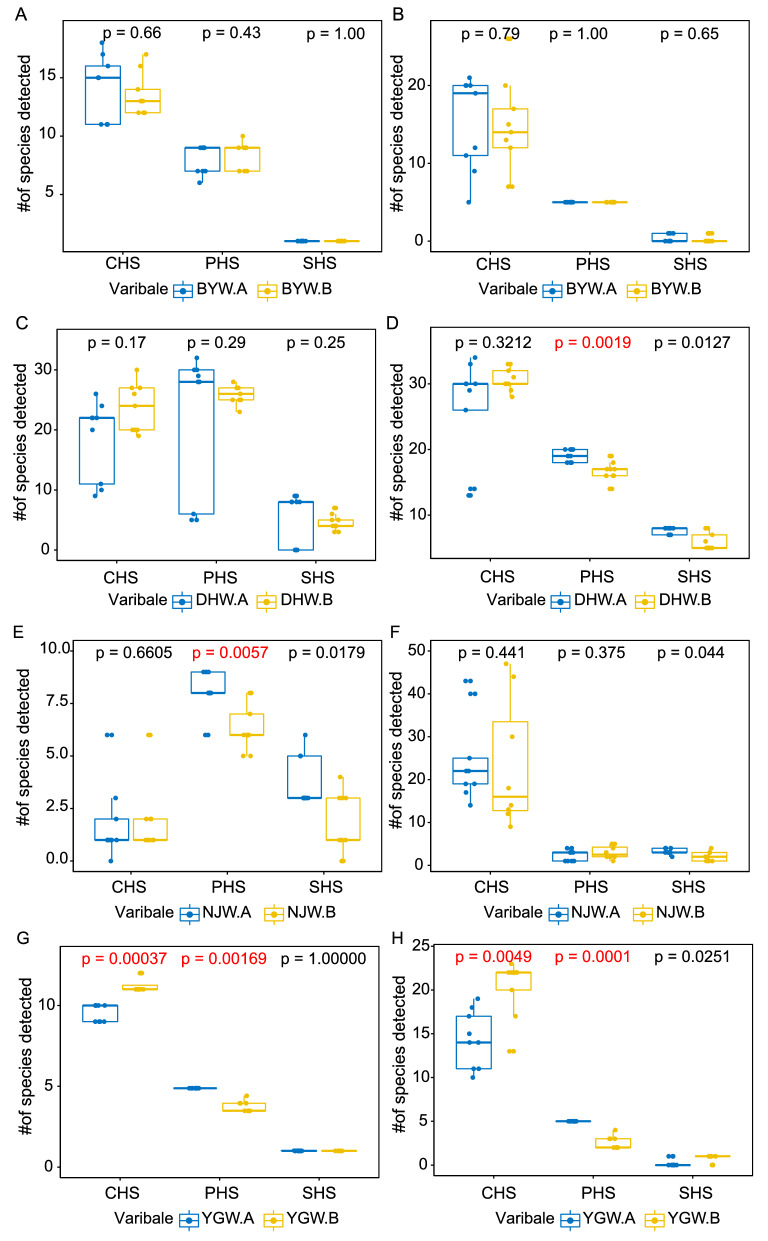
The distribution of detected species in four representative TCM preparations bought from two manufacturers based on ITS2 and *trnL*. (**A**) BYW samples based on ITS2; (**B**) BYW samples based on *trnL*; (**C**) DHW samples based on ITS2; (**D**) DHW samples based on *trnL*; (**E**) NJW samples based on ITS2; (**F**) NJW samples based on *trnL*. (**G**) YGW samples based on ITS2; (**H**) YGW samples based on *trnL*. *PHS* prescribed herbal species, *SHS* substituted herbal species, *CHS* contaminated herbal species.

The results of the ITS2 audit on 18 BYW samples showed that on average of 8.2 PHS, 1.0 substituted herbal species (SHS), and 13.8 contaminated herbal species (CHS) were detected, while 5.0 PHS, 0.3 SHS, and 14.9 CHS were found in each *trnL* sample (Fig. 2A,B). For DHW, each sample has an average of 23.7 PHS, 5.1 SHS, and 21.1 CHS based on ITS2, while an average of 17.9 PHS, 6.8 SHS, and 27.7 CHS based on *trnL* (Fig. 2C,D). For NJW samples, an average of 7.2 PHS, 2.8 SHS, and 1.8 CHS was detected in each sample based on ITS2, which was more than *trnL* (3.0 PHS, 3.0 SHS, and 24.0 CHS; Fig. 2E,F). The mean values of PHS, SHS, and CHS detected in each YGW sample were 4.8, 0.9, and 10.4 based on ITS2, and 3.7, 0.5, and 17.3 were based on *trnL*, respectively (Fig. 2G,H). These differences may partially be due to the completeness of the ITS2 and *trnL* database, as well as their intrinsic resolution properties.

In summary, the multi-barcode sequencing approach could detect the herbal materials, including prescribed, substituted, and contaminated materials, for representative TCM preparations (including BYW, DHW, NJW, and YGW). The result has demonstrated that the multi-barcode sequencing approach has good universality in detecting PHMs from TCM preparation samples.

### Sensitivity analysis of PHMs from TCM preparations

Further investigation was performed to detect the composition of TCM preparations, we chose one TCM preparation (NJW) with a relatively simple composition and pervasively application, and another TCM preparation (DHW) with more complex ingredients, as targets to decode their PHMs through identifying their PHS of each TCM preparations based on ITS2 and *trnL* datasets, respectively.

#### Analysis of herbal materials in the TCM preparations based on ITS2

**Figure 3:**
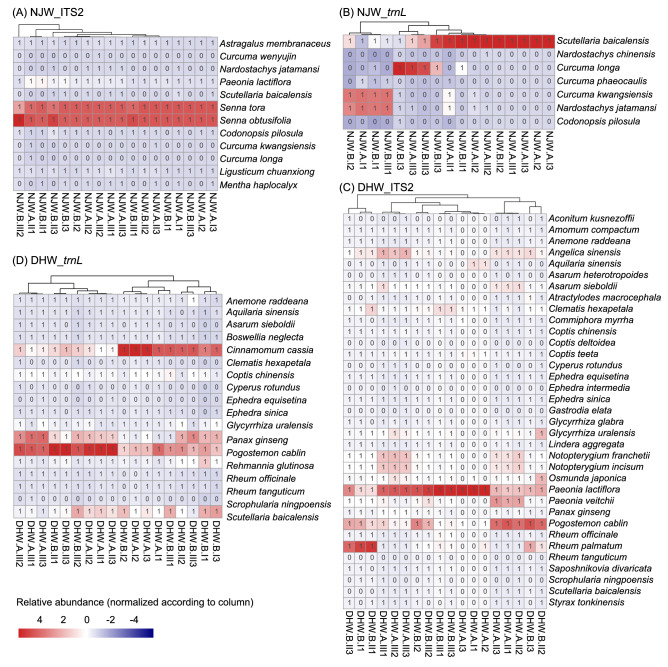
The distribution of prescribed herbal species (PHS) detected in each sample from NJW and DHW preparations. (**A**) The detected PHS in NJW samples based on ITS2; (**B**) The detected PHS in NJW samples based on *trnL*; (**C**) The detected PHS in DHW samples based on ITS2; (**D**) The detected PHS in DHW samples based on *trnL.* Note that each column represents a sample, and each row represents a PHS. In the heatmap that was drawn in R (version 3.5.2) package “pheatmap” (https://cran.rstudio.com/web/packages/pheatmap/index.html), the color represents the relative abundance of PHS (normalized according to column) detected in this sample. The number “1” represents the PHS that was detected in this sample, while “0” represents the PHS that was not detected.

The result of the ITS2 auditing on NJW samples, revealed that it could successfully detect all PHMs (9 herbal materials), including the processed herbal materials (such as Scutellaria extract), covering 12 detected PHS (Table 3, Fig. 3A and Supplementary Fig. S2C). *Senna obtusifolia* (the average relative abundance was 48.4%) and *Senna tora* (45.4%) were the dominant species in all samples, followed by *Paeonia lactiflora* (3.4%) and *Ligusticum chuanxiong* (1.0%). The results suggested that the modified CTAB method was suitable for extracting their DNA, and the primers were more suitable to amply their sequences. Besides the PHS, seven SHS were also found, belonging to *Codonopsis*, *Ligusticum*, *Mentha*, *Paeonia* and *Senna* (their average relative abundance was 0.035%) and six possible contaminated genera, namely *Ipomoea*, *Amaranthus*, *Anemone*, *Cuscuta*, *Pogostemon* and *Zanthoxylum*, which might be introduced during the biological experiment or manufacturing process.

**Table 3:** Prescribed herbal species for NJW preparation and their presence in each sample by the multi-barcode sequencing approach based on ITS2 biomarker.

Prescribed herbal species (PHS)	NJW.A									NJW.B								
	I1	I2	I3	II1	II2	II3	III1	III2	III3	I1	I2	I3	II1	II2	II3	III1	III2	III3
*Astragalus membranaceus*	√	√	√	√	√	√	√	√	√	√	√	√	√	√	√	√	√	√
*Codonopsis pilosula*	√	√	√	√	√	√	√	√	√		√	√	√	√	√	√	√	
*Curcuma kwangsiensis*				√		√												
*Curcuma longa*				√														
*Curcuma wenyujin*								√										
*Ligusticum chuanxiong*	√	√	√	√	√	√	√	√	√	√	√	√	√	√	√	√	√	√
*Mentha haplocalyx*	√		√		√	√	√		√			√	√					
*Nardostachys jatamansi*	√				√		√	√	√						√			
*Paeonia lactiflora*	√	√	√	√	√	√	√	√	√	√	√	√	√	√	√	√	√	√
*Scutellaria baicalensis*	√		√		√		√		√			√			√			
*Senna obtusifolia*	√	√	√	√	√	√	√	√	√	√	√	√	√	√	√	√	√	√
*Senna tora*	√	√	√	√	√	√	√	√	√	√	√	√	√	√	√	√	√	√

Note that “NJW.A” and “NJW.B” means the “Niuhuang Jiangya Wan” bought from manufacturer A and B, respectively. “I1” represents the sample as one of three biological replicates of the first batch sample, and the “√” means that the prescribed herbal species is detected in this sample.

**Figure 4:**
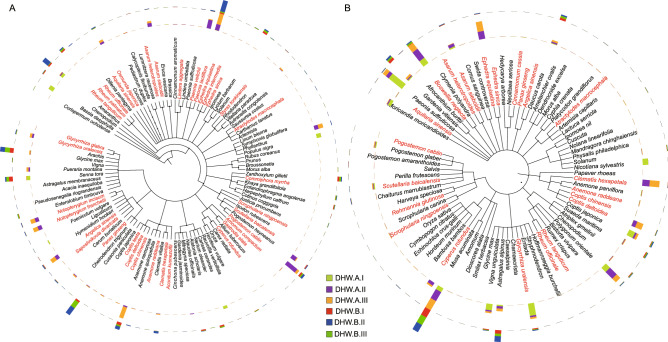
Phylogenetic analysis of the representative species that had at least 0.1% relative abundance in DHW samples. (**A**) Based on ITS2; (**B**) Based on *trnL*. The phylogenetic trees of species are visualized in iTOL (https://itol.embl.de/). The branch in the tree depicts the taxonomic classification of species. The word marked in red means the prescribed herbal species, and the colorful bar means the average relative abundance of species across the three batches from the two manufacturers (**A**,**B**).

For DHW preparation, we detected 35 PHS covering 25 PHMs, including the processed herbal materials such as the stir-fried Baishu. The sensitivity of PHMs was 69.4% based on ITS2 (Table 4, Figs. 3C, 4A). Among the detected PHS from 18 samples, 15 PHS were found with an average relative abundance over 0.1%, where seven PHS were identified with an average relative abundance over 1%, including *Angelica sinensis* (2.0%), *Asarum sieboldii* (1.2%), *Notopterygium franchetii* (1.9%), *Notopterygium incisum* (1.8%), *Paeonia lactiflora* (5.3%), *Paeonia veitchii* (2.0%) and *Pogostemon cablin* (3.7%). Three PHS (*Clematis hexapetala*, *Coptis teeta*, *Paeonia lactiflora*) were found in all samples. Among them, *Paeonia lactiflora* was highly enriched in DHW.A samples. The average relative abundance of *Glycyrrhiza uralensis* (1.56%) and *Osmunda japonica* (1.64%) detected in samples from DHW.A was 1.6 times more than DHW.B samples (*Glycyrrhiza uralensis* (0.94%) and *Osmunda japonica* (0.98%)). While *Coptis deltoidei* (one read detected in DHW.A.III3), *Ephedra intermedia* (three reads detected in DHW.A.III2), *Gastrodia elata* (one read in DHW.A.III3) and *Rheum tanguticum* (three reads detected in DHW.B.III1) were only detected in one sample. Noticeably, the SHS, belonging to *Anemone nemorosa* (0.31%) that have the same genus with PHS, were found with high relative abundance in most samples, especially in DHW.A.II and DHW.A.III, which might be introduced during manufacturer processing or biological experiment.

**Table 4:** Prescribed herbal species for DHW preparation and their presence in each sample by the multi-barcode sequencing approach based on ITS2 biomarker.

Prescribed herbal species (PHS)	DHW.A									DHW.B								
	I1	I2	I3	II1	II2	II3	III1	III2	III3	I1	I2	I3	II1	II2	II3	III1	III2	III3
*Aconitum kusnezoffii*				√	√				√					√	√			
*Amomum compactum*				√	√	√	√	√	√	√	√	√	√	√	√	√	√	√
*Anemone raddeana*				√	√	√	√	√	√	√	√	√	√	√	√	√	√	√
*Angelica sinensis*				√	√	√	√	√	√	√	√	√	√	√	√	√	√	√
*Aquilaria sinensis*	√	√		√	√		√		√	√	√						√	
*Asarum heterotropoides*					√	√	√	√	√								√	
*Asarum sieboldii*				√	√	√	√	√	√	√	√	√	√	√	√	√	√	√
*Atractylodes macrocephala*				√	√	√	√	√	√		√	√	√	√		√		√
*Clematis hexapetala*	√	√	√	√	√	√	√	√	√	√	√	√	√	√	√	√	√	√
*Commiphora myrrha*			√	√	√	√	√	√	√	√	√	√		√	√	√	√	√
*Coptis chinensis*				√	√	√	√	√	√	√	√	√	√	√	√	√	√	√
*Coptis deltoidea*									√									
*Coptis teeta*	√	√	√	√	√	√	√	√	√	√	√	√	√	√	√	√	√	√
*Cyperus rotundus*						√	√	√	√					√				
*Ephedra equisetina*				√	√	√	√	√	√	√	√	√	√	√	√	√	√	√
*Ephedra intermedia*								√										
*Ephedra sinica*				√	√	√	√	√	√	√	√	√	√	√	√	√	√	√
*Gastrodia elata*									√									
*Glycyrrhiza glabra*				√	√	√	√	√	√	√	√	√		√	√	√	√	√
*Glycyrrhiza uralensis*				√	√	√	√	√	√	√	√	√	√	√	√	√	√	√
*Lindera aggregata*				√	√	√	√	√	√	√	√	√	√	√	√	√	√	√
*Notopterygium franchetii*				√	√	√	√	√	√	√	√	√	√	√	√	√	√	√
*Notopterygium incisum*				√	√	√	√	√	√	√	√	√	√	√	√	√	√	√
*Osmunda japonica*	√			√	√	√	√	√	√	√	√	√	√	√	√	√	√	√
*Paeonia lactiflora*	√	√	√	√	√	√	√	√	√	√	√	√	√	√	√	√	√	√
*Paeonia veitchii*				√	√	√	√	√	√	√	√	√	√	√	√	√	√	√
*Panax ginseng*				√	√	√	√	√	√	√	√	√	√	√	√	√	√	√
*Pogostemon cablin*		√	√	√	√	√	√	√	√	√	√	√	√	√	√	√	√	√
*Rheum officinale*				√	√	√	√	√	√	√	√	√	√	√	√	√	√	√
*Rheum palmatum*		√		√	√	√	√	√	√	√	√	√	√	√	√	√	√	√
*Rheum tanguticum*																√		
*Saposhnikovia divaricata*				√	√	√	√	√	√	√	√	√		√	√	√	√	√
*Scrophularia ningpoensis*					√		√			√			√			√	√	
*Scutellaria baicalensis*				√	√	√	√	√	√		√	√		√		√	√	√
*Styrax tonkinensis*				√	√	√	√	√	√		√		√	√	√			√

#### Analysis of herbal materials in the TCM preparations based on *trnL*

For NJW, seven PHS belonged to four genera were detected with low abundance, including *Codonopsis pilosula*, *Curcuma kwangsiensis*, *Curcuma longa*, *Curcuma phaeocaulis*, *Nardostachys chinensis*, *Nardostachys jatamansi, Scutellaria baicalensis* (Table 5, Fig. 3B and Supplementary Fig. S2D). Among them, *Nardostachys chinensis* was captured in all samples, while *Codonopsis pilosula* and *Nardostachys jatamansi* were only identified in one sample with one read, which suggested that the DNA of these low relative abundance species was hard to be extracted or the *trnL* c/h primers were not suitable for the determination them. The substituted *Astragalus* (3.9%) and *Mentha* (8.1%) were identified with high relative abundance. As for possible CHS, they were dispersedly distributed in 52 genera.

**Table 5:** Prescribed herbal species for NJW preparation and their presence in each sample by the multi-barcode sequencing approach based on *trnL* biomarker.

Prescribed herbal species (PHS)	NJW.A									NJW.B							
	I1	I2	I3	II1	II2	II3	III1	III2	III3	I1	I2	I3	II1	II2	II3	III1	III3
*Nardostachys chinensis*	√	√	√	√	√	√	√	√	√	√	√	√	√	√	√	√	√
*Nardostachys jatamansi*								√									
*Scutellaria baicalensis*									√	√		√	√		√		√
*Curcuma kwangsiensis*	√			√						√						√	
*Curcuma longa*	√			√	√					√	√	√				√	
*Curcuma phaeocaulis*	√			√	√			√	√	√	√	√				√	
*Codonopsis pilosula*												√					

For DHW samples based on *trnL*, because of its complex biological ingredients, the sensitivity of PHMs (18 PHMs, 22 PHS) was only 50% (Table 6, Figs. 3D and 4B). Among 22 detected PHS, 12 of them (Table 6) were detected in all samples with an average relative abundance greater than 0.1%, except *Coptis chinensis* (0.05%), in which six of them exceeded 1%, 10 of 22 PHS were below 0.05%. Moreover, the relative abundance of 22 PHS that were detected from DHW.A, was higher than DHW.B. *Boswellia neglecta* (6.4%) was the dominant species, followed by *Glycyrrhiza uralensis* (4.2%), and then *Coptis deltoidei* (2.6%). Nevertheless, *Ephedra equisetina* (12 reads in DHW.A.II3 and 8 reads in DHW.A.III3) and *Scrophularia ningpoensis* (one read in both DHW.A.I2 and DHW.A.III1) were only found in two samples. The reason for this low abundant PHS might be due to the processing in the manufacturers. For example, the materials stir-fried Baishu, vinegar-process Xiangfu and other materials might be boiled or fried before adding into a TCM preparation, resulting in their DNA damage.

**Table 6:** Prescribed herbal species for DHW preparation and their presence in each sample by the multi-barcode sequencing approach based on *trnL* biomarker.

Prescribed herbal species (PHS)	DHW.A									DHW.B								
	I1	I2	I3	II1	II2	II3	III1	III2	III3	I1	I2	I3	II1	II2	II3	III1	III2	III3
*Anemone raddeana*	√	√	√	√	√	√	√	√	√	√	√	√	√	√	√	√	√	√
*Angelica sinensis*	√	√	√	√	√	√	√	√	√	√	√	√	√	√	√	√	√	√
*Aquilaria sinensis*	√	√	√	√	√	√	√	√	√	√	√	√	√		√	√	√	√
*Asarum heterotropoides*	√			√	√	√	√	√	√	√	√	√	√	√	√	√	√	√
*Asarum sieboldii*	√	√	√	√	√	√	√	√	√				√	√			√	
*Atractylodes macrocephala*	√	√	√	√	√	√	√	√	√	√	√	√	√	√	√	√	√	√
*Boswellia neglecta*	√	√	√	√	√	√	√	√	√	√	√	√	√	√	√	√	√	√
*Cinnamomum cassia*	√	√	√	√	√	√	√	√	√	√	√	√	√	√	√	√	√	√
*Clematis hexapetala*	√	√	√			√		√									√	
*Coptis chinensis*	√	√	√	√	√	√	√	√	√	√	√	√	√	√	√	√	√	√
*Coptis deltoidea*	√	√	√	√	√	√	√	√	√	√	√	√	√	√	√	√	√	√
*Cyperus rotundus*	√	√			√			√	√									
*Ephedra equisetina*						√			√									
*Ephedra sinica*	√	√	√	√	√	√	√	√	√			√	√	√		√	√	
*Glycyrrhiza uralensis*	√	√	√	√	√	√	√	√	√	√	√	√	√	√	√	√	√	√
*Panax ginseng*	√	√	√	√	√	√	√	√	√	√	√	√	√	√	√	√	√	√
*Pogostemon cablin*	√	√	√	√	√	√	√	√	√	√	√	√	√	√	√	√	√	√
*Rehmannia glutinosa*	√	√	√	√	√	√	√	√	√	√	√	√	√	√	√	√	√	√
*Rheum officinale*	√		√	√	√	√	√	√	√	√		√	√	√	√	√	√	√
*Rheum tanguticum*	√		√	√	√	√	√	√	√	√		√	√	√	√	√	√	√
*Scrophularia ningpoensis*		√					√											
*Scutellaria baicalensis*	√	√	√	√	√	√	√	√	√	√	√	√	√	√	√	√	√	√

The analysis of the sensitivity on BYW and YGW samples based on ITS2 and *trnL* biomarker was shown in Supplementary Tables S4–S7, Supplementary Fig. S2A–B,E–F. Comparing the analysis result of DHW and NJW, NJW only contains one preprocessed PHM (Scutellaria extract), while DHW has seven preprocessed PHMs. Comparing the result with ITS2 biomarker, much fewer species were identified using *trnL* biomarker, which might be caused by DNA extraction, primer specification and the limitation of *trnL* database of Genbank. The three biological replicates from these batches have shown different PHS compositions based on both ITS2 or for *trnL* (Fig. 3, Fig. 4, Tables 3, 4, 5 and 6, Supplementary Figs. S2–S3 and Supplementary Tables S4–S7), which might be potentially caused by DNA extraction, PCR amplification, high-through sequencing technology. The previous research of LDW^13^, YMW^14^, LXW^15^, and JQW^16^ have also shown this phenomenon.

All detected species including PHS, SHS, and CHS of these four TCM preparations (BYW, DHW, NJW, and YGW) were also provided in Supplementary Tables S8–S15. Based on ITS2 biomarker, we detected eight, 25, nine, and six PHMs of BYW, DHW, NJW, and YGW, respectively. The detected proportion of PHMs was 100% for BYW and NJW, followed by DHW (69.4%) and YGW (66.7%). As for *trnL*, five, 18, four, and four PHMs of BYW, DHW, NJW, and YGW were respectively detected. The maximum sensitivity of PHMs was 62.5% among the four TCM preparations in this experiment. The analysis strongly suggested the multi-barcode sequencing approach has a high sensitivity in identifying PHMs of TCM preparations, especially based on the ITS2 dataset.

### Prediction model to predict the quality of TCM preparations

**Figure 5:**
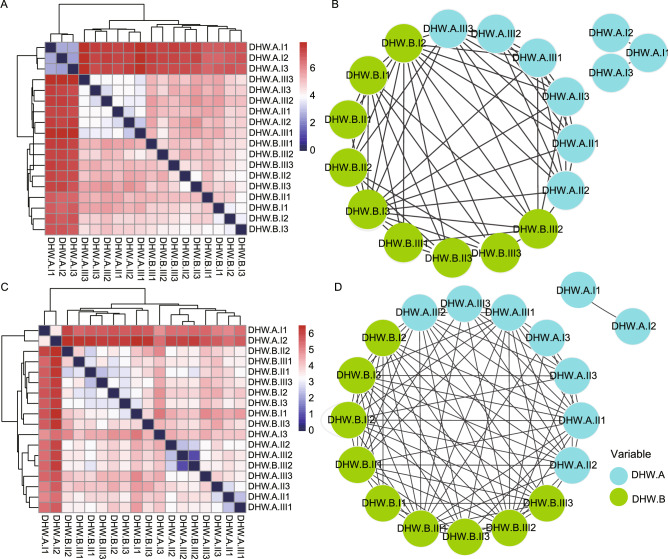
Comparison of the similarity of all DHW samples from intra-/inter-manufacturers based on prescribed herbal materials using Euclidean distances. Heatmap clusters displayed the distance of all samples based on the existence of prescribed herbal species using hierarchical clustering, and network clusters illustrated these differences based on ITS2 (**A**,**B**) and *trnL* (**C**,**D**) sequencing results, respectively. For heatmap (**A**,**C**), which was drawn in R (version 3.5.2) package “pheatmap” (https://cran.rstudio.com/web/packages/pheatmap/index.html), the gradient color bars mean the distance between any two samples, while the red and the blue color depicts the two extreme distances between samples. For network (**B**,**D**) that was visualized in Cytoscape (version 3.7.1; https://cytoscape.org/), each edge represents the distance of any two samples with a distance less than or equal to 5.0 for ITS2 and 4.2 for *trnL*.

Furthermore, we construct a model to differentiate the sample from different which manufacturer and batch the samples were collected. Here, we calculated the Euclidean distances for each pair of samples and then clustered the samples according to their similarity. We took DHW as a case study. The results showed that most DHW samples from manufacturers A and B were clustered together based on both ITS2 (Fig. 5A,B) and *trnL* (Fig. 5C,D) biomarkers, suggesting a high similarity of intra-manufacturer samples. The samples bought from DHW.A.II and DHW.A.III were clustered with DHW.B samples, whereas three samples bought from DHW.A.I, were gathered together, but they were distant from the other samples (Fig. 5A,B). Such separation might be caused by the existence of SHS such as *Senna*, *Amaranthus*, *Glycine,* and CHS such as *Arachis*, *Brassica*, *Solanum,* and *Oryza*. As for NJW (Supplementary Fig. S4), the samples from two manufacturers (A&B) were scattered, based on either ITS2 or *trnL*, while the samples from each manufacturer were clustered together according to the batches (I, II, III), which depicted the high consistency between batches of NJW samples. The cluster analysis results of BYW and YGW samples (Supplementary Figs. S5–S6) showed a clear difference between manufacturers, as well as high similarity within the same manufacturer.

PCA analysis was also performed to explore the consistency of samples from two manufacturers. The samples from DHW.B were clustered more closely than DHW.A based on ITS2 and *trnL* biomarker. Based on ITS2, the samples of DHW from intra-batch were clustered together, while the inter-batches were distributed sparsely. In contrast, based on *trnL*, the samples of DHW.A was dispersed far apart (Supplementary Fig. S7C,D), which suggested that the consistency of DHW.B samples was better than DHW.A. The samples from NJW (Supplementary Fig. S7E,F) were clustered more dispersedly than DHW. The result of BYW and YGW (Supplementary Fig. S7A,B,G,H) was also showed similar results.

**Figure 6:**
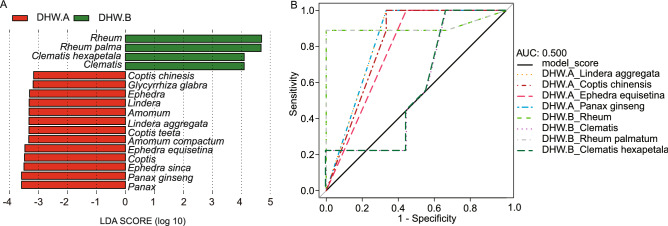
The difference of samples from the two manufacturers (**A**,**B**) could be driven by a few discriminative prescribed herbal species of DHW using ITS2 biomarker. (**A**) The legacy biomarkers selected by LEfSe; (**B**) ROC curves to visualize the MEI score of the legacy biomarkers after removing redundant markers from the two manufacturers.

To investigate the species that drove the difference of samples between manufacturers, LEfSe analysis was conducted for biomarkers discovery. 13 PHS from DHW.A and four from DHW.B were identified as tentative biomarkers (list in Fig. 6A). Through mRMR, five PHS from DHW.A and two PHS of DHW.B were selected. Then, we used the MEI score (formula (1)) to evaluate their performance (Fig. 6B). As the area under ROC curve of *Glycyrrhiza glabra* was less than 0.5, we removed this biomarker from DHW.A. Thus, *Coptis chinensis*, *Ephedra equisetina*, *Lindera aggregate* and *Panax ginseng* were chosen as unique biomarkers of DHW.A. *Rheum palmatum* and *Clematis hexapetala* were selected as representative biomarkers of DHW.B. All of them are of high discrimination power (Fig. 6B), which could be used separately or in combination to differentiate the samples from the two manufacturers. In addition to the ROC analysis, we also used accuracy and F1 score to evaluate the performance of these biomarkers (Supplementary Table S16), which were further supported by the random forest model.

### Comparison of ITS2 and *trnL* on resolutions and sensitivities

Through detecting their PHS, the detected proportion of PHMs was 100% for BYW and NJW, followed by DHW (69.4%) and YGW (66.7%) based on ITS2, while 62.5%, 50%, 44.4% and 44.4% for BYW, DHW, NJW and YGW based on *trnL* datasets respectively (Table 7). The sensitivity of ITS2 was better than that of *trnL* in all TCM preparations, but *trnL* biomarker could also detect the PHS of PHMs that ITS2 couldn’t (*Boswellia neglecta* and *Rehmannia glutinosa*). The union of both biomarkers could detect more PHS, providing a more reliable (as for positive detections) detected result.

**Table 7:** The sensitivity of prescribed herbal materials for four TCM preparations based on ITS2 and *trnL* biomarker.

	ITS2 (%)	*trnL* (%)	Union (%)
BYW	100	62.5	100
DHW	69.4	50	77.8
NJW	100	44.4	100
YGW	66.7	44.4	77.8

Note that the sensitivity was defined as the ratio of the detected prescribed herbal materials and the prescribed herbal materials that could be detected in theory.

**Figure 7:**
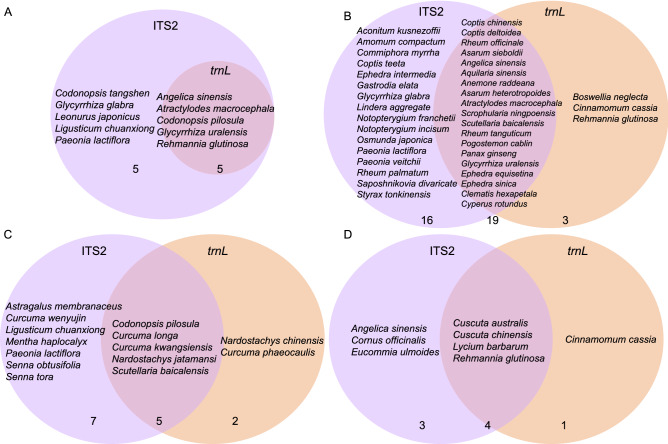
The specific and shared prescribed herbal species of TCM preparations based on ITS2 and *trnL*. (**A**) BYW; (**B**) DHW; (**C**) NJW; (**D**) YGW. The numbers below the Venn diagram mean the number of prescribed herbal species detected based on ITS2, *trnL* only, and the intersection of the two.

As can be observed from the Venn diagram (Fig. 7), all the PHMs of BYW were detected. As for DHW, the union detection result of these two regions was 38 PHS, covering 28 PHMs, which increased the identification efficiency to 77.8%. Similarly, the detection result of *trnL* from NJW preparation was a subset of ITS2 (100% sensitivity). For YGW samples, the union of these two biomarkers increased the sensitivity to 77.8%. This result has also confirmed the high reliability of the multi-barcode sequencing approach. We then compared our result with the previous studies, including JQW, LXW, YMW, and the YYW (Table 8), which indicated the reliability of the multi-barcoding approach. This also suggested that the complexity of biological ingredients of TCM preparation has also negatively affected the detected results.

**Table 8:** Comparison of the sensitivity of prescribed herbal materials through detected prescribed herbal species of TCM preparations.

TCM preparations	All materials	PHMs	The number of detected PHMs			Sensitivity (%)			Undetected PHMs	References
			Biomarker 1	Biomarker 2	Union	Biomarker 1	Biomarker 2	Union		
**BYW**	**9**	**8**	**8 (ITS2)**	**5 (*trnL*)**	**8**	**100**	**62.5**	**100**	**Fulin**	**This work**
**DHW**	**48**	**36**	**25 (ITS2)**	**18 (*trnL*)**	**28**	**69.4**	**50**	**77.8**	Six*	**This work**
JQW	9	9	6 (ITS2)	6 (*psbA-trnH*)	8	66.7	66.7	88.9	Baizhi	^16^
LDW	6	5	5 (ITS2)	4 (*trnL*)	5	100	80	100	–	^13^
LXW	10	10	8 (ITS2)	–	8	80	–	80.0	Zexie Dihuang	^15^
**NJW**	**14**	**9**	**9 (ITS2)**	**4 (*trnL*)**	**9**	**100**	**44.4**	**100**	**–**	**This work**
**YGW**	**10**	**9**	**6 (ITS2)**	**4 (*trnL*)**	**7**	**66.7**	**44.4**	**77.8**	**Fuzi, Shanyao**	**This work**
YMW	4	4	4 (ITS2)	3 (*psbA-trnH*)	4	100	75	100	–	^14^
YYW	4	1	–	1 (*trnL*)	100	–	100	100	–	^2^

Note that we only calculated the sensitivity of prescribed herbal materials of TCM preparation samples bought from manufacturers. The bold contents are the research targets of this work, others are from the previously published studies. *The six undetected PHMs of DHW were Arisaematis rhizome (Tiannanxing), Aucklandiae radix (Muxiang), Olibanum (Ruxiang), Citri reticulatae pericarpium viride (Qingpi), Draconis sanguis (Xuejie), Drynariae rhizome (Gusuibu), Caryophylli flos (Dingxiang), Polygoni multiflori radix (Heshouwu), Puerariae lobatae radix (Gegen).

Though the sensitivity and reliability of the multi-barcode sequencing approach have been demonstrated, the sensitivity of ITS2 and *trnL* is different. ITS2 showed a higher sensitivity than that of *trnL* for PHMs detection, which may cause by more records and a longer conserved region of ITS2. Nevertheless, the role of *trnL* is irreplaceable, as it could complement ITS2 for more reliable identification of the PHMs of TCM preparations, especially for the biological ingredient analysis of DHW and YGW in this work.

## Discussions

As already known to us, herbal materials are the most essential elements in different traditional medicines. An increasing number of papers on DNA-based authentication of single herbs have been published^26,28–33^, while a few applications of the multi-barcode sequencing approach for TCM preparations were reported^13,34–36^.

In this work, the multi-barcode sequencing approach has successfully detected the species (including prescribed, substituted, and contaminated species) in a sample with high sensitivity, indicating the good universality of the method and its potential value for daily TCM supervision. As we could determine the existence of all species in one sample at the species level, these results have indicated an adequate sensitivity of this method in decoding herbal materials of TCM preparations by authenticating their corresponding species. The combination of ITS2 and *trnL* has reached a high sensitivity (minimum: 77.8%, maximum: 100%), highlighting the practical application value and high reliability of this approach. Particularly, the ITS2 exhibited an excellent ability and sensitivity for identifying herbal materials. Although the resolution of *trnL* was lower than that of ITS2, it could also reinforce or complement ITS2 for more reliable results. These results have demonstrated that multi-barcode sequencing was an efficient tool for decoding various TCM preparations’ herbal materials.

For example, for BYW and NJW, all PHMs were detected by authenticating their corresponding PHS. The detected PHS of DHW were 35 (covered 25 PHMs), 22 of them (covered 18 PHMs) based on ITS2 and *trnL*, respectively. The union dataset of ITS2 and *trnL* has boosted the sensitivity increasing from 69.4% to 77.8% for DHW samples. However, six PHMs were not detected in all DHW samples based on either ITS2 or *trnL*. These phenomena might be caused by various preprocessing procedures, such as decocted or stir-fried herbal materials, whose DNA was damaged or degraded. We also note that because of several influencing factors, such as geological location, cultivation conditions, climate, and other conditions, the sensitivity of PHMs of each TCM preparation sample is different.

The multi-barcode sequencing approach could help identify the PHS of PHMs as long as their DNA is not completely damaged. However, in future studies, a deeper and more comprehensive improvement of this multi-barcode sequencing approach still needs to be carried out. A more comprehensive species database was necessary since the reliability of the biological ingredient analysis for TCM preparation largely depends on the reference database^2^. In our future study, we can utilize multiple databases, including the GenBank database, as well as tcmbarcode database^37^, EMBL, DDBJ, and PDB database^2^ to obtain more complete results. Additionally, more biomarker candidates can be considered for assessing the quality of TCM preparation.

Firstly, the multi-barcode sequencing approach could be an attempt to identify the animal materials, because the animal materials still are an important component of TCM and are often combined with medical herbs to exert their pharmacological effects^38^.

Secondly, chemical ingredients analysis based on TLC and HPLC, as well as biological ingredients analysis based on multi-barcode sequencing, are relatively independent but indivisible parts for quality assessments of TCM preparations. TLC and HPLC focus on chemical compounds, while multi-barcode sequencing focuses on species identification. As far as higher sensitivity of species identification was concerned, multi-barcode sequencing technology was superior to HPLC and TLC. However, combining the chemical methods with the DNA barcoding approach, the detection of TCM ingredients might be more comprehensive. Although this thought was initially tested by our group^10^, there is still room for further improvement.

Thirdly, the network pharmacology approach has provided us with a more direct view of the drug–target interactions^39^, which gives us an insight into how to optimize the existing drugs and discover new medicine for satisfying the requirements of overcoming complex diseases. Thus, pharmacological usage should be considered in the QC of TCM preparations, especially for specific usages, such as the mechanism-based QC of YIV-906^40^. This theory has also inspired us to explore the potential treatments of COVID-19 from biological ingredients of TCM preparations^41^. The ingredients such as Glycyrrhizae Radix Et Rhizoma could frequently interact with the target of COVID-19: ACE2^19,41^. Through data-mining, the characteristic of eight biological ingredients of DHW corresponds to the classic Warm disease's symptoms of syndrome differentiation of COVID-19, which might prove effective to treat COVID-19^41^. If combined with public health data, this biological ingredient information might shed more light on the susceptibility of a patient who has taken these TCM preparations, especially those elderly people.

Finally, many herbal medicines are taken orally^42^, undoubtedly exposed to the whole gastrointestinal tract microbiota, which provides sufficiently spatiotemporal opportunities for direct or indirect interactions. For example, berberine, the major pharmacological ingredient of *Coptidis rhizome*^43^, promotes the production of short-chain fatty acid to shift the gut microbiota structure, while the poorly solubilized berberine^44^ was converted into dihydroberberine through a reduction reaction mediated by bacterial nitroreductase, then recovered to the original form after penetrating the intestinal wall tissues^45^, through interactions, the microbial diversity in high-fat diet mice intestines was profoundly decreased^46^.

We believe that these efforts on QC of TCM preparations could joint force and provide much better approaches for the next-generation TCM preparation QC system. Through reshaping the symbiotic microbial composition, we could provide novel therapeutic strategies to accelerate the realization of personalized therapeutics.

Taken together, the multi-barcode sequencing approach was systematically examined with high universality, sensitivity, and reliability. ITS2 shows better identification ability, but *trnL* could detect several PHS or PHMs (such as *Boswellia neglecta* and *Rehmannia glutinosa*) that ITS2 could not, and thus complement ITS2 for more reliable results. Through the multi-barcode sequencing approach, we have detected between 77.8% and 100% PHMs for these representative TCM preparations, which could not be realized through traditional methods, such as morphological and biochemical means. In the future, this approach could assess more diverse sets of TCM preparations, which makes the identification of TCM preparation in a systematic manner, and accelerates the digitization and modernization of the TCM preparation quality control.

## Methods

**Figure 8:**
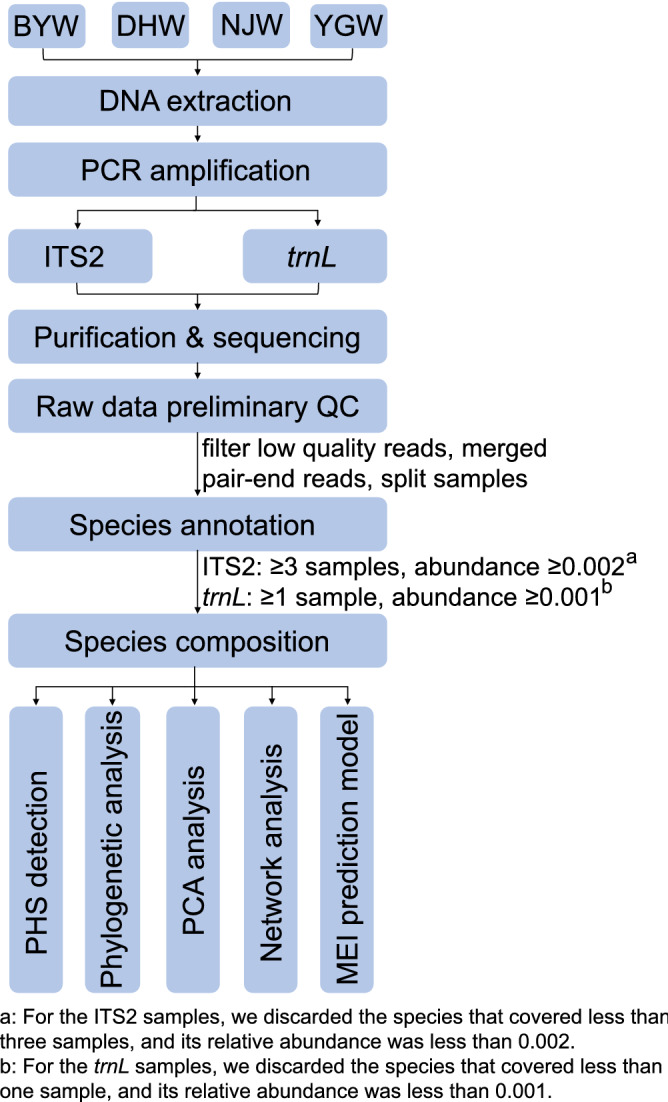
A workflow for TCM preparation analysis procedures. This workflow mainly includes four parts: (1) TCM preparations collection: Baizhen Yimu Wan (BYW), Da Huoluo Wan (DHW), Niuhuang Jiangya Wan (NJW), and Yougui Wan (YGW); (2) Samples preparation and sequencing: DNA extraction, PCR amplification, purification, sequencing; (3) Quality control: raw reads filtering and the species filtering; (4) Species identification and samples comparison: prescribed herbal species (PHS) detection, substituted herbal species (SHS) detection, contaminated herbal species (CHS) detection, the phylogenetic analysis, PCA analysis, network analysis, and Microbial-based environment index (MEI) prediction model for TCM preparations assessment.

The workflow for TCM preparation analysis procedure was also provided in Fig. 8.

### Sample collections

Four TCM preparations, each purchased from two different manufacturers (marked as A and B) with three batches (I, II and III), were collected (Supplementary Table S17). Each batch was implemented with three biological replicates based on ITS2 and *trnL*, respectively. Therefore, 144 samples were used for the subsequent experiment. Here, we gave an example to clarify the naming rule of SampleID: DHW.A.I1 means the DHW sample was bought from manufacturer A, and was one of the three biological replicates (I1) of the first batch (I).

### DNA extraction and quantification

For DNA extraction, we used an optimized cetyl trimethyl ammonium bromide (CTAB) method (TCM-CTAB)^47^. Each sample (1.0 g) was completely dissolved with 0.1 M Tris–HCl, 20 mM EDTA (pH 8.0, 2 mL). Dissolved solution (0.4 mL) was diluted with extraction buffer (0.8 mL) consisting of 2% CTAB; 0.1 M Tris-HC1 (pH 8.0); 20 mM EDTA (pH 8.0); 1.4 M NaCl, and then 100 μL 10% SDS, 10 μL 10 mg/mL Proteinase K (Sigma, MO, USA) and 100 μL *β*-Mercaptoethanol (Amresco, OH, USA) were added and incubated at 65 °C for 1 h with occasional swirling. Protein was removed by extracting twice with an equal volume of phenol:chloroform:isoamyl-alcohol (25:24:1), and once with chloroform: isoamyl-alcohol (24:1). The supernatant was incubated at − 20 °C with 0.6 folds of cold isopropanol for 30 min to precipitate DNA. The precipitate was washed with 75% ethanol, dissolved and diluted to 10 ng/μL with TE buffer, and then used as a PCR amplification template. DNA concentration was quantified on Qubit2.0 Fluorometer.

### DNA amplification and DNA sequencing

The PCR amplification was performed in a 50 μL reaction mixture that contain 1 μL of DNA extracted from TCM preparations, 10.0 μL of 5 × PrimeSTAR buffer (Mg^2+^ plus) (TaKaRa), 2.5 μL of 10 μM dNTPs (TaKaRa), 0.5 μL each of forward and reverse primers (10 μM), 2.5 μL dimethylsulfoxide (DMSO) and 0.5 μL PrimeSTAR HS DNA Polymerase (Takara, 2.5 U/μL). For amplification and sequencing of the ITS2 region, the forward primers S2F^12^ and the reverse primer ITS4^48^ (Supplementary Table S18) with seven bp MID tags (Supplementary Table S19) were designed for PCR amplification. PCR reactions were implemented as follows: pre-denaturation at 95 °C for five min, then 10 cycles made up of 95 °C for 30 s and 62 °C for 30 s with ramping of − 1 °C per cycle, followed by 72 °C for 30 s, next followed by 40 cycles of 95 °C for 30 s, 55 °C for 30 s and 72 °C for 30 s; the procedure ended with 72 °C for 10 min. For the *trnL* region, the forward primers *trnL*-c and the reverse primer *trnL*-h with 7 bp MID tags (Supplementary Table S19) were also designed for PCR amplification. The PCR reactions were carried out according to the conditions: pre-denaturation at 95 °C for 5 min, 10 cycles made up of 95 °C for 30 s and 62 °C for 30 s with ramping of − 1 °C per cycle, followed by 72 °C for 30 s; then followed by 40 cycles of 95 °C for 30 s, 58 °C for 30 s and 72 °C for 30 s; the procedure ended with 72 °C for 10 min. For a better amplification effect, touchdown PCR^48,49^ was carried out. The PCR products were electrophoresed on 1% agarose gel and purified with QIAquick Gel Extraction kit (QIAGEN). The DNA concentration was quantified on Qubit2.0 Fluorometer. After removing one *trnL*-marked BYW specimen failed to be amplified, which was potentially caused by severe PCR inhibition, and one ITS2-marked YGW sample that failed to be built in the next-generation sequencing library preparation, 142 samples (Supplementary Table S20) were sent for Illumina MiSeq PE300 pair-end sequencing. The raw sequencing data for TCM preparation samples were deposited to the NCBI SRA database with accession number PRJNA562480.

### Sequencing data analysis procedure and software configuration

We first used the FastQC (version 0.11.7) with default parameters to evaluate the quality of the sequencing reads. Reads from the same sample were assembled using the QIIME script ‘join_paired_end.py’. Then we used the ‘extract_barcodes.py’ to extract the double-end barcodes from all reads, and the ‘split_libraries_fastq.py’ was used to split the sample according to their barcodes (Supplementary Table S19) from the mixed sequencing data. We also used its ‘-q 20 --max_bad_run_length 3 --min_per_read_length_fraction 0.75 --max_barcode_errors 0-barcode_type 7’ parameters to preliminarily filter the low-quality sequences, then Cutadapt software (version 1.14) was used to remove the primers (Supplementary Table S18) and adapter from all samples.

These reads of all samples were QCed by MOTHUR^50^ (version 1.41.0). Per reads of ITS2 whose length is < 150 bp or > 510 bp and the reads of *trnL* whose length is < 75 bp were removed. After that, we discarded the sequence whose average quality score was below 20 in every five bp sliding window along with the whole reads. The sequences that contained ambiguous base call (N), homopolymers over eight bases or primers mismatched, incorrect barcodes, were also removed from ITS2 and *trnL* datasets.

To assign taxonomical annotation for each sequence, we used the BLASTN (e-value = 1e−10) to search in ITS2 and *trnL* databases based on GenBank^51^, respectively. Among all results, we first chose the PHS with the highest score, else we selected the top-scored species as the target species for the sequence. In addition, we also manually searched all PHS of PHMs in all samples. Then, we discarded the corresponding species of ITS2 and *trnL* sequences with relative abundance below 0.002 and 0.001, respectively. Rarefaction analysis was performed with R^52^ (version 3.5.2) using the “vegan” package to evaluate the sequencing depth of TCM preparation samples (Code 1 for rarefaction analysis in Supplementary materials).

As for the composition of PHS, we used the heatmap with gradient color using R (version 3.5.2) package “pheatmap” (https://cran.rstudio.com/web/packages/pheatmap/index.html) to illustrate the composition of the PHS based on their relative abundance in each sample, and used 0 (not detected) and 1 (detected) to describe the existence status of PHS in each sample. For the species phylogeny, we first obtained their phylogenetic trees from NCBI Taxonomy (https://www.ncbi.nlm.nih.gov/Taxonomy/CommonTree/wwwcmt.cgi). Then, we visualized these phylogenetic trees in iTOL (https://itol.embl.de/). The inner and outer circles mean the relative abundance of this species in manufacturers A and B, respectively. Each circle has three colors, which represent the relative abundance of species in three batches. The distance between any two samples was calculated by Euclidean distance based on the existence of prescribed herbal species. We then visualized the sample-sample distance in the heatmap with hierarchical clustering in R (version 3.5.2) package “pheatmap” (https://cran.rstudio.com/web/packages/pheatmap/index.html). By using the sample as node and the distance of any two samples as edge, we built a network cluster for each TCM preparation and visualized it in Cytoscape (version 3.7.1)^53^ based on ITS2 and *trnL*, respectively. Principal component analysis (PCA) analysis was also performed to detect the difference between two manufacturers (Code 2 for PCA analysis in Supplementary materials). We also used the LDA Effect Size (LEfSe)^54^ to select legacy biomarkers, and then performed feature selection using minimum redundancy maximum relevance feature selection (mRMR)^55^ to select the most discriminative biomarkers. To ensure the selected biomarkers’ performance, we also used an integrated index defined as microbiome-based environment index (MEI) score. That is, the ratio of AUr(S_*i*_) and BUr(S_*j*_), defined as:

1 $$\mathrm{MEI \; score}=\frac{{\sum }_{i=1}^{n}\mathrm{AUr}\left({\mathrm{S}}_{i}\right)}{\sum_{j=1}^{n}\mathrm{BUr}({\mathrm{S}}_{j})},$$

where AUr(S_*i*_) and BUr(S_*j*_) represent the relative abundance of the *i*th and or *j*th selected biomarkers for the two manufacturers A and B through LEfSe and mRMR, respectively.

The receiver operating characteristic (ROC) curve^56^ analysis was applied to visualize the classification effectiveness (MEI score) of the biomarker selected from different manufacturers. We also used the random forest (“randomforest” package in R) to evaluate the selected biomarkers’ performance, which took the accuracy, F1 score and ROC into consideration. The data and parameter settings were detailedly described in our previous study^57^.

### Terminology and abbreviation definitions

The prescribed herbal materials were defined as the herbal materials of a TCM preparation recorded in ChP, abbreviated to PHMs.

The prescribed herbal species (abbreviated to PHS) were the original species of PHMs, any one of them should be considered as the PHS.

The species that have the same genus with PHS, was defined as substituted herbal species (SHS). The species excluded from the two above species was considered as the contaminated herbal species (CHS).

For easier understanding the abbreviations used in this work, we took one TCM preparation, YGW, as an example, as shown in Table 2. The information for other TCM preparations was shown in Supplementary Table S3. We have also provided detailed information about the animal and mineral materials for the four TCM preparations in Supplementary Table S1.

The universality was a measurement to evaluate how the multi-barcode sequencing approach could apply to a broad scope of TCM preparations. The four representative TCM preparations were selected for this purpose.

The sensitivity was defined as the ratio of the number of detected PHMs over the number of PHMs that could be identified in theory, that is,

$${\text{Sensitivity }} = \, ({\text{the}}\,{\text{ number}}\,{\text{ of}}\,{\text{detected}}\,{\text{PHMs}})/ \, \left( {{\text{the}}\,{\text{number}}\,{\text{of}}\,{\text{PHMs}}\,{\text{ could }}\,{\text{be}}\,{\text{detected}}\,{\text{in}}\,{\text{theory}}} \right).$$

The reliability was defined as the number of detectable PHMs from the TCM preparations by the multi-barcode sequencing approach. The larger number of detectable PHMs, the better reliability.

## Supplementary Information

Supplementary Information.

## Data Availability

The data that support the findings of this study are available from NCBI SRA database with accession number PRJNA562480 (https://www.ncbi.nlm.nih.gov/bioproject/PRJNA562480/).
