# Supplementary material for: Decoding herbal materials of TCM preparations with the multi-barcode sequencing approach
Source: Sci Rep. 2022 Apr 9;12:5988. doi: 10.1038/s41598-022-09979-z (PMC8994760; doi:10.1038/s41598-022-09979-z)
Supplement: Supplementary file 1 — Supplementary Information. [file 41598_2022_9979_MOESM1_ESM.docx]

**Supplementary Materials**

**Decoding herbal materials of TCM preparations with the multi-barcode sequencing approach**

Qi Yao^1,#^, Xue Zhu^1,#^, Maozhen Han^1^, Chaoyun Chen^1^, Wei Li^2^, Hong Bai^1,*^, Kang Ning^1,*^

^1^*Key Laboratory of Molecular Biophysics of the Ministry of Education, Hubei Key Laboratory of Bioinformatics and Molecular-imaging, Center of AI Biology, Department of Bioinformatics and Systems Biology, College of Life Science and Technology, Huazhong University of Science and Technology,* *Wuhan, Hubei* *430074, China*

^2^ *Faculty of Pharmaceutical Sciences, Toho University, Tokyo, 1438540, Japan*

^#^ These authors contributed equally to this work

* Correspondence should be addressed to Hong Bai: baihong@hust.edu.cn and Kang Ning: ningkang@hust.edu.cn

**This additional file includes:**

Figure S1-S7

Tables S1-S20

**References**

**Figures**

**
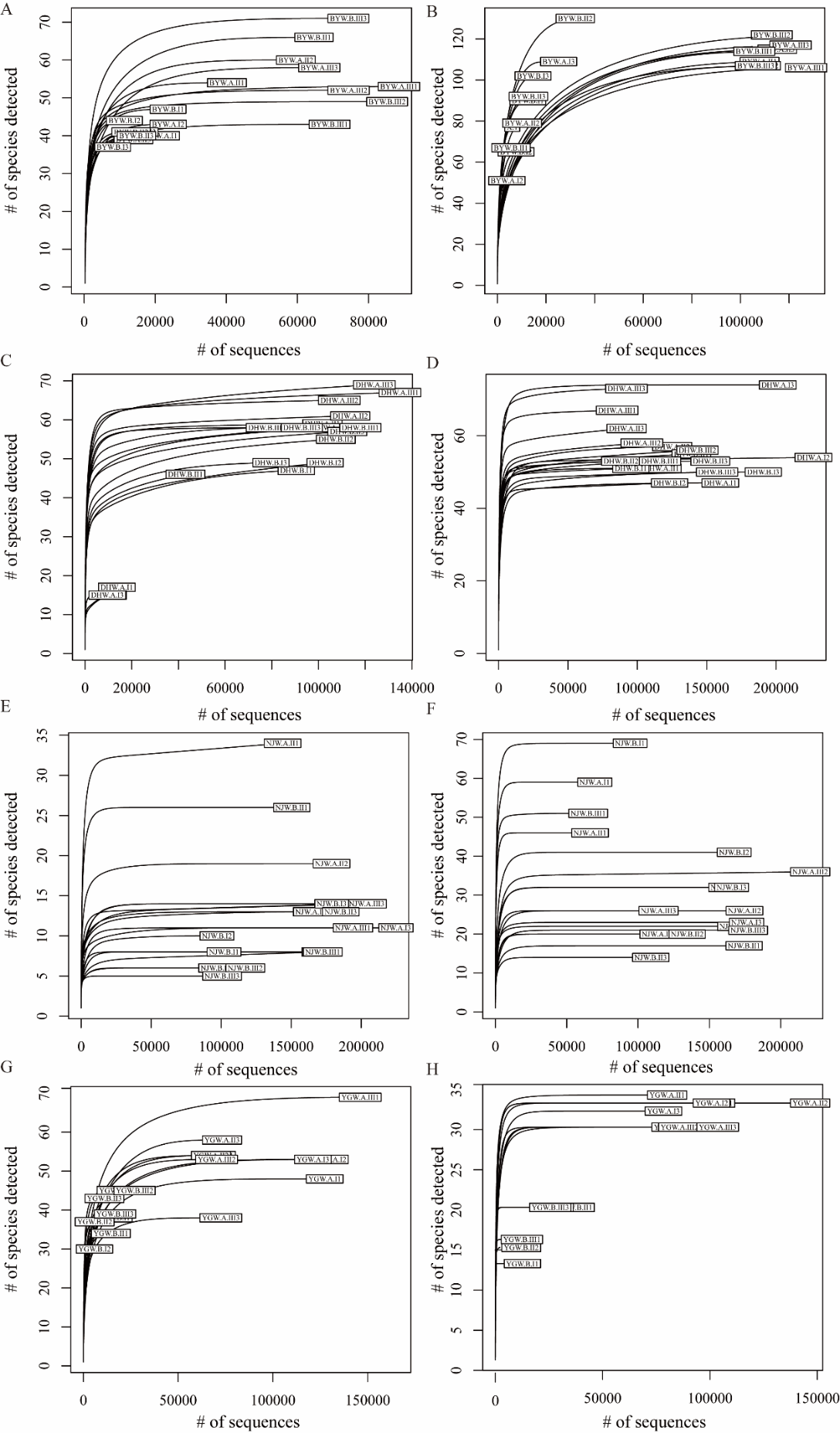
**

**Figure S1. Rarefaction carves of sequencing depth sampled from two manufactures for per TCM preparation based on ITS2 and *trnL*.** (A) BYW based on ITS2; (B) BYW based on *trnL*; (C) DHW based on ITS2; (D) DHW based on *trnL*. (E) NJW based on ITS2. (F) NJW based on trnL; (G) YGW based on ITS2; (H) YGW based on *trnL*. The result shows all samples’ sequencing depth were saturation.

**
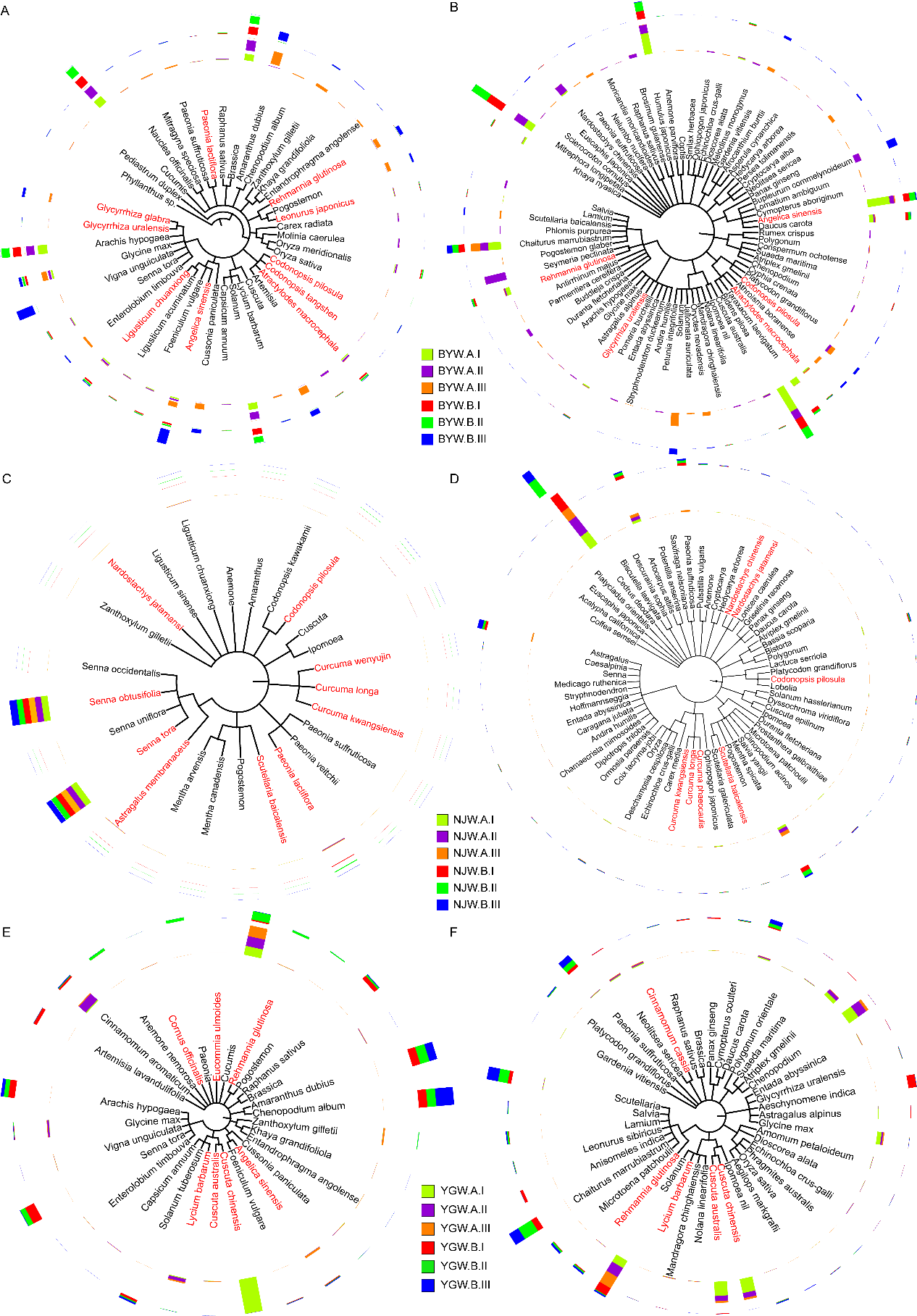
**

**Figure S2. Phylogenetic analysis of the representative species that had at least 0.1% relative abundance for TCM preparation samples.** (A) BYW samples based on ITS2; (B) BYW samples based on *trnL*; (C) NJW samples based on ITS2; (D) NJW samples based on *trnL*; (E) YGW samples based on ITS2; (F) YGW samples based on *trnL*. The phylogenetic trees of species are visualized in iTOL (<https://itol.embl.de/>). The word marked in red means the prescribed herbal species, and the colorful bar means the average relative abundances of species across the three batches from the two manufacturers (A&B).


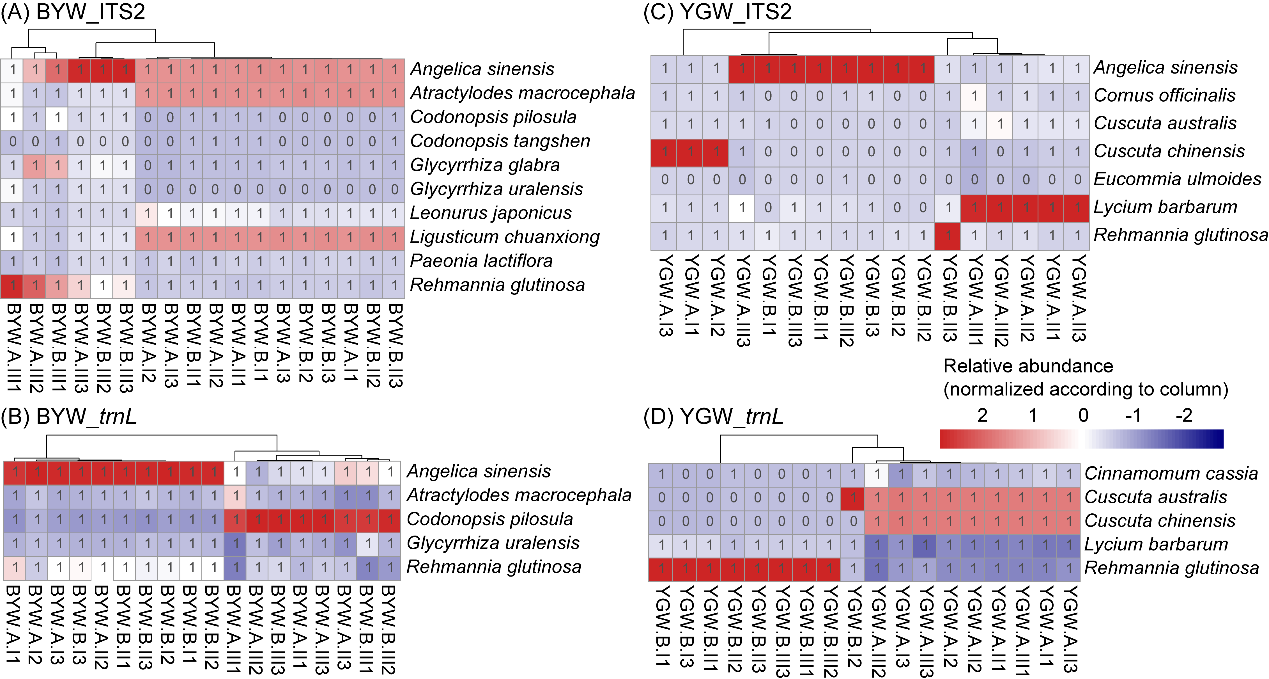


**Figure S3. The distribution of prescribed herbal species (PHS) detected in each sample from BYW and YGW preparations.** (A) The detected PHS in BYW samples based on ITS2. (B) The detected PHS in BYW samples based on *trnL*. (C) The detected PHS in YGW samples based on ITS2. (D) The detected PHS in YGW samples based on *trnL*. Note that each column represents a sample, and each row represents a PHS. In the heatmap that was drawn in R (version 3.5.2) package “pheatmap” (https://cran.rstudio.com/web/packages/pheatmap/index.html), the color represents the relative abundance of PHS (normalized according to column) detected in this sample. The number “1” represents the PHS was detected in this sample, while “0” represents the PHS was not detected.

**
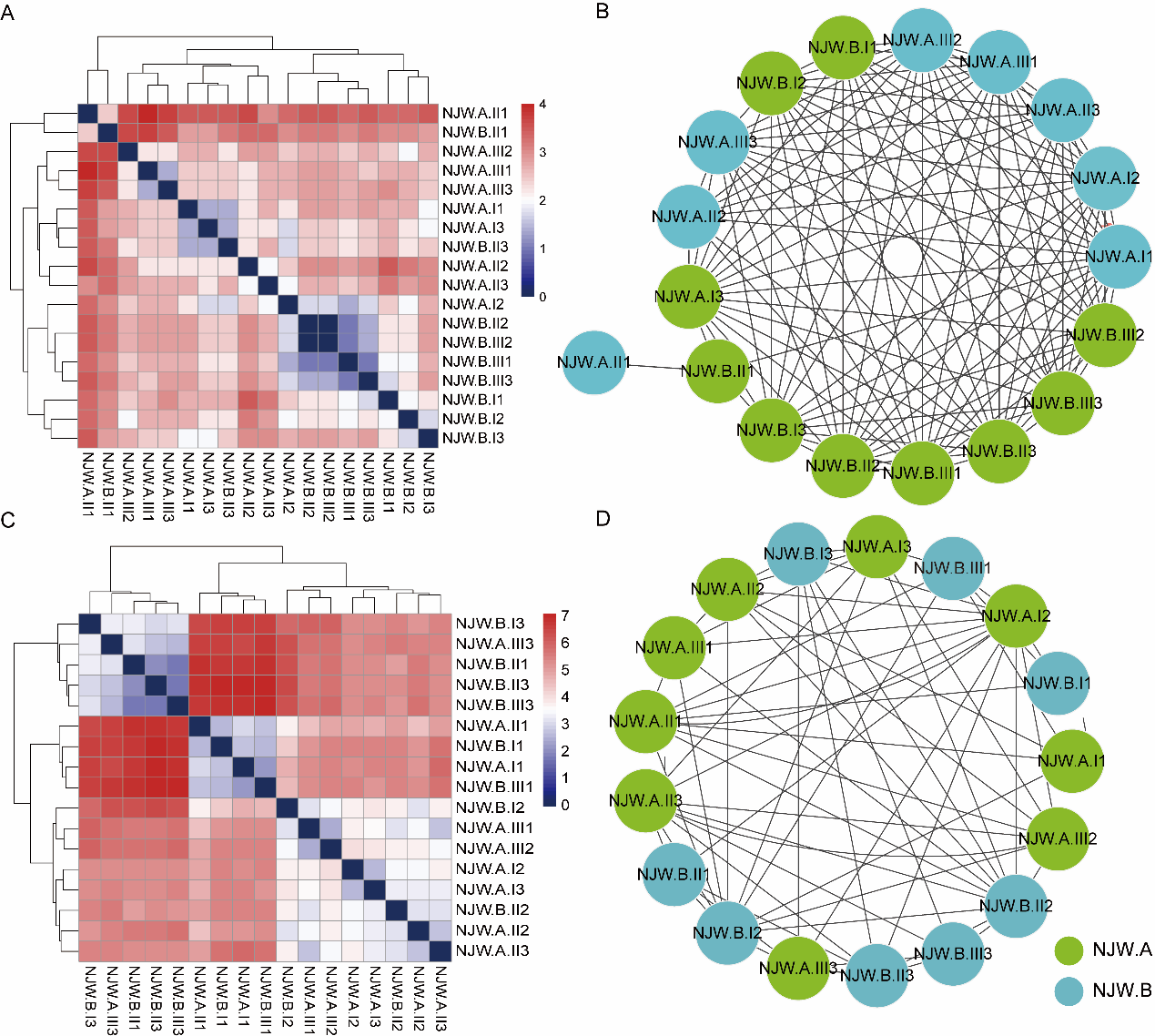
**

**Figure S4. Comparison of the similarity of all NJW samples from intra-/inter-manufacturers based on prescribed herbal materials using Euclidean distances.** Heatmap clusters displayed the distance of all samples based on the existence of prescribed herbal species using hierarchical clustering, and network clusters illustrated these differences in Cytoscape based on ITS2 (A and B) and *trnL* (C and D) sequencing results. For heatmap (A & C) that was drawn in R (version 3.5.2) package “pheatmap” (https://cran.rstudio.com/web/packages/pheatmap/index.html), the gradient color bars mean the distance between any two samples, while the red and the blue color depicts the two extreme distances between samples. For network (B & D) that was visualized in Cytoscape (version 3.7.1; https://cytoscape.org/), each edge represents the distance of any two samples with a distance less than 2.9 for ITS2 and 3.9 for *trnL*.

**
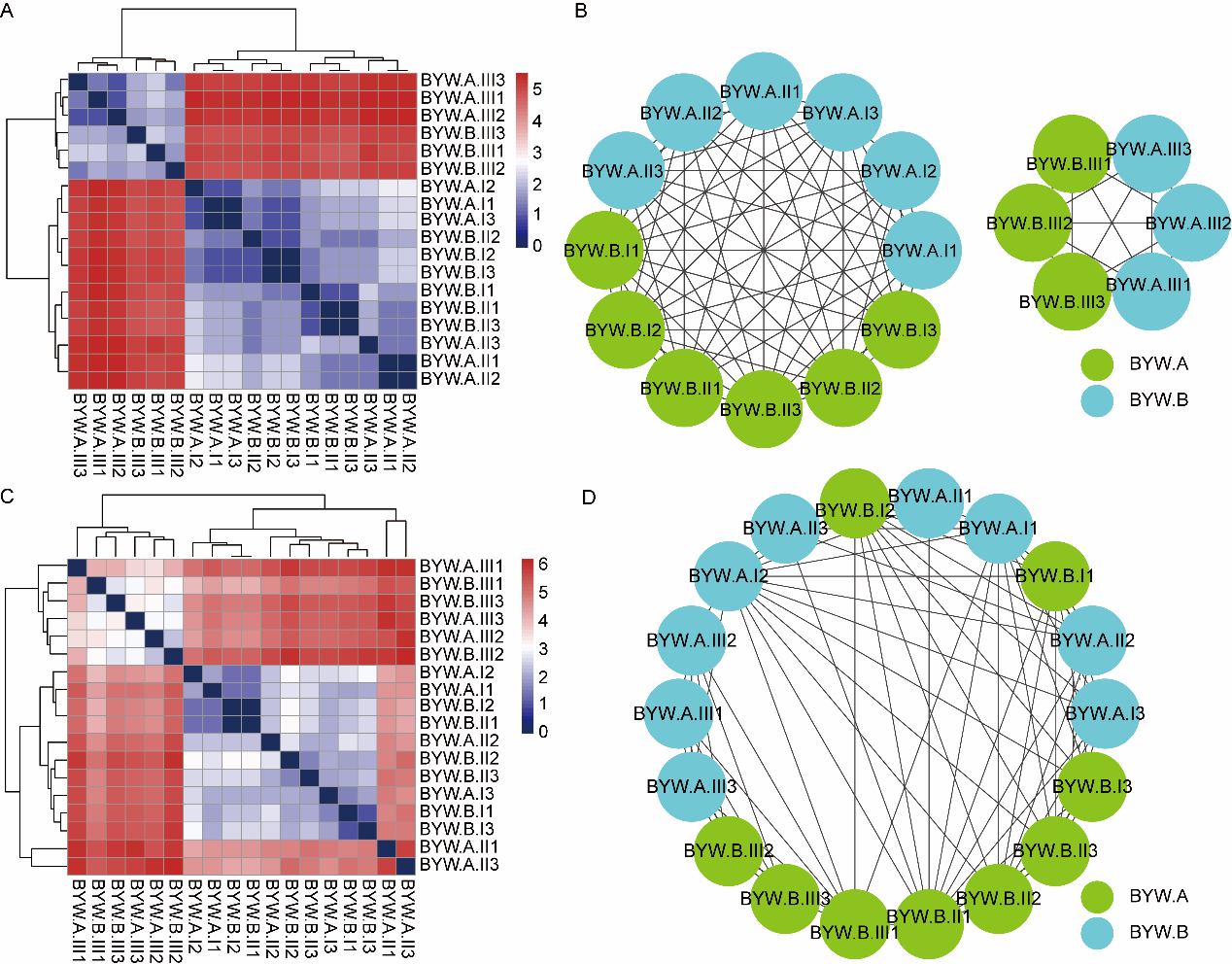
**

**Figure S5. Comparison of the similarity of all BYW samples from intra-/inter-manufacturers based on prescribed herbal materials using Euclidean distances.** Heatmap clusters displayed the distance of all samples based on the existence of prescribed herbal species using hierarchical clustering, and network clusters illustrated these differences in Cytoscape based on ITS2 (A and B) and *trnL* (C and D) sequencing results. For heatmap (A & C), which was drawn in R (version 3.5.2) package “pheatmap” (https://cran.rstudio.com/web/packages/pheatmap/index.html), the gradient color bars mean the distance between any two samples, while the red and the blue color depicts the two extreme distances between samples. For network (B & D) that was visualized in Cytoscape (version 3.7.1; https://cytoscape.org/), each edge represents the distance of any two samples with a distance less than 2.5 for ITS2 and 4.6 for *trnL*.

**
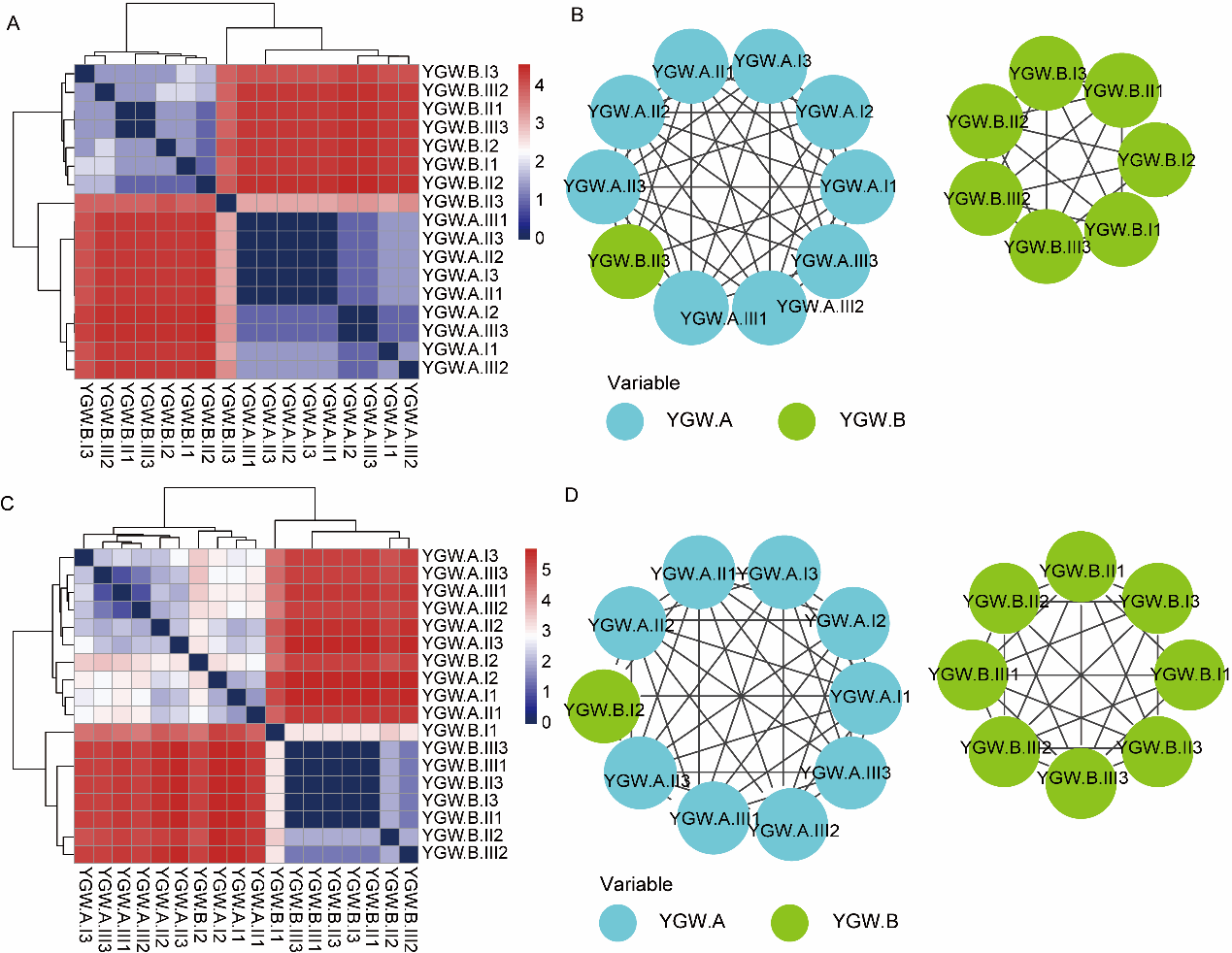
**

**Figure S6. Comparison of the similarity of all YGW samples from intra-/inter-manufacturers based on prescribed herbal materials using Euclidean distances.** Heatmap clusters displayed the distance of all samples based on the existence of prescribed herbal species using hierarchical clustering, and network clusters illustrated these differences in Cytoscape based on ITS2 (A and B) and *trnL* (C and D) sequencing results. For heatmap (A & C), which was drawn in R (version 3.5.2) package “pheatmap” (https://cran.rstudio.com/web/packages/pheatmap/index.html), the gradient color bars mean the distance between any two samples, while the red and the blue color depicts the two extreme distances between samples. For network (B & D) that was visualized in Cytoscape (version 3.7.1; https://cytoscape.org/), each edge represents the distance of any two samples with a distance less than 3.2 for ITS2 and 3.2 for *trnL*.

**
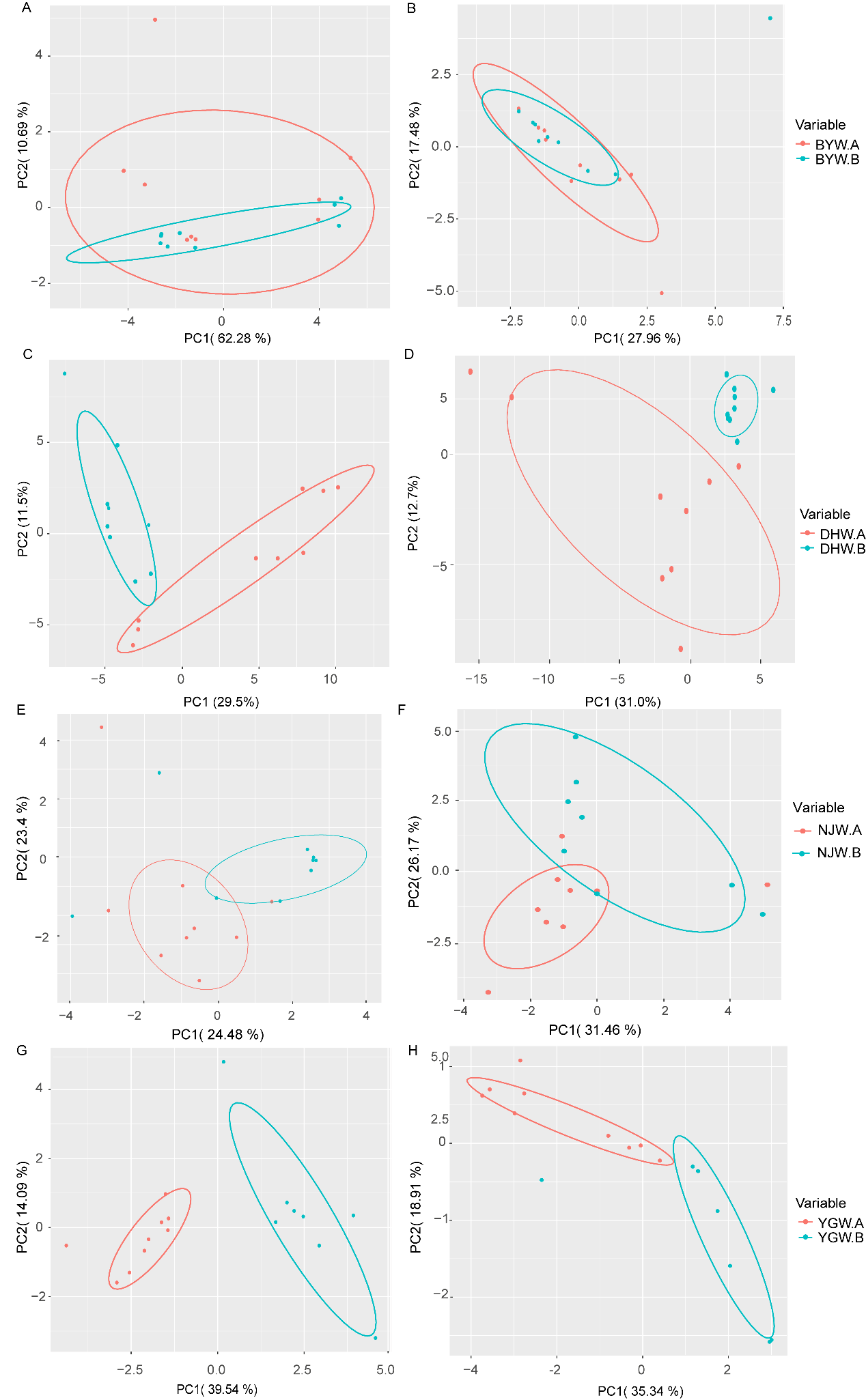
**

**Figure S7. PCA analysis for assessing the consistency of the samples from intra-/inter manufacturer comparison.** (A) The samples of BYW based on ITS2; (B) The samples of BYW based on *trnL*; (C) The samples of DHW based on ITS2; (D) The samples of DHW based on *trnL*; (E) The samples of NJW based on ITS2 biomarker; (F) The samples of NJW based on *trnL* biomarker; (G) The samples of YGW based on ITS2; (H) The samples of YGW based on *trnL*.

**Tables**

**Table S1. The information about the prescribed animal and mineral materials and their corresponding prescribed species for the four TCM preparations recorded in Chinese pharmacopoeia.**

| **TCM preparation** | **Ingredient type** | **Prescribed materials** | **Prescribed species** |
| --- | --- | --- | --- |
| Da Huoluo Wan (DHW) | animal (10) | Agkistrodon | *Agkistrodon acutus* |
|  |  | Zaocys | *Zaocys dhumnades* |
|  |  | Bombyx Batryticatus | *Bombyx mori* |
|  |  | Testudinis Carapax Et Plastrum | *Chinemys reevesii* |
|  |  | Panfhera Pardus L. | *Panthera pardus* |
|  |  |  | *Panthera uncia* |
|  |  |  | *Neofelis nebulosa* |
|  |  | Pheretima | *Pheretima aspergillum* |
|  |  |  | *Pheretima vulgaris* |
|  |  |  | *Pheretima guillelmi* |
|  |  |  | *Pheretima pectinifera* |
|  |  | Powerdered Buffalo Horn Extract | *Bubalus bubalts* |
|  |  | Manmade Moschus | *Moschus berezovskii* |
|  |  |  | *Moschus sifanicus* |
|  |  |  | *Moschus moschiferus* |
|  |  | Scorpio | *Buthus martensii* |
|  |  | Bovis Calculus Sativus | *Bos taurus domesticus* |
|  | mineral (2) | Borneolum Syntheticum | *Borneolum syntheticum* |
|  |  | Colophonium | *Pinus palustris* |
|  |  |  | *Pinus caribaea* |
|  |  |  | *Pinus taeda* |
| Niuhuang Jiangya Wan (NJW) | animal (4) | Saigae tataricae cornu | *Saiga tatarica* |
|  |  | Margarita | *Pteria martensii* |
|  |  |  | *Hyriopsis cumingii* |
|  |  |  | *Cristaria plicata* |
|  |  | Powerdered buffalo horn extract | *Bubalus bubalts* |
|  |  | Bovis calculus artifactus | *Bos taurus domesticus* |
|  | mineral (1) | Borneolum syntheticum | *Borneolum syntheticum* |
| Yougui Wan (YGW) | animal (1) | Cervi cornuscolla | *Antlet glue* |

Note: Bazhen Yimu Wan (BYW) only contains herbal materials, and YGW does not contain mineral material.

**Table S2. The length of sequences obtained from BYW, DHW, NJW and YGW samples.**

| **SampleID** | **ITS2** | | | ***trnL*** | | | **SampleID** | **ITS2** | | | ***trnL*** | | |
| --- | --- | --- | --- | --- | --- | --- | --- | --- | --- | --- | --- | --- | --- |
|  | **Max** | **Min** | **Avg** | **Max** | **Min** | **Avg** |  | **Max** | **Min** | **Avg** | **Max** | **Min** | **Avg** |
| BYW.A.I1 | 510 | 151 | 373 | 274 | 143 | 172 | NJW.A.I1 | 422 | 162 | 373 | 298 | 129 | 169 |
| BYW.A.I2 | 461 | 174 | 363 | 274 | 145 | 173 | NJW.A.I2 | 469 | 223 | 373 | 572 | 145 | 171 |
| BYW.A.I3 | 510 | 178 | 377 | 367 | 143 | 173 | NJW.A.I3 | 434 | 223 | 373 | 275 | 96 | 172 |
| BYW.A.II1 | 509 | 181 | 365 | 379 | 146 | 165 | NJW.A.II1 | 486 | 182 | 370 | 274 | 138 | 170 |
| BYW.A.II2 | 506 | 151 | 365 | 367 | 139 | 172 | NJW.A.II2 | 414 | 204 | 372 | 453 | 145 | 173 |
| BYW.A.II3 | 505 | 181 | 364 | 277 | 100 | 166 | NJW.A.II3 | 449 | 168 | 372 | 448 | 142 | 172 |
| BYW.A.III1 | 448 | 157 | 356 | 378 | 107 | 165 | NJW.A.III1 | 414 | 273 | 372 | 569 | 145 | 172 |
| BYW.A.III2 | 411 | 187 | 355 | 319 | 89 | 165 | NJW.A.III2 | 396 | 273 | 372 | 571 | 104 | 173 |
| BYW.A.III3 | 411 | 175 | 354 | 373 | 141 | 165 | NJW.A.III3 | 396 | 252 | 372 | 468 | 128 | 172 |
| BYW.B.I1 | 486 | 181 | 366 | 275 | 144 | 172 | NJW.B.I1 | 388 | 272 | 372 | 474 | 138 | 169 |
| BYW.B.I2 | 509 | 151 | 373 | 274 | 144 | 173 | NJW.B.I2 | 508 | 169 | 372 | 476 | 144 | 172 |
| BYW.B.I3 | 509 | 274 | 376 | 282 | 106 | 172 | NJW.B.I3 | 438 | 206 | 372 | 471 | 142 | 173 |
| BYW.B.II1 | 506 | 178 | 366 | 275 | 144 | 173 | NJW.B.II1 | 510 | 256 | 371 | 453 | 143 | 173 |
| BYW.B.II2 | 490 | 195 | 368 | 275 | 144 | 173 | NJW.B.II2 | 405 | 158 | 373 | 571 | 145 | 173 |
| BYW.B.II3 | 505 | 181 | 368 | 337 | 111 | 173 | NJW.B.II3 | 434 | 271 | 372 | 275 | 145 | 172 |
| BYW.B.III1 | 411 | 262 | 356 | 367 | 107 | 166 | NJW.B.III1 | 445 | 225 | 374 | 273 | 141 | 168 |
| BYW.B.III2 | 490 | 167 | 354 | 346 | 144 | 165 | NJW.B.III2 | 380 | 272 | 373 |  |  |  |
| BYW.B.III3 | 411 | 152 | 354 | 275 | 77 | 166 | NJW.B.III3 | 405 | 272 | 373 | 275 | 145 | 173 |
| DHW.A.I1 | 510 | 204 | 383 | 277 | 131 | 171 | YGW.A.I1 | 496 | 224 | 353 | 358 | 144 | 168 |
| DHW.A.I2 | 510 | 181 | 376 | 428 | 83 | 171 | YGW.A.I2 | 457 | 183 | 353 | 354 | 143 | 169 |
| DHW.A.I3 | 509 | 165 | 377 | 557 | 143 | 169 | YGW.A.I3 | 407 | 229 | 353 | 354 | 143 | 193 |
| DHW.A.II1 | 492 | 176 | 361 | 345 | 108 | 170 | YGW.A.II1 | 411 | 223 | 354 | 356 | 144 | 171 |
| DHW.A.II2 | 487 | 176 | 361 | 325 | 143 | 167 | YGW.A.II2 | 411 | 223 | 354 | 457 | 141 | 167 |
| DHW.A.II3 | 508 | 176 | 361 | 273 | 141 | 170 | YGW.A.II3 | 455 | 233 | 355 | 276 | 144 | 165 |
| DHW.A.III1 | 503 | 156 | 361 | 377 | 140 | 170 | YGW.A.III1 | 489 | 182 | 354 | 277 | 100 | 166 |
| DHW.A.III2 | 508 | 253 | 362 | 352 | 143 | 169 | YGW.A.III2 | 483 | 183 | 352 | 338 | 106 | 166 |
| DHW.A.III3 | 508 | 159 | 360 | 377 | 131 | 169 | YGW.A.III3 | 445 | 273 | 353 | 275 | 143 | 163 |
| DHW.B.I1 | 492 | 156 | 355 | 360 | 143 | 176 | YGW.B.I1 | 457 | 180 | 376 | 274 | 145 | 173 |
| DHW.B.I2 | 510 | 151 | 357 | 374 | 144 | 170 | YGW.B.I2 | 480 | 181 | 366 | 563 | 107 | 167 |
| DHW.B.I3 | 468 | 227 | 355 | 345 | 144 | 167 | YGW.B.I3 | 443 | 170 | 356 | 367 | 143 | 173 |
| DHW.B.II1 | 497 | 183 | 354 | 536 | 144 | 171 | YGW.B.II1 | 468 | 221 | 378 | 364 | 87 | 172 |
| DHW.B.II2 | 473 | 197 | 355 | 568 | 131 | 170 | YGW.B.II2 | 442 | 151 | 363 | 371 | 107 | 172 |
| DHW.B.II3 | 503 | 236 | 356 | 367 | 122 | 168 | YGW.B.II3 | 497 | 150 | 361 | 274 | 116 | 172 |
| DHW.B.III1 | 510 | 239 | 357 | 363 | 143 | 171 | YGW.B.III1 | |  |  | 274 | 145 | 172 |
| DHW.B.III2 | 501 | 171 | 359 | 365 | 116 | 171 | YGW.B.III2 | 443 | 166 | 379 | 378 | 104 | 171 |
| DHW.B.III3 | 497 | 167 | 359 | 351 | 131 | 170 | YGW.B.III3 | 447 | 181 | 377 | 274 | 143 | 172 |

Note Max: the maximum length in one sample; min: the minimum length in one sample; Avg: the average length in one sample.

**Table S3. The prescribed herbal materials and their corresponding prescribed herbal species of (a) Bazhen Yimu Wan (BYW), (b) Da Huoluo Wan (DHW), (c) Niuhuang Jiangya Wan (NJW) recorded in Chinese pharmacopoeia.**

**(a) Bazhen Yimu Wan (BYW)**

| **TCM preparation** | **Prescribed herbal material (PHM)** | **Prescribed herbal species (PHS)** |
| --- | --- | --- |
| Bazhen Yimu Wan (BYW) | Angelica sinensis (oliv.) diels. | *Angelica sinensis* |
|  | Atractylodis macrocephalae | *Atractylodes macrocephala* |
|  | Codonopsis radix | *Codonopsis pilosula* |
|  |  | *Codonopsis tangshen* |
|  | Glycyrrhizae radix et rhizoma | *Glycyrrhiza glabra* |
|  |  | *Glycyrrhiza inflate* |
|  |  | *Glycyrrhiza uralensis* |
|  | Leonuri herba | *Leonurus japonicus* |
|  | Chuanxiong rhizoma | *Ligusticum chuanxiong* |
|  | Paeoniae radix alba | *Paeonia lactiflora* |
|  | Rehmanniae radix praeparata | *Rehmannia glutinosa* |

Note: Un-prescribed herbal materials mainly include the substituted herbal species (SHS) and contaminated herbal species (CHS).

**(b) Da Huoluo Wan (DHW)**

| **TCM preparation** | **Prescribed herbal material (PHM)** | **Prescribed herbal species (PHS)** |
| --- | --- | --- |
| Da Huoluo Wan (DHW) | Aconiti kusnezoffii radix | *Aconitum kusnezoffii* |
|  | Alpinia katsumadai hayata | *Amomum compactum* |
|  |  | *Amomum kravanh* |
|  | Anemones raddeanae rhizoma | *Anemone raddeana* |
|  | Angelica sinensis (oliv.) diels. | *Angelica sinensis* |
|  | Aquilariae lignum resinatum | *Aquilaria sinensis* |
|  | Arisaematis rhizoma | *Arisaema amurense* |
|  |  | *Arisaema erubescens* |
|  |  | *Arisaema heterophyllum* |
|  | Asari radix et rhizoma | *Asarum heterotropoides* |
|  |  | *Asarum sieboldii* |
|  | Atractylodis macrocephalae | *Atractylodes macrocephala* |
|  | Rhizoma | *Aucklandia lappa* |
|  | Olibanum | *Boswellia bhaw-dajiana* |
|  |  | *Boswellia carterii* |
|  |  | *Boswellia neglecta* |
|  | Cinnamomum cassia Presl. | *Cinnamomum cassia* |
|  | Citri reticulatae pericarpium viride | *Citrus reticulate* |
|  | Clematidis radix et rhizoma | *Clematis chinensis* |
|  |  | *Clematis hexapetala* |
|  |  | *Clematis manshurica* |
|  | Myrrha | *Commiphora molmol* |
|  |  | *Commiphora myrrha* |
|  | Coptidis rhizoma | *Coptis chinensis* |
|  |  | *Coptis deltoidea* |
|  |  | *Coptis teeta* |
|  | Cyperi rhizoma | *Cyperus rotundus* |
|  | Draconis sanguis | *Daemonorops draco* |
|  | Drynariae rhizoma | *Drynaria fortune* |
|  | Dryopteris crassirhizoma nakai | *Dryopteris crassirhizoma* |
|  |  | *Osmunda japonica* |
|  | Ephedrae herba | *Ephedra equisetina* |
|  |  | *Ephedra intermedia* |
|  |  | *Ephedra sinica* |
|  | Caryophylli flos | *Eugenia caryophyllata* |
|  | Gastrodiae rhizoma | *Gastrodia elata* |
|  | Glycyrrhizae radix et rhizoma | *Glycyrrhiza glabra* |
|  |  | *Glycyrrhiza inflate* |
|  |  | *Glycyrrhiza uralensis* |
|  | Linderae radix | *Lindera aggregate* |
|  | Notopterygii rhizoma et radix | *Notopterygium franchetii* |
|  |  | *Notopterygium incisum* |
|  | Dryopteris crassirhizoma nakai | *Osmunda japonica* |
|  | Paeoniae radix rubra | *Paeonia lactiflora* |
|  |  | *Paeonia veitchii* |
|  | Ginseng radix et rhizoma rubra | *Panax ginseng* |
|  | Pogostemonis herba | *Pogostemon cablin* |
|  | Polygoni multiflori radix | *Polygonum multiflorum* |
|  | Puerariae lobatae radix | *Pueraria lobata* |
|  |  | *Pueraria thomsonii* |
|  | Rehmanniae radix praeparata | *Rehmannia glutinosa* |
|  | Rhei radix et rhizoma | *Rheum officinale* |
|  |  | *Rheum palmatum* |
|  |  | *Rheum tanguticum* |
|  | Saposhnikoviae radix | *Saposhnikovia divaricate* |
|  | Scrophulariae radix | *Scrophularia ningpoensis* |
|  | Scutellariae radix | *Scutellaria baicalensis* |
|  | Benzoinum | *Styrax tonkinensis* |

Note: Un-prescribed herbal materials mainly include the substituted herbal species (SHS) and contaminated herbal species (CHS).

**(c) Niuhuang Jiangya Wan (NJW)**

| **TCM preparation** | **Prescribed herbal material (PHM)** | **Prescribed herbal species (PHS)** |
| --- | --- | --- |
| Niuhuang Jiangya Wan (NJW) | Astragali radix | *Astragalus membranaceus* |
|  | Codonopsis radix | *Codonopsis pilosula* |
|  |  | *Codonopsis tangshen* |
|  | Curcumae radix | *Curcuma kwangsiensis* |
|  |  | *Curcuma longa* |
|  |  | *Curcuma phaeocaulis* |
|  |  | *Curcuma wenyujin* |
|  | Chuanxiong rhizoma | *Ligusticum chuanxiong* |
|  | Menthae haplocalycis herba | *Mentha haplocalyx* |
|  | Nardostachyos radix et rhizoma | *Nardostachys chinensis* |
|  |  | *Nardostachys jatamansi* |
|  | Paeoniae radix alba | *Paeonia lactiflora* |
|  | Scutellariae radix | *Scutellaria baicalensis* |
|  | Cassiae semen | *Senna obtusifolia* |
|  |  | *Senna tora* |

Note: Un-prescribed herbal materials mainly include the substituted herbal species (SHS) and contaminated herbal species (CHS).

**Table S4. Prescribed herbal species for BYW preparation and their presence in each sample by multi-barcode sequencing approach based on ITS2 biomarker.**

| **Prescribed herbal species (PHS)** | **BYW.A** | | | | | | | | |  | **BYW.B** | | | | | | | | |
| --- | --- | --- | --- | --- | --- | --- | --- | --- | --- | --- | --- | --- | --- | --- | --- | --- | --- | --- | --- |
|  | **I1** | **I2** | **I3** | **II1** | **II2** | **II3** | **III1** | **III2** | **III3** |  | **I1** | **I2** | **I3** | **II1** | **II2** | **II3** | **III1** | **III2** | **III3** |
| *Angelica sinensis* | √ | √ | √ | √ | √ | √ | √ | √ | √ |  | √ | √ | √ | √ | √ | √ | √ | √ | √ |
| *Atractylodes macrocephala* | √ | √ | √ | √ | √ | √ | √ | √ | √ |  | √ | √ | √ | √ | √ | √ | √ | √ | √ |
| *Codonopsis pilosula* |  |  |  | √ | √ |  | √ | √ | √ |  | √ |  |  | √ |  | √ | √ | √ | √ |
| *Codonopsis tangshen* |  |  |  | √ | √ |  |  |  |  |  | √ |  |  | √ |  | √ | √ |  |  |
| *Glycyrrhiza glabra* | √ |  | √ | √ | √ | √ | √ | √ | √ |  | √ | √ | √ | √ | √ | √ | √ | √ | √ |
| *Glycyrrhiza uralensis* |  |  |  |  |  |  | √ | √ | √ |  |  |  |  |  |  |  | √ | √ | √ |
| *Leonurus japonicus* | √ | √ | √ | √ | √ | √ | √ | √ | √ |  | √ | √ | √ | √ | √ | √ | √ | √ | √ |
| *Ligusticum chuanxiong* | √ | √ | √ | √ | √ | √ | √ | √ | √ |  | √ | √ | √ | √ | √ | √ | √ | √ | √ |
| *Paeonia lactiflora* | √ | √ | √ | √ | √ | √ | √ | √ | √ |  | √ | √ | √ | √ | √ | √ | √ | √ | √ |
| *Rehmannia glutinosa* | √ | √ | √ | √ | √ | √ | √ | √ | √ |  | √ | √ | √ | √ | √ | √ | √ | √ | √ |

**Table S5. Prescribed herbal species for BYW preparation and their presence in each sample by multi-barcode sequencing approach based on *trnL* biomarker.**

| **Prescribed herbal species (PHS)** | **BYW.A** | | | | | | | | |  | **BYW.B** | | | | | | | | |
| --- | --- | --- | --- | --- | --- | --- | --- | --- | --- | --- | --- | --- | --- | --- | --- | --- | --- | --- | --- |
|  | **I1** | **I2** | **I3** | **II1** | **II2** | **II3** | **III1** | **III2** | **III3** |  | **I1** | **I2** | **I3** | **II1** | **II2** | **II3** | **III1** | **III2** | **III3** |
| *Angelica sinensis* | √ | √ | √ | √ | √ | √ | √ | √ | √ |  | √ | √ | √ | √ | √ | √ | √ | √ | √ |
| *Atractylodes macrocephala* | √ | √ | √ | √ | √ | √ | √ | √ | √ |  | √ | √ | √ | √ | √ | √ | √ | √ | √ |
| *Codonopsis pilosula* | √ | √ | √ | √ | √ | √ | √ | √ | √ |  | √ | √ | √ | √ | √ | √ | √ | √ | √ |
| *Glycyrrhiza uralensis* | √ | √ | √ | √ | √ | √ | √ | √ | √ |  | √ | √ | √ | √ | √ | √ | √ | √ | √ |
| *Rehmannia glutinosa* | √ | √ | √ | √ | √ | √ | √ | √ | √ |  | √ | √ | √ | √ | √ | √ | √ | √ | √ |

**Table S6. Prescribed herbal species for YGW preparation and their presence in each sample by multi-barcode sequencing approach based on ITS2 biomarker.**

| **Prescribed herbal species (PHS)** | **YGW.A** | | | | | | | | |  | **YGW.B** | | | | | | | |
| --- | --- | --- | --- | --- | --- | --- | --- | --- | --- | --- | --- | --- | --- | --- | --- | --- | --- | --- |
|  | **I1** | **I2** | **I3** | **II1** | **II2** | **II3** | **III1** | **III2** | **III3** |  | **I1** | **I2** | **I3** | **II1** | **II2** | **II3** | **III2** | **III3** |
| *Angelica sinensis* | √ | √ | √ | √ | √ | √ | √ | √ | √ |  | √ | √ | √ | √ | √ | √ | √ | √ |
| *Cornus officinalis* | √ | √ | √ | √ | √ | √ | √ | √ | √ |  |  |  | √ |  |  | √ | √ |  |
| *Cuscuta australis* | √ | √ | √ | √ | √ | √ | √ | √ | √ |  | √ |  |  |  |  | √ |  |  |
| *Cuscuta chinensis* | √ | √ | √ | √ | √ | √ | √ |  | √ |  |  |  |  |  |  | √ |  |  |
| *Eucommia ulmoides* |  |  |  |  |  |  |  |  |  |  |  |  |  |  |  |  | √ |  |
| *Lycium barbarum* | √ | √ | √ | √ | √ | √ | √ | √ | √ |  |  |  | √ | √ |  | √ | √ | √ |
| *Rehmannia glutinosa* | √ | √ | √ | √ | √ | √ | √ | √ | √ |  | √ | √ | √ | √ | √ | √ | √ | √ |

**Table S7. Prescribed herbal species for YGW preparation and their presence in each sample by multi-barcode sequencing approach based on *trnL* biomarker.**

| **Prescribed herbal species (PHS)** | **YGW.A** | | | | | | | | |  | **YGW.B** | | | | | | | | |
| --- | --- | --- | --- | --- | --- | --- | --- | --- | --- | --- | --- | --- | --- | --- | --- | --- | --- | --- | --- |
|  | **I1** | **I2** | **I3** | **II1** | **II2** | **II3** | **III1** | **III2** | **III3** |  | **I1** | **I2** | **I3** | **II1** | **II2** | **II3** | **III1** | **III2** | **III3** |
| *Cinnamomum cassia* | √ | √ | √ | √ | √ | √ | √ | √ | √ |  | √ | √ |  |  | √ |  |  | √ |  |
| *Cuscuta australis* | √ | √ | √ | √ | √ | √ | √ | √ | √ |  |  | √ |  |  |  |  |  |  |  |
| *Cuscuta chinensis* | √ | √ | √ | √ | √ | √ | √ | √ | √ |  |  |  |  |  |  |  |  |  |  |
| *Lycium barbarum* | √ | √ | √ | √ | √ | √ | √ | √ | √ |  | √ | √ | √ | √ | √ | √ | √ | √ | √ |
| *Rehmannia glutinosa* | √ | √ | √ | √ | √ | √ | √ | √ | √ |  | √ | √ | √ | √ | √ | √ | √ | √ | √ |

**Table S8. All detected species including PHS, SHS and CHS of the BYW preparations based on ITS2.**

| **Detected species** | **BYW.A** | | | | | | | | |  | **BYW.B** | | | | | | | | |
| --- | --- | --- | --- | --- | --- | --- | --- | --- | --- | --- | --- | --- | --- | --- | --- | --- | --- | --- | --- |
|  | **I1** | **I2** | **I3** | **II1** | **II2** | **II3** | **III1** | **III2** | **III3** |  | **I1** | **I2** | **I3** | **II1** | **II2** | **II3** | **III1** | **III2** | **III3** |
| ***Angelica sinensis*** | √ | √ | √ | √ | √ | √ | √ | √ | √ |  | √ | √ | √ | √ | √ | √ | √ | √ | √ |
| ***Atractylodes macrocephala*** | √ | √ | √ | √ | √ | √ | √ | √ | √ |  | √ | √ | √ | √ | √ | √ | √ | √ | √ |
| ***Codonopsis pilosula*** |  |  |  | √ | √ |  | √ | √ | √ |  | √ |  |  | √ |  | √ | √ | √ | √ |
| ***Codonopsis tangshen*** |  |  |  | √ | √ |  |  |  |  |  | √ |  |  | √ |  | √ | √ |  |  |
| ***Glycyrrhiza glabra*** | √ |  | √ | √ | √ | √ | √ | √ | √ |  | √ | √ | √ | √ | √ | √ | √ | √ | √ |
| ***Glycyrrhiza uralensis*** |  |  |  |  |  |  | √ | √ | √ |  |  |  |  |  |  |  | √ | √ | √ |
| ***Leonurus japonicus*** | √ | √ | √ | √ | √ | √ | √ | √ | √ |  | √ | √ | √ | √ | √ | √ | √ | √ | √ |
| ***Ligusticum chuanxiong*** | √ | √ | √ | √ | √ | √ | √ | √ | √ |  | √ | √ | √ | √ | √ | √ | √ | √ | √ |
| ***Paeonia lactiflora*** | √ | √ | √ | √ | √ | √ | √ | √ | √ |  | √ | √ | √ | √ | √ | √ | √ | √ | √ |
| ***Rehmannia glutinosa*** | √ | √ | √ | √ | √ | √ | √ | √ | √ |  | √ | √ | √ | √ | √ | √ | √ | √ | √ |
| *Ligusticum acuminatum* | √ | √ | √ | √ | √ | √ |  |  |  |  | √ | √ | √ | √ | √ | √ |  |  |  |
| *Paeonia suffruticosa* |  |  |  |  |  |  | √ | √ | √ |  |  |  |  |  |  |  | √ | √ | √ |
| *Amaranthus dubius* |  |  |  | √ | √ | √ | √ | √ | √ |  |  |  |  | √ | √ | √ | √ | √ | √ |
| *Arachis hypogaea* | √ | √ | √ | √ | √ | √ |  |  |  |  | √ | √ | √ | √ | √ | √ |  |  |  |
| *Artemisia* |  |  |  | √ | √ | √ |  |  |  |  | √ | √ | √ | √ | √ | √ |  |  |  |
| *Brassica* | √ | √ | √ | √ | √ | √ |  |  |  |  | √ | √ | √ | √ | √ | √ |  |  |  |
| *Capsicum annuum* | √ | √ | √ | √ | √ | √ |  |  |  |  | √ | √ | √ | √ | √ | √ |  |  |  |
| *Carex radiata* |  |  |  | √ | √ | √ |  |  |  |  |  |  |  |  |  |  |  |  |  |
| *Chenopodium album* |  |  |  |  |  |  | √ | √ | √ |  |  |  |  |  |  |  |  |  |  |
| *Cucumis* | √ | √ | √ | √ | √ | √ |  |  |  |  | √ | √ | √ | √ | √ | √ |  |  |  |
| *Cuscuta* |  |  |  |  |  |  | √ | √ | √ |  |  |  |  |  |  |  | √ | √ | √ |
| *Cussonia paniculata* |  |  |  |  |  |  | √ | √ | √ |  |  |  |  |  |  |  | √ | √ | √ |
| *Entandrophragma angolense* |  |  |  |  |  |  | √ | √ | √ |  |  |  |  |  |  |  | √ | √ | √ |
| *Enterolobium timbouva* |  |  |  |  |  |  | √ | √ | √ |  |  |  |  |  |  |  | √ | √ |  |
| *Foeniculum vulgare* | √ | √ | √ | √ | √ | √ |  |  |  |  | √ | √ | √ | √ | √ | √ |  |  |  |
| *Glycine max* | √ | √ | √ | √ | √ | √ | √ | √ | √ |  | √ | √ | √ | √ | √ | √ | √ | √ | √ |
| *Khaya grandifoliola* |  |  |  |  |  |  | √ | √ | √ |  |  |  |  |  |  |  | √ | √ |  |
| *Lycium barbarum* |  |  |  |  |  |  | √ | √ | √ |  |  |  |  |  |  |  | √ | √ | √ |
| *Mitragyna speciosa* |  |  |  |  |  |  | √ | √ | √ |  |  |  |  |  |  |  | √ | √ | √ |
| *Molinia caerulea* |  |  |  | √ | √ | √ |  |  |  |  |  |  |  |  |  |  |  |  |  |
| *Nauclea officinalis* |  |  |  |  |  |  | √ | √ |  |  |  |  |  |  |  |  | √ |  | √ |
| *Oryza meridionalis* | √ | √ | √ | √ | √ | √ |  |  |  |  | √ | √ | √ | √ | √ | √ |  |  |  |
| *Oryza sativa* |  |  |  |  |  |  | √ |  |  |  |  |  |  |  |  |  |  |  |  |
| *Pediastrum duplex* |  |  |  |  |  |  | √ | √ | √ |  |  |  |  |  |  |  |  | √ | √ |
| *Phyllanthus sp.* |  |  |  |  |  |  | √ | √ | √ |  |  |  |  |  |  |  | √ | √ | √ |
| *Pogostemon* |  |  |  |  |  |  | √ | √ | √ |  |  |  |  |  |  |  | √ | √ | √ |
| *Raphanus sativus* | √ | √ | √ | √ | √ | √ |  |  |  |  | √ | √ | √ | √ | √ | √ |  |  |  |
| *Senna tora* | √ | √ | √ | √ | √ | √ |  |  |  |  | √ | √ | √ | √ | √ | √ | √ | √ |  |
| *Solanum* | √ | √ | √ | √ | √ | √ |  |  |  |  | √ | √ | √ | √ | √ | √ |  |  |  |
| *Vigna unguiculata* |  |  |  |  |  |  | √ | √ | √ |  |  |  |  |  |  |  | √ | √ | √ |
| *Zanthoxylum gilletii* |  |  |  |  |  |  | √ | √ | √ |  |  |  |  |  |  |  | √ | √ | √ |

Note: The bold font of detected species means the prescribed herbal materials.

**Table S9. All detected species including PHS, SHS and CHS of the BYW preparations based on *trnL*.**

| **Detected species** | BYW.A | | | | | | | | |  | BYW.B | | | | | | | | |
| --- | --- | --- | --- | --- | --- | --- | --- | --- | --- | --- | --- | --- | --- | --- | --- | --- | --- | --- | --- |
|  | I1 | I2 | I3 | II1 | II2 | II3 | III1 | III2 | III3 |  | I1 | I2 | I3 | II1 | II2 | II3 | III1 | III2 | III3 |
| ***Angelica sinensis*** | √ | √ | √ | √ | √ | √ | √ | √ | √ |  | √ | √ | √ | √ | √ | √ | √ | √ | √ |
| ***Atractylodes macrocephala*** | √ | √ | √ | √ | √ | √ | √ | √ | √ |  | √ | √ | √ | √ | √ | √ | √ | √ | √ |
| ***Codonopsis pilosula*** | √ | √ | √ | √ | √ | √ | √ | √ | √ |  | √ | √ | √ | √ | √ | √ | √ | √ | √ |
| ***Glycyrrhiza uralensis*** | √ | √ | √ | √ | √ | √ | √ | √ | √ |  | √ | √ | √ | √ | √ | √ | √ | √ | √ |
| ***Rehmannia glutinosa*** | √ | √ | √ | √ | √ | √ | √ | √ | √ |  | √ | √ | √ | √ | √ | √ | √ | √ | √ |
| *Paeonia suffruticosa* |  |  |  |  |  |  | √ | √ | √ |  |  |  |  |  |  |  |  | √ | √ |
| *Afrocanthium burttii* |  |  |  |  |  |  | √ |  |  |  |  |  |  |  |  |  |  |  |  |
| *Andira humilis* |  |  |  |  |  | √ |  |  |  |  |  |  |  |  |  |  |  |  |  |
| *Anemone parviflora* |  |  |  |  |  |  |  |  |  |  |  |  |  |  |  |  |  | √ | √ |
| *Antirrhinum majus* |  |  |  |  |  |  |  |  |  |  |  |  |  |  | √ | √ |  |  |  |
| *Arachis hypogaea* |  |  |  | √ |  |  |  |  |  |  |  |  |  |  |  |  |  |  |  |
| *Asperula cynanchica* |  |  |  |  |  | √ |  |  |  |  |  |  |  |  |  |  |  |  |  |
| *Astragalus alpinus* |  |  |  |  |  |  |  | √ | √ |  |  |  |  |  |  |  |  | √ | √ |
| *Athroisma boranense* |  |  |  |  | √ | √ |  |  |  |  |  |  | √ |  |  |  |  |  |  |
| *Atriplex gmelinii* |  |  |  |  |  |  | √ | √ | √ |  |  |  |  |  |  |  | √ | √ | √ |
| *Bidens pilosa* |  |  | √ | √ |  |  |  |  |  |  | √ |  | √ |  | √ |  |  |  |  |
| *Brosimum guianense* | √ | √ | √ |  | √ |  |  |  |  |  | √ | √ | √ | √ | √ | √ |  |  |  |
| *Buddleja crispa* |  |  |  | √ |  |  |  |  |  |  |  |  |  |  |  |  |  |  |  |
| *Bupleurum commelynoideum* |  |  |  | √ |  |  |  |  |  |  |  |  |  |  |  |  |  |  |  |
| *Chaiturus marrubiastrum* |  |  |  |  |  | √ |  |  | √ |  |  |  |  |  |  |  | √ | √ | √ |
| *Chenopodium* | √ | √ | √ | √ | √ | √ | √ | √ | √ |  | √ | √ | √ | √ | √ | √ | √ | √ | √ |
| *Coptis* |  |  |  | √ | √ |  |  |  |  |  |  |  |  |  |  |  |  |  |  |
| *Corispermum ochotense* |  |  |  |  |  |  | √ |  |  |  |  |  |  |  |  |  |  |  |  |
| *Cryptocarya alba* |  |  |  |  |  | √ |  |  |  |  |  |  |  |  |  |  |  |  |  |
| *Cuscuta australis* |  |  |  |  |  |  | √ | √ | √ |  |  |  |  |  |  |  | √ | √ | √ |
| *Cymopterus aboriginum* |  |  | √ |  | √ |  |  |  |  |  | √ |  | √ |  | √ | √ |  |  |  |
| *Cyphia crenata* |  |  |  |  |  | √ |  |  |  |  |  |  |  |  |  |  |  |  |  |
| *Daucus carota* |  |  |  |  |  |  | √ | √ | √ |  |  |  |  |  |  |  | √ | √ | √ |
| *Dioscorea alata* | √ | √ | √ |  | √ |  |  | √ | √ |  | √ | √ | √ | √ | √ | √ | √ | √ |  |
| *Duranta fletcheriana* | √ |  | √ |  | √ | √ |  |  |  |  | √ | √ | √ | √ | √ | √ |  |  |  |
| *Echinochloa crus-galli* |  |  |  |  |  |  | √ | √ |  |  |  |  |  |  |  |  |  | √ |  |
| *Entada abyssinica* |  |  |  |  |  |  | √ | √ | √ |  |  |  |  |  |  |  | √ | √ | √ |
| *Euscaphis japonica* |  |  |  | √ |  | √ |  |  |  |  |  |  |  |  |  |  |  |  |  |
| *Gardenia vitiensis* |  |  |  |  |  |  | √ |  | √ |  |  |  |  |  |  |  | √ | √ | √ |
| *Glycine max* |  |  |  |  |  | √ | √ | √ | √ |  |  |  |  |  |  |  | √ | √ | √ |
| *Hedycarya arborea* |  |  |  | √ |  |  | √ | √ | √ |  |  |  |  |  |  |  |  | √ |  |
| *Hoffmannseggia burchellii* |  |  |  | √ |  |  |  |  |  |  |  |  |  |  |  |  | √ | √ |  |
| *Humulus japonicus* |  |  | √ |  | √ | √ |  |  |  |  |  |  |  |  | √ | √ |  |  |  |
| *Ipomoea nil* | √ | √ | √ | √ | √ |  |  | √ |  |  | √ | √ | √ | √ | √ | √ | √ | √ | √ |
| *Jaltomata auriculata* |  |  |  | √ |  |  |  |  |  |  |  |  |  |  | √ |  |  |  |  |
| *Khaya nyasica* |  |  |  |  |  |  | √ |  |  |  |  |  |  |  |  |  |  |  | √ |
| *Lamium* | √ |  | √ |  | √ |  |  |  |  |  | √ |  | √ |  | √ | √ |  |  |  |
| *Lomatium ambiguum* |  |  | √ |  |  |  |  |  |  |  | √ |  | √ |  | √ | √ |  |  |  |
| *Mandragora chinghaiensis* |  |  |  |  |  |  | √ | √ | √ |  |  |  |  |  |  |  | √ | √ | √ |
| *Melodinus monogynus* |  |  |  |  |  |  |  |  |  |  | √ |  | √ |  | √ | √ |  |  |  |
| *Mitrephora longipetala* |  |  |  |  |  | √ | √ |  |  |  |  |  |  |  |  |  |  |  |  |
| *Moricandia moricandioides* | √ | √ | √ | √ | √ | √ |  |  |  |  | √ | √ | √ | √ | √ | √ |  |  |  |
| *Nardostachys chinensis* |  |  |  | √ |  |  |  |  |  |  |  |  |  |  |  |  |  |  |  |
| *Nelumbo nucifera* | √ |  |  | √ |  |  |  |  |  |  | √ |  | √ |  | √ |  |  |  |  |
| *Neolitsea sericea* |  |  |  |  |  |  | √ | √ | √ |  |  |  |  |  |  |  | √ | √ | √ |
| *Nolana linearifolia* |  |  |  |  |  |  | √ | √ | √ |  |  |  |  |  |  |  | √ | √ | √ |
| *Ophiopogon japonicus* |  |  |  |  |  | √ |  |  |  |  |  |  |  |  |  |  |  |  |  |
| *Oryctes nevadensis* |  |  |  |  |  | √ |  |  |  |  |  |  |  |  |  |  |  |  |  |
| *Panax ginseng* |  |  |  |  |  |  |  |  | √ |  |  |  |  |  |  |  |  | √ |  |
| *Parmentiera cereifera* |  |  |  |  |  |  | √ |  |  |  |  |  |  |  |  |  |  |  |  |
| *Persea tolimanensis* |  |  |  | √ |  |  |  |  |  |  |  |  |  |  |  |  |  |  |  |
| *Petunia integrifolia* |  |  |  | √ |  |  |  |  |  |  |  |  |  |  |  |  |  |  |  |
| *Phlomis purpurea* |  |  |  |  |  | √ |  |  |  |  |  |  |  |  |  |  |  |  |  |
| *Platycodon grandiflorus* |  |  |  |  |  |  |  | √ |  |  |  |  |  |  |  |  |  | √ | √ |
| *Pogostemon glaber* |  |  |  |  |  |  | √ | √ | √ |  |  |  |  |  |  |  |  | √ |  |
| *Polygonum* |  |  |  | √ |  |  | √ | √ |  |  |  |  |  |  |  |  |  | √ |  |
| *Raphanus sativus* |  |  |  | √ |  |  |  |  |  |  |  |  |  |  |  |  |  |  |  |
| *Rumex crispus* |  |  |  | √ |  |  |  |  |  |  |  |  |  |  |  |  |  |  |  |
| *Salvia* |  |  |  |  |  | √ |  |  |  |  |  |  |  |  |  |  | √ | √ | √ |
| *Sclerocroton cornutus* |  |  |  |  |  | √ |  |  |  |  |  |  |  |  |  |  |  |  |  |
| *Scutellaria baicalensis* |  |  |  |  |  |  |  | √ | √ |  |  |  |  |  |  |  | √ | √ | √ |
| *Seymeria pectinata* |  |  |  |  |  | √ |  |  |  |  |  |  |  |  |  |  |  |  |  |
| *Smilax herbacea* |  |  |  |  |  |  |  |  | √ |  |  |  |  |  |  |  |  |  |  |
| *Solanum* |  |  |  |  |  |  | √ | √ | √ |  |  |  |  |  |  |  | √ | √ | √ |
| *Stryphnodendron duckeanum* | |  |  |  |  |  | √ |  |  |  |  |  |  |  |  |  |  |  |  |
| *Suaeda maritima* |  |  |  | √ |  |  |  | √ |  |  |  |  |  |  |  |  |  | √ | √ |
| *Taraxacum laevigatum* | √ |  | √ |  |  | √ |  |  |  |  | √ | √ | √ | √ |  |  |  |  |  |

Note: The bold font of detected species means the prescribed herbal materials.

**Table S10. All detected species including PHS, SHS and CHS of the DHW preparations based on ITS2.**

| **Detected species** | **DHW.A** | | | | | | | | |  | **DHW.B** | | | | | | | | |
| --- | --- | --- | --- | --- | --- | --- | --- | --- | --- | --- | --- | --- | --- | --- | --- | --- | --- | --- | --- |
|  | **I1** | **I2** | **I3** | **II1** | **II2** | **II3** | **III1** | **III2** | **III3** |  | **I1** | **I2** | **I3** | **II1** | **II2** | **II3** | **III1** | **III2** | **III3** |
| ***Aconitum kusnezoffii*** |  |  |  | √ | √ |  |  |  | √ |  |  |  |  |  | √ | √ |  |  |  |
| ***Amomum compactum*** |  |  |  | √ | √ | √ | √ | √ | √ |  | √ | √ | √ | √ | √ | √ | √ | √ | √ |
| ***Anemone raddeana*** |  |  |  | √ | √ | √ | √ | √ | √ |  | √ | √ | √ | √ | √ | √ | √ | √ | √ |
| ***Angelica sinensis*** |  |  |  | √ | √ | √ | √ | √ | √ |  | √ | √ | √ | √ | √ | √ | √ | √ | √ |
| ***Aquilaria sinensis*** | **√** | **√** |  | √ | √ |  | √ |  | √ |  | √ | √ |  |  |  |  |  | √ |  |
| ***Asarum heterotropoides*** |  |  |  |  | √ | √ | √ | √ | √ |  |  |  |  |  |  |  |  | √ |  |
| ***Asarum sieboldii*** |  |  |  | √ | √ | √ | √ | √ | √ |  | √ | √ | √ | √ | √ | √ | √ | √ | √ |
| ***Atractylodes macrocephala*** |  |  |  | √ | √ | √ | √ | √ | √ |  |  | √ | √ | √ | √ |  | √ |  | √ |
| ***Clematis hexapetala*** | **√** | **√** | **√** | √ | √ | √ | √ | √ | √ |  | √ | √ | √ | √ | √ | √ | √ | √ | √ |
| ***Commiphora myrrha*** |  |  | **√** | √ | √ | √ | √ | √ | √ |  | √ | √ | √ |  | √ | √ | √ | √ | √ |
| ***Coptis chinensis*** |  |  |  | √ | √ | √ | √ | √ | √ |  | √ | √ | √ | √ | √ | √ | √ | √ | √ |
| ***Coptis deltoidea*** |  |  |  |  |  |  |  |  | √ |  |  |  |  |  |  |  |  |  |  |
| ***Coptis teeta*** | **√** | **√** | **√** | √ | √ | √ | √ | √ | √ |  | √ | √ | √ | √ | √ | √ | √ | √ | √ |
| ***Cyperus rotundus*** |  |  |  |  |  | √ | √ | √ | √ |  |  |  |  |  | √ |  |  |  |  |
| ***Ephedra equisetina*** |  |  |  | √ | √ | √ | √ | √ | √ |  | √ | √ | √ | √ | √ | √ | √ | √ | √ |
| ***Ephedra intermedia*** |  |  |  |  |  |  |  | √ |  |  |  |  |  |  |  |  |  |  |  |
| ***Ephedra sinica*** |  |  |  | √ | √ | √ | √ | √ | √ |  | √ | √ | √ | √ | √ | √ | √ | √ | √ |
| ***Gastrodia elata*** |  |  |  |  |  |  |  |  | √ |  |  |  |  |  |  |  |  |  |  |
| ***Glycyrrhiza glabra*** |  |  |  | √ | √ | √ | √ | √ | √ |  | √ | √ | √ |  | √ | √ | √ | √ | √ |
| ***Glycyrrhiza uralensis*** |  |  |  | √ | √ | √ | √ | √ | √ |  | √ | √ | √ | √ | √ | √ | √ | √ | √ |
| ***Lindera aggregata*** |  |  |  | √ | √ | √ | √ | √ | √ |  | √ | √ | √ | √ | √ | √ | √ | √ | √ |
| ***Notopterygium franchetii*** |  |  |  | √ | √ | √ | √ | √ | √ |  | √ | √ | √ | √ | √ | √ | √ | √ | √ |
| ***Notopterygium incisum*** |  |  |  | √ | √ | √ | √ | √ | √ |  | √ | √ | √ | √ | √ | √ | √ | √ | √ |
| ***Osmunda japonica*** | **√** |  |  | √ | √ | √ | √ | √ | √ |  | √ | √ | √ | √ | √ | √ | √ | √ | √ |
| ***Paeonia lactiflora*** | **√** | **√** | **√** | √ | √ | √ | √ | √ | √ |  | √ | √ | √ | √ | √ | √ | √ | √ | √ |
| ***Paeonia veitchii*** |  |  |  | √ | √ | √ | √ | √ | √ |  | √ | √ | √ | √ | √ | √ | √ | √ | √ |
| ***Panax ginseng*** |  |  |  | √ | √ | √ | √ | √ | √ |  | √ | √ | √ | √ | √ | √ | √ | √ | √ |
| ***Pogostemon cablin*** |  | **√** | **√** | √ | √ | √ | √ | √ | √ |  | √ | √ | √ | √ | √ | √ | √ | √ | √ |
| ***Rheum officinale*** |  |  |  | √ | √ | √ | √ | √ | √ |  | √ | √ | √ | √ | √ | √ | √ | √ | √ |
| ***Rheum palmatum*** |  | **√** |  | √ | √ | √ | √ | √ | √ |  | √ | √ | √ | √ | √ | √ | √ | √ | √ |
| ***Rheum tanguticum*** |  |  |  |  |  |  |  |  |  |  |  |  |  |  |  |  | √ |  |  |
| ***Saposhnikovia divaricata*** |  |  |  | √ | √ | √ | √ | √ | √ |  | √ | √ | √ |  | √ | √ | √ | √ | √ |
| ***Scrophularia ningpoensis*** |  |  |  |  | √ |  | √ |  |  |  | √ |  |  | √ |  |  | √ | √ |  |
| ***Scutellaria baicalensis*** |  |  |  | √ | √ | √ | √ | √ | √ |  |  | √ | √ |  | √ |  | √ | √ | √ |
| ***Styrax tonkinensis*** |  |  |  | √ | √ | √ | √ | √ | √ |  |  | √ |  | √ | √ | √ |  |  | √ |
| *Anemone nemorosa* |  |  |  | √ | √ | √ | √ | √ | √ |  | √ | √ | √ | √ | √ | √ | √ | √ | √ |
| *Anemone quinquefolia* |  |  |  | √ | √ | √ | √ | √ | √ |  |  |  |  |  |  |  | √ | √ | √ |
| *Coptis japonica* |  |  |  | √ | √ | √ | √ | √ | √ |  |  |  |  |  |  |  |  |  |  |
| *Lindera umbellata* |  |  |  | √ | √ | √ | √ | √ | √ |  |  |  |  |  |  |  |  | √ |  |
| *Paeonia suffruticosa* |  |  |  |  | √ | √ | √ | √ | √ |  |  |  | √ | √ |  |  | √ | √ |  |
| *Pogostemon heyneanus* |  |  |  | √ | √ | √ | √ | √ | √ |  | √ | √ | √ | √ | √ | √ | √ | √ | √ |
| *Pueraria montana* |  |  |  | √ | √ | √ | √ | √ | √ |  | √ | √ | √ |  | √ | √ | √ | √ | √ |
| *Cinnamomum aromaticum* |  |  |  | √ | √ | √ | √ | √ | √ |  |  | √ | √ | √ |  |  | √ | √ | √ |
| *Clematis vitalba* |  |  |  | √ |  |  |  | √ | √ |  |  |  |  |  | √ |  |  |  |  |
| *Amaranthus* |  | √ | √ | √ | √ | √ | √ | √ | √ |  | √ | √ | √ | √ | √ | √ | √ | √ | √ |
| *Glycine max* | √ | √ | √ | √ | √ | √ | √ | √ | √ |  | √ | √ | √ | √ | √ | √ | √ | √ | √ |
| *Senna tora* | √ | √ | √ | √ | √ | √ | √ | √ | √ |  | √ | √ | √ | √ | √ | √ | √ | √ | √ |
| *Cuscuta* | √ |  |  | √ | √ | √ | √ | √ | √ |  | √ | √ | √ | √ | √ | √ | √ | √ | √ |
| *Cussonia paniculata* |  |  |  | √ | √ | √ | √ | √ | √ |  | √ | √ | √ | √ | √ | √ | √ | √ | √ |
| *Hansenia* |  |  |  | √ | √ | √ | √ | √ | √ |  | √ | √ | √ | √ | √ | √ | √ | √ | √ |
| *Lycium barbarum* |  |  |  | √ | √ | √ | √ | √ | √ |  | √ | √ | √ | √ | √ | √ | √ | √ | √ |
| *Phyllanthus* |  |  |  | √ | √ | √ | √ | √ | √ |  | √ | √ | √ | √ | √ | √ | √ | √ | √ |
| *Zanthoxylum gilletii* |  |  |  | √ | √ | √ | √ | √ | √ |  | √ | √ | √ | √ | √ | √ | √ | √ | √ |
| *Entandrophragma angolense* |  |  |  |  | √ |  | √ | √ | √ |  | √ | √ | √ | √ | √ | √ | √ | √ | √ |
| *Nauclea officinalis* |  |  |  | √ | √ |  | √ |  | √ |  | √ | √ | √ | √ | √ | √ |  | √ | √ |
| *Vigna* | √ |  | √ | √ |  | √ | √ |  | √ |  |  |  | √ | √ | √ | √ | √ | √ |  |
| *Enterolobium timbouva* |  |  |  |  |  | √ | √ | √ | √ |  | √ | √ |  |  | √ | √ | √ | √ | √ |
| *Khaya grandifoliola* |  |  |  |  |  |  | √ |  | √ |  | √ | √ | √ | √ | √ | √ | √ | √ | √ |
| *Chenopodium* |  |  |  | √ | √ |  | √ | √ | √ |  |  |  | √ | √ |  | √ |  | √ | √ |
| *Carum buriaticum* |  |  |  | √ | √ | √ | √ | √ | √ |  |  | √ |  |  |  |  | √ | √ |  |
| *Vitellaria paradoxa* |  |  |  |  |  | √ |  |  | √ |  | √ | √ | √ |  | √ | √ | √ | √ |  |
| *Calycopteris floribunda* |  |  |  |  |  | √ |  | √ | √ |  |  |  | √ | √ | √ | √ |  |  | √ |
| *Nerium oleander* |  |  |  | √ |  |  |  |  |  |  | √ | √ | √ |  | √ | √ | √ |  | √ |
| *Ligusticum* | √ |  | √ | √ |  |  |  | √ |  |  |  |  |  |  |  | √ |  | √ | √ |
| *Oryza* | √ | √ | √ |  |  | √ |  |  |  |  |  |  |  |  |  | √ | √ | √ |  |
| *Rhododendron* |  |  |  | √ | √ | √ | √ | √ | √ |  |  |  |  |  |  |  |  |  | √ |
| *Ipomoea* |  |  |  |  |  |  | √ |  | √ |  |  |  | √ |  |  | √ | √ | √ |  |
| *Kali turgidum* |  |  |  |  | √ | √ | √ | √ | √ |  |  |  |  |  |  |  |  |  | √ |
| *Mitragyna speciosa* |  |  |  |  |  |  | √ |  |  |  |  |  | √ |  | √ | √ |  | √ | √ |
| *Populus nigra* |  |  |  | √ | √ | √ | √ | √ | √ |  |  |  |  |  |  |  |  |  |  |
| *Prunus* |  |  |  | √ | √ | √ | √ | √ | √ |  |  |  |  |  |  |  |  |  |  |
| *Bassia dasyphylla* |  |  |  | √ |  | √ | √ | √ | √ |  |  |  |  |  |  |  |  |  |  |
| *Cotinus coggygria* |  |  |  | √ | √ | √ | √ | √ |  |  |  |  |  |  |  |  |  |  |  |
| *Salix* |  |  |  | √ |  | √ | √ | √ | √ |  |  |  |  |  |  |  |  |  |  |
| *Saussurea costus* |  |  |  | √ | √ | √ | √ |  |  |  |  |  |  |  |  |  |  | √ |  |
| *Allium tuberosum* |  |  |  | √ |  |  |  |  |  |  |  |  |  |  | √ | √ | √ |  |  |
| *Brassica* | √ | √ | √ |  |  |  |  | √ |  |  |  |  |  |  |  |  |  |  |  |
| *Carissa carandas* |  |  |  |  |  |  |  |  |  |  |  |  |  |  | √ | √ | √ |  | √ |
| *Morus alba* |  |  |  |  |  |  | √ |  |  |  |  |  |  |  |  | √ | √ |  | √ |
| *Rosa spinosissima* |  |  |  | √ | √ |  |  | √ |  |  |  |  |  |  |  |  |  |  | √ |
| *Solanum* | √ | √ | √ |  |  |  |  |  |  |  |  |  |  |  |  |  |  |  | √ |
| *Arachis* | √ | √ | √ |  |  |  |  |  |  |  |  |  |  |  |  |  |  |  |  |
| *Artemisia* | √ | √ |  |  |  |  |  |  |  |  |  |  |  |  | √ |  |  |  |  |
| *Broussonetia* |  |  |  |  |  | √ |  |  |  |  |  |  |  | √ |  |  |  | √ |  |
| *Carthamus lanatus* |  |  |  |  |  |  |  |  |  |  |  |  |  |  |  |  | √ | √ | √ |
| *Cucumis* | √ | √ | √ |  |  |  |  |  |  |  |  |  |  |  |  |  |  |  |  |
| *Harpephyllum caffrum* |  |  |  |  |  |  |  |  |  |  | √ |  |  |  | √ | √ |  |  |  |
| *Hymenidium hookeri* |  |  |  |  | √ |  | √ | √ |  |  |  |  |  |  |  |  |  |  |  |
| *Pediastrum duplex* |  |  |  |  |  |  |  |  |  |  |  |  |  | √ |  | √ | √ |  |  |
| *Alstonia scholaris* |  |  |  |  |  |  |  |  |  |  |  |  |  |  | √ |  |  |  | √ |
| *Astragalus membranaceus* |  |  |  |  | √ |  |  |  |  |  |  |  |  |  |  |  |  | √ |  |
| *Campylotropis macrocarpa* |  |  |  |  |  |  | √ |  | √ |  |  |  |  |  |  |  |  |  |  |
| *Dillenia pentagyna* |  |  |  |  |  |  |  |  |  |  | √ |  |  |  |  |  |  |  | √ |
| *Entada phaseoloides* |  |  |  |  |  |  | √ |  | √ |  |  |  |  |  |  |  |  |  |  |
| *Foeniculum vulgare* |  |  |  |  |  |  |  |  |  |  |  |  |  |  |  |  |  | √ | √ |
| *Isodon rubescens* |  |  |  |  |  |  |  |  |  |  | √ |  | √ |  |  |  |  |  |  |
| *Oxybasis glauca* |  |  |  |  |  |  | √ | √ |  |  |  |  |  |  |  |  |  |  |  |
| *Robinia pseudoacacia* |  |  |  |  |  |  |  | √ | √ |  |  |  |  |  |  |  |  |  |  |
| *Rubus coreanus* |  |  |  |  |  |  |  |  | √ |  |  |  |  |  |  |  | √ |  |  |
| *Symphonia globulifera* |  |  |  |  |  |  |  |  |  |  | √ |  |  |  |  |  | √ |  |  |
| *Ulmus procera* |  |  |  |  | √ |  |  |  | √ |  |  |  |  |  |  |  |  |  |  |
| *Lumnitzera racemosa* |  |  |  |  |  |  |  |  |  |  | √ |  |  |  |  |  |  |  |  |
| *Linum bienne* |  |  |  |  |  |  |  |  |  |  |  |  |  |  |  | √ |  |  |  |
| *Acacia inaequiloba* |  |  |  |  |  |  |  |  |  |  |  | √ |  |  |  |  |  |  |  |
| *Autranella congolensis* |  |  |  |  |  |  |  |  |  |  |  |  |  |  |  | √ |  |  |  |
| *Carex radiata* |  |  |  |  |  |  |  |  |  |  |  |  |  |  |  |  | √ |  |  |
| *Gardenia jasminoides* |  |  |  |  |  |  |  |  |  |  |  |  |  |  |  |  |  |  | √ |
| *Pseudosenegalia riograndensis* |  |  |  |  |  |  |  |  |  |  |  |  |  |  |  |  | √ |  |  |
| *Corispermum ochotense* |  |  |  |  |  |  |  |  |  |  |  |  |  | √ |  |  |  |  |  |
| *Cheirodendron trigynum* |  |  |  |  |  |  |  |  |  |  |  |  |  | √ |  |  |  |  |  |
| *Eruca vesicaria* |  |  |  |  |  |  |  |  |  |  |  |  |  |  |  | √ |  |  |  |
| *Rauvolfia verticillata* |  |  |  |  |  |  |  |  |  |  |  | √ |  |  |  |  |  |  |  |
| *Justicia procumbens* |  |  |  |  |  |  |  |  |  |  |  |  |  | √ |  |  |  |  |  |
| *Cinchona pubescens* |  |  |  |  |  |  |  |  |  |  |  |  |  |  | √ |  |  |  |  |
| *Hordeum vulgare* |  |  |  |  |  |  |  |  |  |  |  | √ |  |  |  |  |  |  |  |

Note: The bold font of detected species means the prescribed herbal materials.

**Table S11. All detected species including PHS, SHS and CHS of the DHW preparations based on *trnL*.**

| **Detected species** | **DHW.A** | | | | | | | | |  | **DHW.B** | | | | | | | | |
| --- | --- | --- | --- | --- | --- | --- | --- | --- | --- | --- | --- | --- | --- | --- | --- | --- | --- | --- | --- |
|  | **I1** | **I2** | **I3** | **II1** | **II2** | **II3** | **III1** | **III2** | **III3** |  | **I1** | **I2** | **I3** | **II1** | **II2** | **II3** | **III1** | **III2** | **III3** |
| ***Anemone raddeana*** | √ | √ | √ | √ | √ | √ | √ | √ | √ |  | √ | √ | √ | √ | √ | √ | √ | √ | √ |
| ***Aquilaria sinensis*** | √ | √ | √ | √ | √ | √ | √ | √ | √ |  | √ | √ | √ | √ |  | √ | √ | √ | √ |
| ***Asarum sieboldii*** | √ | √ | √ | √ | √ | √ | √ | √ | √ |  |  |  |  | √ | √ |  |  | √ |  |
| ***Boswellia neglecta*** | √ | √ | √ | √ | √ | √ | √ | √ | √ |  | √ | √ | √ | √ | √ | √ | √ | √ | √ |
| ***Cinnamomum cassia*** | √ | √ | √ | √ | √ | √ | √ | √ | √ |  | √ | √ | √ | √ | √ | √ | √ | √ | √ |
| ***Clematis hexapetala*** | √ | √ | √ |  |  | √ |  | √ |  |  |  |  |  |  |  |  |  | √ |  |
| ***Coptis chinensis*** | √ | √ | √ | √ | √ | √ | √ | √ | √ |  | √ | √ | √ | √ | √ | √ | √ | √ | √ |
| ***Cyperus rotundus*** | √ | √ |  |  | √ |  |  | √ | √ |  |  |  |  |  |  |  |  |  |  |
| ***Ephedra equisetina*** |  |  |  |  |  | √ |  |  | √ |  |  |  |  |  |  |  |  |  |  |
| ***Ephedra sinica*** | √ | √ | √ | √ | √ | √ | √ | √ | √ |  |  |  | √ | √ | √ |  | √ | √ |  |
| ***Glycyrrhiza uralensis*** | √ | √ | √ | √ | √ | √ | √ | √ | √ |  | √ | √ | √ | √ | √ | √ | √ | √ | √ |
| ***Panax ginseng*** | √ | √ | √ | √ | √ | √ | √ | √ | √ |  | √ | √ | √ | √ | √ | √ | √ | √ | √ |
| ***Pogostemon cablin*** | √ | √ | √ | √ | √ | √ | √ | √ | √ |  | √ | √ | √ | √ | √ | √ | √ | √ | √ |
| ***Rehmannia glutinosa*** | √ | √ | √ | √ | √ | √ | √ | √ | √ |  | √ | √ | √ | √ | √ | √ | √ | √ | √ |
| ***Rheum officinale*** | √ |  | √ | √ | √ | √ | √ | √ | √ |  | √ |  | √ | √ | √ | √ | √ | √ | √ |
| ***Rheum tanguticum*** | √ |  | √ | √ | √ | √ | √ | √ | √ |  | √ |  | √ | √ | √ | √ | √ | √ | √ |
| ***Scrophularia ningpoensis*** |  | √ |  |  |  |  | √ |  |  |  |  |  |  |  |  |  |  |  |  |
| ***Scutellaria baicalensis*** | √ | √ | √ | √ | √ | √ | √ | √ | √ |  | √ | √ | √ | √ | √ | √ | √ | √ | √ |
| ***Angelica sinensis*** | √ | √ | √ | √ | √ | √ | √ | √ | √ |  | √ | √ | √ | √ | √ | √ | √ | √ | √ |
| ***Asarum heterotropoides*** | √ |  |  | √ | √ | √ | √ | √ | √ |  | √ | √ | √ | √ | √ | √ | √ | √ | √ |
| ***Atractylodes macrocephala*** | √ | √ | √ | √ | √ | √ | √ | √ | √ |  | √ | √ | √ | √ | √ | √ | √ | √ | √ |
| ***Coptis deltoidea*** | **√** | **√** | **√** | **√** | **√** | **√** | **√** | **√** | **√** |  | **√** | **√** | **√** | **√** | **√** | **√** | **√** | **√** | **√** |
| *Amomum* | √ | √ | √ | √ | √ | √ | √ | √ | √ |  |  |  |  |  |  | √ | √ | √ |  |
| *Anemone parviflora* | √ | √ | √ | √ | √ | √ | √ | √ | √ |  | √ | √ | √ | √ | √ | √ | √ | √ | √ |
| *Anemone ranunculoides* | √ | √ | √ |  |  |  |  |  |  |  |  |  |  |  |  |  |  |  |  |
| *Aquilaria rugosa* | √ | √ | √ |  |  |  |  |  |  |  |  |  |  |  |  |  |  |  |  |
| *Asarum europaeum* |  | √ | √ |  |  |  |  |  |  |  |  |  |  |  |  |  |  |  |  |
| *Asarum yakusimense* |  |  |  | √ | √ | √ | √ | √ | √ |  |  |  |  |  |  |  |  |  |  |
| *Clematis sibirica* | √ |  | √ |  |  |  |  |  |  |  |  |  |  |  |  |  |  |  |  |
| *Coptis japonica* | √ | √ | √ | √ | √ | √ | √ | √ | √ |  | √ | √ | √ | √ | √ | √ | √ | √ | √ |
| *Coptis quinquefolia* | √ | √ | √ | √ |  |  | √ |  | √ |  |  |  |  |  |  |  |  |  |  |
| *Paeonia suffruticosa* | √ | √ | √ | √ | √ | √ | √ | √ | √ |  | √ | √ | √ | √ | √ | √ | √ | √ | √ |
| *Pogostemon amaranthoides* | √ | √ | √ | √ | √ | √ | √ | √ | √ |  |  |  | √ |  |  | √ | √ | √ |  |
| *Pogostemon glaber* | √ | √ | √ | √ | √ | √ | √ | √ | √ |  | √ | √ | √ | √ | √ | √ | √ | √ | √ |
| *Polygonum orientale* |  |  |  | √ | √ | √ | √ | √ | √ |  | √ | √ | √ | √ | √ | √ | √ | √ | √ |
| *Scrophularia canina* | √ | √ | √ | √ | √ | √ | √ | √ | √ |  |  |  |  |  |  |  | √ | √ |  |
| *Styrax agrestis* | √ | √ |  |  |  |  |  |  |  |  |  |  |  |  |  |  |  |  |  |
| *Acaena laevigata* | √ | √ | √ |  |  |  |  |  |  |  |  |  |  |  |  |  |  |  |  |
| *Acalypha californica* |  |  |  |  | √ |  |  |  |  |  |  |  |  |  |  |  |  |  |  |
| *Achyrospermum carvalhoi* |  |  | √ | √ | √ |  | √ |  | √ |  |  |  |  |  |  |  |  |  |  |
| *Aconogonon alaskanum* | √ | √ |  |  |  |  |  |  |  |  |  |  |  |  |  |  |  |  |  |
| *Afrocanthium burttii* |  |  |  |  |  |  |  | √ |  |  | √ |  |  |  |  | √ |  | √ |  |
| *Allium schoenoprasum* |  |  |  |  |  |  |  |  |  |  |  | √ |  |  | √ |  |  |  |  |
| *Amaranthus sp.* |  |  |  |  |  |  |  |  |  |  |  |  |  |  | √ |  |  |  |  |
| *Amelanchier ovalis* | √ | √ | √ |  |  | √ |  |  |  |  |  |  |  |  |  |  |  |  |  |
| *Artemisia capillaris* | √ | √ | √ |  |  |  |  |  |  |  |  |  |  |  |  |  |  |  |  |
| *Astragalus alpinus* | √ |  | √ | √ | √ | √ | √ | √ | √ |  | √ | √ | √ | √ | √ | √ | √ | √ | √ |
| *Atriplex gmelinii* |  |  | √ | √ | √ | √ | √ | √ | √ |  | √ | √ | √ | √ | √ | √ | √ | √ | √ |
| *Bambusa bambos* |  |  | √ |  |  |  |  |  |  |  |  |  |  |  |  |  |  |  |  |
| *Baphiopsis parviflora* |  |  |  |  |  |  |  | √ |  |  |  |  |  |  |  |  |  | √ |  |
| *Bassia scoparia* |  |  |  |  |  | √ |  |  | √ |  |  |  |  |  |  |  |  |  |  |
| *Bauhinia glabra* |  |  | √ |  | √ |  |  | √ |  |  | √ | √ |  | √ | √ | √ | √ | √ | √ |
| *Bistorta vivipara* |  |  |  |  |  |  |  |  |  |  |  |  |  |  |  |  | √ |  |  |
| *Buddleja salviifolia* |  |  |  | √ | √ |  | √ |  | √ |  |  |  |  |  |  |  |  |  |  |
| *Caesalpinia* |  |  |  |  | √ |  |  | √ | √ |  | √ | √ | √ | √ | √ |  | √ | √ |  |
| *Chaiturus marrubiastrum* |  |  | √ | √ | √ | √ | √ | √ | √ |  | √ | √ | √ | √ | √ | √ | √ | √ | √ |
| *Chamaecrista* | √ |  | √ | √ | √ | √ | √ | √ | √ |  | √ | √ | √ | √ | √ | √ | √ | √ | √ |
| *Chenopodium* |  |  | √ | √ | √ | √ | √ | √ | √ |  | √ | √ | √ | √ | √ | √ | √ | √ | √ |
| *Chionochloa australis* |  |  |  |  |  |  |  | √ |  |  |  |  |  |  |  |  |  | √ |  |
| *Clymenia polyandra* | √ | √ |  |  |  |  |  |  |  |  | √ |  | √ |  |  | √ |  |  | √ |
| *Cocos nucifera* |  |  | √ |  |  |  |  |  |  |  |  |  |  |  |  |  |  |  |  |
| *Coeloneurum ferrugineum* |  |  | √ |  | √ |  | √ | √ | √ |  |  | √ | √ | √ | √ | √ | √ | √ | √ |
| *Cornus sanguinea* |  |  | √ | √ | √ | √ | √ | √ | √ |  | √ | √ | √ | √ | √ | √ | √ | √ | √ |
| *Corylus avellana* | √ | √ |  |  |  |  |  |  |  |  |  |  |  |  |  |  |  |  |  |
| *Craniolaria integrifolia* |  |  | √ | √ | √ | √ | √ | √ | √ |  |  |  |  |  | √ |  | √ |  |  |
| *Cuscuta* |  |  | √ | √ | √ | √ | √ | √ | √ |  | √ | √ | √ | √ | √ | √ | √ | √ | √ |
| *Cymbopogon citratus* |  |  | √ |  |  |  |  |  |  |  | √ |  |  |  |  | √ |  |  |  |
| *Cyphia crenata* |  |  | √ |  | √ |  |  | √ | √ |  |  |  |  |  |  | √ |  | √ |  |
| *Daucus carota* | √ | √ | √ | √ |  | √ | √ | √ | √ |  | √ | √ | √ | √ | √ | √ | √ | √ | √ |
| *Dendrocnide excelsa* | √ | √ | √ |  |  |  |  |  |  |  |  |  |  |  |  |  |  |  |  |
| *Dioscorea alata* |  | √ | √ | √ | √ | √ | √ | √ | √ |  | √ | √ | √ | √ | √ | √ | √ | √ | √ |
| *Echinochloa crus-galli* |  |  | √ |  |  | √ | √ | √ | √ |  | √ | √ | √ | √ | √ | √ | √ | √ | √ |
| *Entada* |  |  | √ | √ | √ | √ | √ | √ | √ |  | √ | √ | √ | √ | √ | √ | √ | √ | √ |
| *Gardenia vitiensis* |  |  |  | √ | √ | √ | √ | √ | √ |  | √ | √ | √ | √ | √ |  | √ | √ | √ |
| *Glycine max* |  | √ | √ | √ | √ | √ | √ | √ | √ |  | √ | √ | √ | √ | √ | √ | √ | √ | √ |
| *Gonostegia pentandra* |  | √ | √ |  |  |  |  |  |  |  |  |  |  |  |  |  |  |  |  |
| *Harveya speciosa* |  |  |  |  |  |  |  |  | √ |  |  |  |  |  |  |  |  |  |  |
| *Hedycarya arborea* | √ | √ | √ | √ | √ | √ | √ | √ |  |  | √ | √ | √ | √ | √ | √ | √ | √ | √ |
| *Hedychium coronarium* | √ | √ |  | √ |  |  |  |  |  |  |  |  |  |  |  |  |  |  |  |
| *Hoffmannseggia burchellii* |  |  | √ |  |  |  |  |  |  |  |  |  |  |  |  |  | √ |  |  |
| *Hordeum murinum* |  |  |  |  |  | √ |  |  |  |  | √ |  |  |  |  |  |  |  |  |
| *Ipomoea nil* |  |  |  |  | √ |  | √ | √ | √ |  | √ | √ | √ | √ | √ | √ | √ | √ |  |
| *Juniperus hybrid* |  | √ |  |  |  |  |  |  | √ |  |  |  |  |  |  |  |  |  |  |
| *Lactuca serriola* | √ | √ | √ | √ |  | √ | √ |  | √ |  |  |  |  |  |  |  |  |  |  |
| *Lamium galactophyllum* |  |  |  | √ |  |  | √ |  | √ |  |  | √ |  |  |  |  |  |  |  |
| *Latua pubiflora* |  | √ |  |  |  |  |  |  |  |  |  |  |  |  |  |  |  |  |  |
| *Lepechinia lancifolia* | √ |  | √ | √ |  | √ | √ |  |  |  |  |  |  |  |  |  |  |  |  |
| *Mandragora chinghaiensis* |  |  | √ | √ | √ | √ | √ | √ | √ |  | √ | √ | √ | √ | √ | √ | √ | √ | √ |
| *Moricandia moricandioides* |  | √ |  |  |  |  |  |  | √ |  |  |  |  |  |  |  |  |  |  |
| *Morus alba* |  |  |  |  | √ |  | √ | √ | √ |  |  |  |  | √ | √ | √ |  | √ | √ |
| *Musa acuminata* |  |  |  |  |  | √ |  | √ |  |  |  |  |  |  |  |  |  | √ |  |
| *Neolitsea sericea* | √ |  | √ | √ | √ | √ | √ | √ | √ |  | √ | √ | √ | √ | √ | √ | √ | √ | √ |
| *Nicotiana sylvestris* |  |  | √ | √ | √ | √ | √ | √ | √ |  |  | √ | √ | √ | √ | √ | √ | √ | √ |
| *Nolana linearifolia* |  |  | √ | √ | √ | √ | √ | √ | √ |  | √ | √ | √ | √ | √ | √ | √ | √ | √ |
| *Operculina sp.* |  |  | √ | √ | √ | √ | √ | √ | √ |  |  | √ | √ | √ | √ | √ | √ | √ | √ |
| *Ophiopogon japonicus* |  |  |  |  |  |  | √ |  |  |  |  |  |  | √ |  |  |  |  |  |
| *Oryza sativa* |  |  |  | √ |  |  |  |  |  |  | √ | √ | √ |  |  | √ | √ |  |  |
| *Pachypleurum alpinum* |  | √ | √ | √ |  |  | √ |  | √ |  |  |  |  |  |  |  |  |  |  |
| *Papaver rhoeas* |  |  |  |  |  |  |  |  |  |  |  |  |  |  | √ |  |  |  |  |
| *Pentaclethra macrophylla* |  |  |  |  |  |  |  |  | √ |  |  |  |  |  |  |  | √ |  |  |
| *Perilla frutescens* |  |  |  | √ | √ | √ | √ |  |  |  |  |  |  |  | √ |  |  |  |  |
| *Persea* | √ | √ | √ |  |  |  |  | √ |  |  |  |  |  |  |  |  |  | √ |  |
| *Persicaria maculosa* |  |  |  | √ |  |  | √ |  |  |  |  |  |  |  |  |  |  |  |  |
| *Petunia integrifolia* |  |  | √ |  | √ | √ | √ | √ | √ |  | √ | √ | √ | √ | √ | √ |  | √ | √ |
| *Physalis philadelphica* |  |  |  |  |  |  |  |  |  |  |  |  |  |  |  | √ |  |  |  |
| *Pinus sylvestris* | √ | √ |  |  |  | √ |  |  |  |  |  |  |  |  |  |  |  |  |  |
| *Pistacia chinensis* | √ | √ | √ |  |  | √ | √ |  | √ |  |  |  |  |  |  |  |  |  |  |
| *Platycladus orientalis* | √ | √ |  |  |  |  |  |  | √ |  |  |  |  |  |  |  |  |  |  |
| *Platycodon grandiflorus* | √ |  | √ |  | √ |  |  | √ | √ |  | √ | √ |  | √ |  | √ | √ | √ | √ |
| *Populus lasiocarpa* | √ | √ | √ |  |  |  | √ | √ | √ |  |  |  |  |  |  |  |  | √ |  |
| *Pulsatilla vulgaris* | √ | √ | √ |  |  |  |  |  | √ |  |  |  |  |  |  |  |  |  |  |
| *Rumex crispus* | √ |  | √ | √ | √ | √ | √ | √ |  |  | √ | √ | √ | √ | √ | √ | √ | √ | √ |
| *Salvia* |  |  | √ | √ | √ | √ | √ | √ | √ |  | √ | √ | √ | √ | √ | √ | √ | √ | √ |
| *Senna occidentalis* |  |  |  | √ |  |  |  |  | √ |  |  |  |  |  |  |  |  |  |  |
| *Seymeria pectinata* |  |  | √ |  | √ | √ | √ | √ | √ |  | √ | √ | √ | √ | √ | √ |  | √ | √ |
| *Smilax herbacea* | √ | √ | √ | √ | √ | √ | √ | √ | √ |  |  | √ |  | √ | √ | √ | √ | √ | √ |
| *Solanum* | √ | √ | √ | √ | √ | √ | √ | √ | √ |  | √ | √ | √ | √ | √ | √ | √ | √ | √ |
| *Spiraea densiflora* | √ | √ | √ |  |  |  |  |  |  |  |  |  |  |  |  |  |  |  |  |
| *Stryphnodendron* |  |  | √ |  |  |  | √ |  |  |  |  |  |  | √ |  | √ | √ |  | √ |
| *Suaeda maritima* |  |  | √ |  | √ |  |  | √ |  |  | √ | √ | √ | √ | √ |  | √ | √ | √ |
| *Swida controversa* |  | √ | √ | √ | √ | √ | √ | √ | √ |  |  |  | √ |  | √ | √ |  | √ | √ |
| *Vigna unguiculata* |  |  |  |  | √ | √ |  | √ |  |  | √ |  | √ | √ | √ |  |  | √ | √ |

Note: The bold font of detected species means the prescribed herbal materials.

**Table S12. All detected species including PHS, SHS and CHS of the NJW preparations based on ITS2.**

| **Detected species** | **NJW.A** | | | | | | | | |  | **NJW.B** | | | | | | | | |
| --- | --- | --- | --- | --- | --- | --- | --- | --- | --- | --- | --- | --- | --- | --- | --- | --- | --- | --- | --- |
|  | **I1** | **I2** | **I3** | **II1** | **II2** | **II3** | **III1** | **III2** | **III3** |  | **I1** | **I2** | **I3** | **II1** | **II2** | **II3** | **III1** | **III2** | **III3** |
| ***Astragalus membranaceus*** | √ | √ | √ | √ | √ | √ | √ | √ | √ |  | √ | √ | √ | √ | √ | √ | √ | √ | √ |
| ***Curcuma wenyujin*** |  |  |  |  |  |  |  | √ |  |  |  |  |  |  |  |  |  |  |  |
| ***Nardostachys jatamansi*** | √ |  |  |  | √ |  | √ | √ | √ |  |  |  |  |  |  | √ |  |  |  |
| ***Paeonia lactiflora*** | √ | √ | √ | √ | √ | √ | √ | √ | √ |  | √ | √ | √ | √ | √ | √ | √ | √ | √ |
| ***Scutellaria baicalensis*** | √ |  | √ |  | √ |  | √ |  | √ |  |  |  | √ |  |  | √ |  |  |  |
| ***Senna tora*** | √ | √ | √ | √ | √ | √ | √ | √ | √ |  | √ | √ | √ | √ | √ | √ | √ | √ | √ |
| ***Senna obtusifolia*** | √ | √ | √ | √ | √ | √ | √ | √ | √ |  | √ | √ | √ | √ | √ | √ | √ | √ | √ |
| ***Codonopsis pilosula*** | √ | √ | √ | √ | √ | √ |  | √ | √ |  |  | √ | √ | √ | √ | √ | √ | √ |  |
| ***Curcuma kwangsiensis*** |  |  |  | √ |  | √ |  |  |  |  |  |  |  |  |  |  |  |  |  |
| ***Curcuma longa*** |  |  |  | √ |  |  |  |  |  |  |  |  |  |  |  |  |  |  |  |
| ***Ligusticum chuanxiong*** | √ | √ | √ | √ | √ | √ | √ | √ | √ |  | √ | √ | √ | √ | √ | √ | √ | √ | √ |
| ***Mentha haplocalyx*** | √ |  | √ |  | √ | √ | √ |  | √ |  |  |  | √ | √ |  |  |  |  |  |
| *Codonopsis kawakamii* | √ | √ | √ | √ | √ | √ |  | √ | √ |  |  |  |  | √ |  | √ |  |  |  |
| *Senna* |  |  |  | √ | √ | √ | √ | √ | √ |  |  |  |  |  |  |  |  |  |  |
| *Paeonia* |  |  |  | √ | √ |  |  |  |  |  |  |  |  | √ |  |  |  |  |  |
| *Astragalus americanus* |  |  |  |  |  |  | √ |  | √ |  |  |  |  |  |  |  | √ |  |  |
| *Mentha arvensis* | √ | √ | √ | √ | √ | √ | √ | √ | √ |  | √ | √ | √ | √ |  | √ | √ |  | √ |
| *Mentha canadensis* | √ | √ | √ |  | √ | √ | √ | √ | √ |  |  | √ | √ |  |  | √ |  |  |  |
| *Ligusticum* | √ |  | √ |  |  |  |  |  |  |  |  |  | √ |  |  | √ |  |  |  |
| *Ipomoea* | √ | √ | √ | √ | √ | √ |  |  |  |  |  |  |  | √ | √ | √ | √ | √ | √ |
| *Amaranthus* | √ |  | √ | √ |  |  |  | √ |  |  | √ | √ | √ | √ |  | √ |  |  |  |
| *Acalypha australis* |  |  |  |  |  |  |  |  |  |  | √ | √ | √ | √ |  | √ |  |  |  |
| *Anemone* | √ |  |  | √ |  |  |  |  |  |  | √ |  | √ | √ |  |  |  |  |  |
| *Corchorus olitorius* |  |  |  |  |  |  |  |  |  |  |  |  | √ |  |  |  |  |  |  |
| *Descurainia sophia* |  |  |  | √ | √ |  | √ | √ | √ |  |  |  |  |  |  |  |  |  |  |
| *Hibiscus acetosella* |  |  |  | √ | √ |  | √ | √ | √ |  |  |  |  |  |  |  |  |  |  |
| *Angelica* | √ |  |  | √ |  |  |  |  |  |  |  |  |  | √ |  |  |  |  |  |
| *Argyreia sp.* |  |  |  | √ | √ | √ |  |  |  |  |  |  |  |  |  |  |  |  |  |
| *Cuscuta* |  |  |  | √ |  |  |  |  | √ |  |  |  |  | √ |  |  |  |  |  |
| *Sesbania* |  |  |  | √ | √ | √ |  |  |  |  |  |  |  |  |  |  |  |  |  |
| *Sida* |  |  |  | √ | √ | √ |  |  |  |  |  |  |  |  |  |  |  |  |  |
| *Asarum sieboldii* |  |  |  | √ |  |  |  |  |  |  |  |  |  | √ |  |  |  |  |  |
| *Carum buriaticum* |  |  |  | √ |  |  |  |  |  |  |  |  |  | √ |  |  |  |  |  |
| *Cussonia paniculata* |  |  |  | √ |  |  |  |  |  |  |  |  |  | √ |  |  |  |  |  |
| *Glycine max* |  |  |  | √ |  |  |  |  |  |  |  |  |  | √ |  |  |  |  |  |
| *Glycyrrhiza uralensis* |  |  |  | √ |  |  |  |  |  |  |  |  |  | √ |  |  |  |  |  |
| *Hansenia* |  |  |  | √ |  |  |  |  |  |  |  |  |  | √ |  |  |  |  |  |
| *Hyptis suaveolens* |  |  |  |  | √ | √ |  |  |  |  |  |  |  |  |  |  |  |  |  |
| *Lycium barbarum* |  |  |  | √ |  |  |  |  |  |  |  |  |  | √ |  |  |  |  |  |
| *Panax ginseng* |  |  |  | √ |  |  |  |  |  |  |  |  |  | √ |  |  |  |  |  |
| *Phyllanthus sp.* |  |  |  | √ |  |  |  |  |  |  |  |  |  | √ |  |  |  |  |  |
| *Pogostemon* |  |  |  | √ |  |  |  |  |  |  |  |  |  | √ |  |  |  |  |  |
| *Pueraria montana* |  |  |  | √ |  |  |  |  |  |  |  |  |  | √ |  |  |  |  |  |
| *Tephrosia persica* |  |  |  | √ | √ |  |  |  |  |  |  |  |  |  |  |  |  |  |  |
| *Zanthoxylum gilletii* |  |  |  | √ |  |  |  |  |  |  |  |  |  | √ |  |  |  |  |  |

Note: The bold font of detected species means the prescribed herbal materials.

**Table S13. All detected species including PHS, SHS and CHS of the NJW preparations based on *trnL*.**

| **Detected species** | **NJW.A** | | | | | | | | |  | **NJW.B** | | | | | | | |
| --- | --- | --- | --- | --- | --- | --- | --- | --- | --- | --- | --- | --- | --- | --- | --- | --- | --- | --- |
|  | **I1** | **I2** | **I3** | **II1** | **II2** | **II3** | **III1** | **III2** | **III3** |  | **I1** | **I2** | **I3** | **II1** | **II2** | **II3** | **III1** | **III3** |
| ***Codonopsis pilosula*** |  |  |  |  |  |  |  |  |  |  |  |  | √ |  |  |  |  |  |
| ***Curcuma kwangsiensis*** | √ |  |  | √ |  |  |  |  |  |  | √ |  |  |  |  |  | √ |  |
| ***Curcuma longa*** | √ |  |  | √ | √ |  |  |  |  |  | √ | √ | √ |  |  |  | √ |  |
| ***Curcuma phaeocaulis*** | √ |  |  | √ | √ |  |  | √ | √ |  | √ | √ | √ |  |  |  | √ |  |
| ***Nardostachys chinensis*** | √ | √ | √ | √ | √ | √ | √ | √ | √ |  | √ | √ | √ | √ | √ | √ | √ | √ |
| ***Nardostachys jatamansi*** |  |  |  |  |  |  |  | √ |  |  |  |  |  |  |  |  |  |  |
| ***Scutellaria baicalensis*** |  |  |  |  |  |  |  |  | √ |  | √ |  | √ | √ |  | √ |  | √ |
| *Astragalus* | √ |  |  | √ |  |  | √ |  | √ |  | √ |  | √ | √ |  | √ | √ | √ |
| *Mentha spicata* | √ | √ | √ | √ | √ | √ | √ | √ |  |  | √ | √ |  |  | √ |  | √ |  |
| *Scutellaria galericulata* | √ | √ |  | √ | √ | √ | √ | √ | √ |  | √ | √ |  |  |  |  |  |  |
| *Senna* | √ | √ | √ | √ | √ | √ | √ | √ | √ |  | √ | √ | √ |  | √ |  | √ |  |
| *Acalypha californica* | |  |  |  |  |  |  |  |  |  | √ | √ | √ | √ | √ |  |  | √ |
| *Andira humilis* | √ | √ | √ | √ | √ | √ | √ | √ | √ |  | √ | √ | √ | √ | √ | √ | √ | √ |
| *Anemone* | √ | √ | √ | √ | √ |  |  |  | √ |  | √ | √ | √ |  |  |  | √ |  |
| *Artocarpus altilis* | √ | √ | √ | √ | √ | √ | √ | √ |  |  | √ | √ |  | √ | √ |  | √ |  |
| *Atriplex gmelinii* | √ |  |  | √ |  |  |  |  |  |  | √ |  |  |  |  |  | √ |  |
| Bassia scoparia | |  | √ | √ |  | √ | √ | √ |  |  | √ | √ |  |  |  |  | √ |  |
| Biscutella laevigata | | √ | √ | √ | √ | √ | √ | √ |  |  | √ | √ |  |  | √ |  |  |  |
| *Bistorta* | √ | √ | √ | √ | √ | √ | √ | √ | √ |  | √ | √ |  | √ | √ |  | √ | √ |
| *Caesalpinia* |  |  |  |  |  |  |  |  | √ |  |  |  | √ | √ |  | √ |  | √ |
| *Caragana jubata* | √ |  |  | √ |  |  |  |  |  |  | √ |  |  |  |  |  | √ |  |
| *Carex media* | √ | √ | √ | √ | √ | √ | √ | √ |  |  | √ | √ |  |  | √ |  | √ |  |
| *Cedrus deodara* | √ | √ | √ | √ | √ | √ | √ | √ |  |  | √ | √ |  |  | √ |  | √ |  |
| *Chamaecrista mimosoides* | √ |  |  | √ |  |  |  |  | √ |  | √ |  | √ | √ |  | √ | √ | √ |
| *Clinopodium acinos* | √ | √ |  | √ |  |  |  |  |  |  | √ | √ |  |  |  |  | √ |  |
| *Coffea semsei* | √ | √ | √ | √ |  |  | √ | √ |  |  | √ | √ |  |  |  |  | √ |  |
| *Coix lacryma-jobi* | √ |  |  | √ |  |  |  |  |  |  | √ |  |  |  |  |  | √ |  |
| *Cryptocarya* | √ |  | √ | √ |  |  |  |  |  |  | √ |  |  |  |  |  | √ |  |
| *Cuscuta epilinum* | √ |  |  |  |  |  |  |  |  |  | √ |  |  |  |  |  | √ |  |
| *Daucus carota* | √ |  |  |  |  |  |  |  |  |  | √ |  |  | √ |  |  | √ |  |
| *Deschampsia cespitosa* | √ | √ | √ | √ | √ | √ | √ | √ |  |  | √ | √ |  |  | √ |  | √ |  |
| *Descurainia sophia* | √ |  |  |  |  |  |  |  | √ |  |  |  |  |  |  |  |  |  |
| *Diplotropis triloba* | √ | √ |  | √ |  |  |  |  |  |  | √ | √ |  |  |  |  | √ |  |
| *Duranta fletcheriana* | √ |  | √ | √ | √ | √ | √ | √ |  |  | √ | √ |  |  | √ |  | √ |  |
| *Dyssochroma viridiflora* | √ |  |  | √ |  |  |  |  |  |  | √ | √ |  |  | √ |  | √ |  |
| *Echinochloa crusgalli* | √ |  |  | √ | √ |  | √ | √ |  |  | √ | √ |  |  | √ |  | √ |  |
| Entada abyssinica | |  |  |  |  |  |  |  | √ |  |  |  | √ | √ |  | √ |  | √ |
| *Euscaphis japonica* | √ |  |  | √ |  |  |  |  |  |  | √ |  |  |  |  |  | √ |  |
| *Griselinia racemosa* | √ | √ | √ | √ | √ | √ | √ | √ |  |  | √ | √ |  |  | √ |  | √ |  |
| *Hedycarya arborea* | √ | √ | √ | √ |  |  | √ | √ |  |  | √ | √ |  |  | √ |  | √ |  |
| Hoffmannseggia | | √ | √ |  |  |  |  | √ | √ |  |  |  | √ | √ |  | √ |  | √ |
| *Ipomoea* | √ |  |  |  |  |  |  |  | √ |  | √ |  | √ | √ |  | √ |  | √ |
| *Lactuca serriola* | √ |  |  | √ |  |  |  |  | √ |  | √ |  |  |  |  |  | √ | √ |
| *Lobelia* | √ |  |  | √ | √ | √ | √ | √ |  |  | √ | √ |  |  | √ |  | √ |  |
| *Lonicera caerulea* | √ |  |  | √ |  |  |  |  |  |  | √ |  |  |  |  |  | √ |  |
| *Medicago ruthenica* | √ |  |  |  |  |  |  | √ |  |  |  |  |  |  |  |  | √ |  |
| *Microtoena patchoulii* | √ | √ | √ | √ | √ | √ | √ | √ |  |  | √ | √ |  |  | √ |  | √ |  |
| *Ophiopogon japonicus* | √ | √ | √ | √ | √ | √ | √ | √ |  |  | √ | √ | √ |  | √ |  | √ |  |
| *Ormosia paraensis* | √ |  |  | √ |  |  |  |  |  |  | √ |  |  |  |  |  | √ |  |
| *Oryza* | √ | √ | √ | √ | √ |  |  |  |  |  | √ | √ | √ |  |  |  | √ |  |
| *Paeonia suffruticosa* | √ | √ | √ | √ | √ | √ | √ | √ | √ |  | √ | √ | √ | √ | √ | √ | √ | √ |
| Panax ginseng | |  |  |  |  |  |  |  | √ |  | √ |  |  |  |  |  | √ |  |
| *Platycladus orientalis* | √ |  |  | √ |  |  | √ | √ |  |  | √ | √ |  |  |  |  | √ |  |
| Platycodon grandiflorus | |  |  |  |  |  |  |  | √ |  |  |  | √ | √ |  | √ |  | √ |
| *Pogostemon* | √ |  | √ | √ | √ | √ |  |  |  |  | √ | √ | √ |  |  |  | √ |  |
| *Polygonum* | √ | √ | √ | √ | √ |  | √ | √ |  |  | √ | √ |  |  | √ |  | √ |  |
| Potentilla anserina | |  |  | √ | √ | √ | √ | √ |  |  | √ | √ |  |  |  |  |  |  |
| *Prostanthera galbraithiae* | √ |  |  | √ |  |  | √ | √ |  |  | √ | √ |  |  |  |  | √ |  |
| *Pulsatilla vulgaris* | √ |  |  | √ |  |  |  |  |  |  | √ |  |  |  |  |  | √ |  |
| *Salvia yangii* | √ | √ | √ | √ | √ | √ | √ | √ |  |  | √ | √ |  |  | √ |  | √ |  |
| *Saxifraga nelsoniana* | √ |  |  |  |  |  |  |  |  |  | √ |  |  |  |  |  | √ |  |
| Solanum hasslerianum | |  |  |  |  | √ | √ | √ |  |  |  | √ |  |  |  |  |  |  |
| *Stryphnodendron* |  |  |  |  |  |  |  | √ | √ |  |  |  | √ | √ |  | √ |  | √ |

Note: The bold font of detected species means the prescribed herbal materials.

**Table S14. All detected species including PHS, SHS and CHS of the YGW preparations based on ITS2.**

| **Detected species** | **YGW.A** | | | | | | | | |  | **YGW.B** | | | | | | | |
| --- | --- | --- | --- | --- | --- | --- | --- | --- | --- | --- | --- | --- | --- | --- | --- | --- | --- | --- |
|  | **I1** | **I2** | **I3** | **II1** | **II2** | **II3** | **III1** | **III2** | **III3** |  | **I1** | **I2** | **I3** | **II1** | **II2** | **II3** | **III2** | **III3** |
| ***Angelica sinensis*** | √ | √ | √ | √ | √ | √ | √ | √ | √ |  | √ | √ | √ | √ | √ | √ | √ | √ |
| ***Cornus officinalis*** | √ | √ | √ | √ | √ | √ | √ | √ | √ |  |  |  | √ |  |  | √ | √ |  |
| ***Cuscuta australis*** | √ | √ | √ | √ | √ | √ | √ | √ | √ |  | √ |  |  |  |  | √ |  |  |
| ***Cuscuta chinensis*** | √ | √ | √ | √ | √ | √ | √ |  | √ |  |  |  |  |  |  | √ |  |  |
| ***Eucommia ulmoides*** |  |  |  |  |  |  |  |  |  |  |  |  |  |  |  |  | √ |  |
| ***Lycium barbarum*** | √ | √ | √ | √ | √ | √ | √ | √ | √ |  |  |  | √ | √ |  | √ | √ | √ |
| ***Rehmannia glutinosa*** | √ | √ | √ | √ | √ | √ | √ | √ | √ |  | √ | √ | √ | √ | √ | √ | √ | √ |
| *Cinnamomum aromaticum* |  |  |  |  |  |  |  |  |  |  | √ | √ | √ | √ | √ |  | √ | √ |
| *Cuscuta* | √ | √ | √ | √ | √ | √ | √ | √ | √ |  | √ | √ | √ | √ | √ | √ | √ | √ |
| *Amaranthus dubius* | √ | √ | √ | √ | √ | √ | √ | √ | √ |  | √ | √ | √ | √ | √ | √ | √ | √ |
| *Anemone nemorosa* |  |  |  |  |  |  |  |  |  |  |  |  |  |  |  | √ |  |  |
| *Arachis hypogaea* |  |  |  |  |  |  |  |  |  |  | √ | √ | √ | √ | √ |  | √ | √ |
| *Artemisia lavandulifolia* |  |  |  |  |  |  |  |  |  |  | √ | √ | √ | √ | √ |  | √ | √ |
| *Brassica* |  |  | √ | √ | √ | √ | √ |  |  |  | √ | √ | √ | √ | √ | √ | √ | √ |
| *Capsicum annuum* |  |  |  |  |  |  |  |  |  |  | √ | √ | √ | √ | √ |  | √ | √ |
| *Chenopodium album* | √ | √ | √ | √ | √ | √ | √ | √ | √ |  |  |  |  |  |  |  |  |  |
| *Cucumis* |  |  |  |  |  |  |  |  |  |  | √ | √ | √ | √ | √ |  | √ | √ |
| *Cussonia paniculata* | √ | √ | √ | √ | √ | √ | √ | √ | √ |  |  |  |  |  |  | √ |  |  |
| *Entandrophragma angolense* | √ | √ | √ | √ | √ | √ | √ | √ | √ |  |  |  |  |  |  | √ |  |  |
| *Enterolobium timbouva* | √ | √ | √ | √ | √ | √ | √ | √ | √ |  |  |  |  |  |  |  |  |  |
| *Foeniculum vulgare* |  |  |  |  |  |  |  |  |  |  | √ | √ | √ | √ | √ | √ | √ | √ |
| *Glycine max* | √ | √ | √ | √ | √ | √ | √ | √ | √ |  | √ | √ | √ | √ | √ | √ | √ | √ |
| *Khaya grandifoliola* | √ | √ | √ | √ | √ | √ | √ | √ | √ |  |  |  |  |  |  |  |  |  |
| *Raphanus sativus* |  |  |  |  |  |  |  |  |  |  | √ | √ | √ | √ | √ |  | √ | √ |
| *Senna tora* |  |  |  |  |  |  |  |  |  |  | √ | √ | √ | √ | √ | √ | √ | √ |
| *Solanum tuberosum* |  |  |  |  |  |  |  |  |  |  | √ | √ | √ | √ | √ |  | √ | √ |
| *Vigna unguiculata* | √ | √ | √ | √ | √ | √ | √ | √ | √ |  |  | √ | √ |  |  |  |  |  |
| *Zanthoxylum gilletii* | √ | √ | √ | √ | √ | √ | √ | √ | √ |  |  |  |  |  |  | √ |  |  |
| *Paeonia* |  |  |  |  |  |  |  |  |  |  |  |  |  |  |  | √ |  |  |
| *Pogostemon* |  |  |  |  |  |  |  |  |  |  |  |  |  |  |  | √ |  |  |

Note: The bold font of detected species means the prescribed herbal materials.

**Table S15. All detected species including PHS, SHS and CHS of the YGW preparations based on *trnL*.**

| **Detected species** | **YGW.A** | | | | | | | | |  | **YGW.B** | | | | | | | | |
| --- | --- | --- | --- | --- | --- | --- | --- | --- | --- | --- | --- | --- | --- | --- | --- | --- | --- | --- | --- |
|  | **I1** | **I2** | **I3** | **II1** | **II2** | **II3** | **III1** | **III2** | **III3** |  | **I1** | **I2** | **I3** | **II1** | **II2** | **II3** | **III1** | **III2** | **III3** |
| ***Cinnamomum cassia*** | √ | √ | √ | √ | √ | √ | √ | √ | √ |  | √ | √ |  |  | √ |  |  | √ |  |
| ***Cuscuta australis*** | √ | √ | √ | √ | √ | √ | √ | √ | √ |  |  | √ |  |  |  |  |  |  |  |
| ***Cuscuta chinensis*** | √ | √ | √ | √ | √ | √ | √ | √ | √ |  |  |  |  |  |  |  |  |  |  |
| ***Lycium barbarum*** | √ | √ | √ | √ | √ | √ | √ | √ | √ |  | √ | √ | √ | √ | √ | √ | √ | √ | √ |
| ***Rehmannia glutinosa*** | √ | √ | √ | √ | √ | √ | √ | √ | √ |  | √ | √ | √ | √ | √ | √ | √ | √ | √ |
| *Dioscorea alata* |  | √ |  | √ |  |  |  |  |  |  | √ |  | √ | √ |  | √ | √ | √ | √ |
| *Aegilops markgrafii* |  |  |  |  |  |  |  |  |  |  |  |  |  |  |  |  |  | √ |  |
| *Aeschynomene indica* |  |  |  |  |  |  |  |  |  |  | √ |  | √ | √ | √ | √ | √ | √ | √ |
| *Amomum petaloideum* | √ | √ |  | √ | √ | √ |  |  |  |  |  | √ |  |  |  |  |  |  |  |
| *Anisomeles indica* |  |  |  |  |  |  |  |  |  |  | √ |  | √ | √ |  | √ | √ | √ | √ |
| *Asteraceae environmental* |  |  |  |  |  |  |  |  |  |  | √ |  | √ | √ | √ | √ | √ | √ | √ |
| *Astragalus alpinus* | √ | √ |  | √ |  |  |  |  |  |  |  | √ |  |  |  |  |  |  |  |
| *Atriplex gmelinii* | √ | √ | √ | √ | √ | √ | √ | √ | √ |  |  | √ |  |  |  |  |  |  |  |
| *Brassica* |  |  |  |  |  |  |  |  |  |  | √ |  | √ | √ | √ | √ | √ | √ | √ |
| *Chaiturus marrubiastrum* |  |  |  |  |  |  |  |  |  |  | √ | √ | √ | √ | √ | √ | √ | √ | √ |
| *Chamaecrista sp.* | √ | √ | √ | √ | √ | √ | √ | √ | √ |  |  | √ |  |  |  |  |  |  |  |
| *Chenopodium* | √ | √ | √ | √ | √ | √ | √ | √ | √ |  |  | √ |  |  |  |  |  |  |  |
| *Cymopterus coulteri* |  |  |  |  |  |  |  |  |  |  | √ |  | √ | √ | √ | √ | √ | √ | √ |
| *Daucus carota* | √ | √ | √ | √ | √ | √ | √ | √ | √ |  | √ |  | √ | √ | √ | √ | √ | √ | √ |
| *Echinochloa crus-galli* | √ | √ |  | √ | √ |  |  |  |  |  |  | √ |  |  |  |  |  |  |  |
| *Entada abyssinica* | √ | √ | √ | √ | √ | √ | √ | √ | √ |  |  | √ |  |  |  |  |  |  |  |
| *Gardenia vitiensis* | √ | √ | √ |  |  |  |  |  |  |  |  |  |  |  |  |  |  |  |  |
| *Glycine max* | √ | √ | √ | √ | √ | √ | √ | √ | √ |  | √ | √ | √ | √ |  | √ | √ | √ | √ |
| *Glycyrrhiza uralensis* |  |  |  |  |  |  |  |  |  |  | √ | √ | √ | √ | √ | √ | √ | √ | √ |
| *Ipomoea nil* | √ |  |  | √ |  | √ |  |  |  |  | √ | √ | √ | √ | √ | √ | √ | √ | √ |
| *Lamium* |  |  |  |  |  |  |  |  |  |  |  |  | √ | √ | √ | √ | √ | √ | √ |
| *Leonurus sibiricus* |  |  |  |  |  |  |  |  |  |  | √ |  | √ | √ | √ | √ | √ | √ | √ |
| *Mandragora chinghaiensis* | √ | √ | √ | √ | √ | √ | √ | √ | √ |  |  | √ |  |  |  |  |  |  |  |
| *Microtoena patchoulii* |  |  |  |  |  |  |  |  |  |  |  |  | √ | √ | √ | √ | √ | √ | √ |
| *Neolitsea sericea* | √ | √ | √ | √ | √ | √ | √ | √ | √ |  |  | √ |  |  |  |  |  |  |  |
| *Nolana linearifolia* | √ | √ |  |  | √ | √ | √ | √ | √ |  |  |  |  |  |  |  |  |  |  |
| *Oryza sativa* |  |  | √ |  |  |  |  |  |  |  | √ |  | √ | √ | √ | √ | √ | √ | √ |
| *Paeonia suffruticosa* | √ |  | √ | √ | √ |  |  | √ |  |  | √ | √ | √ | √ | √ | √ | √ | √ | √ |
| *Panax ginseng* |  | √ |  |  |  |  |  |  |  |  |  | √ | √ | √ | √ | √ | √ | √ | √ |
| *Phragmites australis* |  |  |  |  |  |  |  |  |  |  |  |  | √ | √ | √ | √ | √ | √ | √ |
| *Platycodon grandiflorus* |  |  |  |  |  |  |  |  |  |  |  |  | √ | √ | √ | √ | √ | √ | √ |
| *Polygonum orientale* | √ | √ |  | √ |  | √ |  |  |  |  |  |  |  |  |  |  |  |  |  |
| *Raphanus sativus* |  |  |  |  |  |  |  |  |  |  |  |  | √ | √ | √ | √ | √ | √ | √ |
| *Salvia* |  |  |  |  |  |  |  |  |  |  |  |  | √ | √ | √ | √ | √ | √ | √ |
| *Scutellaria* | √ | √ | √ | √ | √ |  |  |  | √ |  |  |  | √ | √ | √ | √ | √ | √ | √ |
| *Solanum* | √ | √ | √ | √ | √ | √ | √ | √ | √ |  |  | √ | √ | √ | √ | √ | √ | √ | √ |
| *Suaeda maritima* | √ | √ | √ | √ | √ | √ |  |  |  |  |  | √ |  |  |  |  |  |  |  |

Note: The bold font of detected species means the prescribed herbal materials.

**Table S16. Comparative parameters including accuracy, F1 score and ROC based on the candidate biomarkers selected by the MEI model in ITS2-sequenced DHW.**

|  | Accuracy (%) | F1 score | ROC (%) |
| --- | --- | --- | --- |
| *Rheum palmatum* | 86.66±0.4 | 1.00 | 64.89 |
| *Coptis chinensis* | 85.75±3.52 | 0.67 | 53.33 |
| *Panax ginseng* | 93.33±0.15 | 1.00 | 68.44 |
| *Ephedra equisetina* | 73.31±0.43 | 1.00 | 59.11 |
| *Lindera aggregata* | 85.37±3.25 | NaN | 55.11 |
| *Clematis hexapetala* | 73.33±0.18 | 0.67 | 55.11 |
| *Rheum* | 33.16±1.25 | NaN | 72.00 |
| *Clematis* | 56.88±1.78 | NaN | 53.11 |

NAN means the preparations from DHW.A were miss-predicated as that of DHW.B, while the preparations from DHW.B were miss-predicated as that of DHW.A.

**Table S17. The information for TCM preparations used in this study.**

| **TCM preparation** | **Manufacture** | **SampleID** | **TCM preparation** | **Manufacture** | **SampleID** |
| --- | --- | --- | --- | --- | --- |
| BYW | BYW.A | BYW.A.I | NJW | NJW.A | NJW.A.I |
|  |  | BYW.A.II |  |  | NJW.A.II |
|  |  | BYW.A.III |  |  | NJW.A.III |
|  | BYW.B | BYW.B.I |  | NJW.B | NJW.B.I |
|  |  | BYW.B.II |  |  | NJW.B.II |
|  |  | BYW.B.III |  |  | NJW.B.III |
| DHW | DHW.A | DHW.A.I | YGW | YGW.A | YGW.A.I |
|  |  | DHW.A.II |  |  | YGW.A.II |
|  |  | DHW.A.III |  |  | YGW.A.III |
|  | DHW.B | DHW.B.I |  | YGW.B | YGW.B.I |
|  |  | DHW.B.II |  |  | YGW.B.II |
|  |  | DHW.B.III |  |  | YGW.B.III |

Note: “BYW.A.I” means Bazhen Yimu Wan produced by manufacturer A with batch I.

**Table S18. ITS2 and *trnL* gene primer sequences.**

| **Primer name** | **Primer sequence (5'→3')** | **Abbreviation** | **References** |
| --- | --- | --- | --- |
| *trnL* c (forward) | CGAAATCGGTAGACGCTACG | *trnL* | ^1^ |
| *trnL* h (reverse) | CCATTGAGTCTCTGCACCTATC |  |  |
| S2F (forward) | ATGCGATACTTGGTGTGAAT | ITS2 | ^2,3^ |
| ITS4 (reverse) | TCCTCCGCTTATTGATATGC |  |  |

**Table S19. The barcodes of samples used for Illumina MiSeq PE300 sequencing.** (a) Based on ITS2 biomarker; (b) Based on *trnL* biomarker.

**(a) Based on ITS2 biomarker**

| **TCM preparation** | **SampleID** | **Barcode** | **Sequencing library** | **TCM preparation** | **SampleID** | **Barcode** | **Sequencing library** |
| --- | --- | --- | --- | --- | --- | --- | --- |
| BYW | BYW.A.I1 | CGACTCA | Y1 | DHW | DHW.A.I1 | CACTGTC | Y2 |
|  | BYW.A.I2 | CGAGCTG | Y1 |  | DHW.A.I2 | CAGACTA | Y2 |
|  | BYW.A.I3 | CGAGTGC | Y1 |  | DHW.A.I3 | CAGCGAC | Y2 |
|  | BYW.A.II1 | CGATCAC | Y1 |  | DHW.A.II1 | CGACGTC | NJS |
|  | BYW.A.II2 | CGATGAG | Y1 |  | DHW.A.II2 | CATGACG | NJS |
|  | BYW.A.II3 | CGCACGC | Y1 |  | DHW.A.II3 | CATCGTG | NJS |
|  | BYW.A.III1 | CGATCAC | BYS1 |  | DHW.A.III1 | CAGACTA | NJS |
|  | BYW.A.III2 | CGAGTGC | BYS1 |  | DHW.A.III2 | CAGTCTG | NJS |
|  | BYW.A.III3 | CGAGCTG | BYS1 |  | DHW.A.III3 | CAGTAGC | NJS |
|  | BYW.B.I1 | CAGCTGA | Y1 |  | DHW.B.I1 | CGTAGAC | NJS |
|  | BYW.B.I2 | CAGTAGC | Y1 |  | DHW.B.I2 | CGCTAGA | NJS |
|  | BYW.B.I3 | CAGTCTG | Y1 |  | DHW.B.I3 | CGCAGCG | NJS |
|  | BYW.B.II1 | CACTGTC | Y1 |  | DHW.B.II1 | CGCACGC | NJS |
|  | BYW.B.II2 | CAGACTA | Y1 |  | DHW.B.II2 | CGATGAG | NJS |
|  | BYW.B.II3 | CAGCGAC | Y1 |  | DHW.B.II3 | CGATCAC | NJS |
|  | BYW.B.III1 | CGTAGAC | BYS1 |  | DHW.B.III1 | CGAGTGC | NJS |
|  | BYW.B.III2 | CGCTAGA | BYS1 |  | DHW.B.III2 | CACGTCG | NJS |
|  | BYW.B.III3 | CGCAGCG | BYS1 |  | DHW.B.III3 | CAGCGAC | NJS |
| NJW | NJW.A.I1 | CATCATC | D2 | YGW | YGW.A.I1 | CGACTCA | BYS1 |
|  | NJW.A.I2 | CATACGA | D2 |  | YGW.A.I2 | CGACGTC | BYS1 |
|  | NJW.A.I3 | CAGTGCA | D2 |  | YGW.A.I3 | CATGACG | BYS1 |
|  | NJW.A.II1 | CACTACA | NJS |  | YGW.A.II1 | CAGTGCA | BYS1 |
|  | NJW.A.II2 | CAGACTA | D2 |  | YGW.A.II2 | CAGTCTG | BYS1 |
|  | NJW.A.II3 | CATCGTG | D2 |  | YGW.A.II3 | CAGTAGC | BYS1 |
|  | NJW.A.III1 | CAGTCTG | D2 |  | YGW.A.III1 | CATCGTG | BYS1 |
|  | NJW.A.III2 | CAGTAGC | D2 |  | YGW.A.III2 | CATCATC | BYS1 |
|  | NJW.A.III3 | CAGCTGA | D2 |  | YGW.A.III3 | CATACGA | BYS1 |
|  | NJW.B.I1 | CGTAGAC | M3T1 |  | YGW.B.I1 | AGATGTA | Y1 |
|  | NJW.B.I2 | CGCTAGA | M3T1 |  | YGW.B.I2 | CACGTCG | Y1 |
|  | NJW.B.I3 | CGCAGCG | D2 |  | YGW.B.I3 | CACTACA | Y1 |
|  | NJW.B.II1 | CACTGTC | NJS |  | YGW.B.II1 | ACGTCTA | Y1 |
|  | NJW.B.II2 | CGAGTGC | M3T1 |  | YGW.B.II2 | ACTACTG | Y1 |
|  | NJW.B.II3 | CAGCGAC | D2 |  | YGW.B.II3 | CACTACA | YGS10 |
|  | NJW.B.III1 | CGCACGC | D2 |  | YGW.B.III1 | ACTGATA | Y1 |
|  | NJW.B.III2 | CGATGAG | M3T1 |  | YGW.B.III2 | AGACAGA | Y1 |
|  | NJW.B.III3 | CGATCAC | M3T1 |  | YGW.B.III3 | AGACTAC | Y1 |

Note that the 142 samples were allocated in 10 runs (marked as Sequencing library), and the samples in the same run could be separated by using the barcode. Each sequencing library represents a run, and the samples in the same sequencing library were in the same batch for sequencing.

**(b) Based on *trnL* biomarker**

| **TCM preparation** | **SampleID** | **Barcode** | **Sequencing library** | **TCM preparation** | **SampleID** | **Barcode** | **Sequencing library** |
| --- | --- | --- | --- | --- | --- | --- | --- |
| BYW | BYW.A.I1 | AGTGATG | YHT | DHW | DHW.A.I1 | ACAGACA | M3T1 |
|  | BYW.A.I2 | AGTGCTA | YHT |  | DHW.A.I2 | ACAGTCG | D2 |
|  | BYW.A.I3 | ATACGTA | YHT |  | DHW.A.I3 | AGTGATG | YGS10 |
|  | BYW.A.II1 | ATGCATG | BYT |  | DHW.A.II1 | ATCATAC | YGS10 |
|  | BYW.A.II2 | ATGACAG | YHT |  | DHW.A.II2 | ATAGTGA | YGS10 |
|  | BYW.A.II3 | ATGACAG | BYT |  | DHW.A.II3 | ATAGCAC | YGS10 |
|  | BYW.A.III1 | ATCTGAG | BYT |  | DHW.A.III1 | ATACTGC | YGS10 |
|  | BYW.A.III2 | ATCTATC | BYT |  | DHW.A.III2 | ATACGTA | YGS10 |
|  | BYW.A.III3 | ATCGCTG | BYT |  | DHW.A.III3 | AGTGCTA | YGS10 |
|  | BYW.B.I1 | ACGAGCG | YHT |  | DHW.B.I1 | CACGCAC | YGS10 |
|  | BYW.B.I2 | ACGATGC | YHT |  | DHW.B.I2 | ATGTAGA | YGS10 |
|  | BYW.B.I3 | ACGCATC | YHT |  | DHW.B.I3 | ATGCTCA | YGS10 |
|  | BYW.B.II1 | ACAGACA | YHT |  | DHW.B.II1 | ATGCATG | YGS10 |
|  | BYW.B.II2 | ACAGTCG | YHT |  | DHW.B.II2 | ATGACAG | YGS10 |
|  | BYW.B.II3 | ACATCGA | YHT |  | DHW.B.II3 | ATCTGAG | YGS10 |
|  | BYW.B.III1 | CACGCAC | BYT |  | DHW.B.III1 | AGTCACG | YGS10 |
|  | BYW.B.III2 | ATGTAGA | BYT |  | DHW.B.III2 | AGTATGA | YGS10 |
|  | BYW.B.III3 | ATGCTCA | BYT |  | DHW.B.III3 | ATCGAGC | YGS10 |
| NJW | NJW.A.I1 | AGCAGTC | YGS10 | YGW | YGW.A.I1 | ACAGACA | BYT |
|  | NJW.A.I2 | AGTATGA | M3T1 |  | YGW.A.I2 | ACAGTCG | BYT |
|  | NJW.A.I3 | AGTGATG | M3T1 |  | YGW.A.I3 | ACATCGA | BYT |
|  | NJW.A.II1 | AGCATAG | YGS10 |  | YGW.A.II1 | ACGAGCG | BYT |
|  | NJW.A.II2 | AGCATAG | M3T1 |  | YGW.A.II2 | ACGATGC | BYT |
|  | NJW.A.II3 | AGTACTC | M3T1 |  | YGW.A.II3 | ACGCATC | BYT |
|  | NJW.A.III1 | AGTGCTA | D2 |  | YGW.A.III1 | ACGCTAG | BYT |
|  | NJW.A.III2 | ATACGTA | M3T1 |  | YGW.A.III2 | AGCACGA | BYT |
|  | NJW.A.III3 | ATACTGC | M3T1 |  | YGW.A.III3 | AGCAGTC | BYT |
|  | NJW.B.I1 | AGTACTC | YGS10 |  | YGW.B.I1 | AGTGCTA | YHT |
|  | NJW.B.I2 | ACAGACA | D2 |  | YGW.B.I2 | AGCACGA | BYS1 |
|  | NJW.B.I3 | ACATCGA | M3T1 |  | YGW.B.I3 | ATACGTA | YHT |
|  | NJW.B.II1 | ACGCTAG | M3T1 |  | YGW.B.II1 | AGCATAG | YHT |
|  | NJW.B.II2 | AGCACGA | R8 |  | YGW.B.II2 | AGCTCGC | YHT |
|  | NJW.B.II3 | AGCAGTC | M3T1 |  | YGW.B.II3 | AGTACTC | YHT |
|  | NJW.B.III1 | AGCTCGC | YGS10 |  | YGW.B.III1 | AGTATGA | YHT |
|  | NJW.B.III2 | ACGCATC | failed to build the library |  | YGW.B.III2 | AGTCACG | YHT |
|  | NJW.B.III3 | ACGAGCG | M3T1 |  | YGW.B.III3 | AGTGATG | YHT |

Note that the 142 samples were allocated in 10 runs (marked as Sequencing library), and the samples in the same run could be separated by using the barcode. Each sequencing library represents a run, and the samples in the same sequencing library were in the same batch for sequencing.

**Table S20. The number of samples of each TCM preparation based on ITS2 and *trnL* biomarker.**

| **Biomarker** | **BYW** | **DHW** | **NJW** | **YGW** | **Total** |
| --- | --- | --- | --- | --- | --- |
| ITS2 | 18 | 18 | 18 | 17 | 71 |
| *trnL* | 18 | 18 | 17 | 18 | 71 |

Note that one *trnL*-marked BYW sample was failed to amplify because of severe PCR inhibition, and one ITS2-marked YGW sample was failed to build the libra

**Code 1 for rarefaction analysis:**

## Use DHW_ITS2 data as an example:

library('ggplot2')

library('vegan')

data1=read.csv("local_path/DHW_ITS_rc.csv",header=T,sep=",", row.names = 1) ## row = species, column = sampleID

data1=t(data1)

xlab="# of sequences"

ylab="# of species detected"

rarecurve(data1, step = 1, col = "black", cex = 0.5,xlab=xlab,ylab=ylab,scipen = 200)

### Supplementary Figure 1C

**Code 2 for principal component analysis:**

## Use DHW_ITS2 data as an example:

data<-read.csv("local_path/DHW_ITS_pca.csv", header= T, row.names=1, sep=",") ## row = sampeID, column = species

group<-read.csv("local_path/DHW_ITS_pca_group.csv", header= T, row.names=1, sep=",")

data=t(data)

pca =dudi.pca(data[,1:ncol(t(data))], scannf=F, nf=5)

str(pca$li)

PC1 = pca$li[ ,1]

PC2 = pca$li[ ,2]

a<- pca$li

plotdata <- data.frame(rownames(pca$li),PC1,PC2,group[,2])

colnames(plotdata) <-c("ID","PC1","PC2","group")

pc1 <-floor(pca$eig[1]*10000/sum(pca$eig))/100

pc2 <-floor(pca$eig[2]*10000/sum(pca$eig))/100

PCA_DHW_ITS2 <-ggplot(plotdata, aes(PC1, PC2))+stat_ellipse(aes(color=group),level=0.8)+ geom_point(aes(colour=group,shape=group), size=8)+

labs(title="trnL",size=10, x=paste("PC1(",pc1,"%)"),y=paste("PC2(",pc2,"%)"))+

geom_vline(aes(xintercept=0),linetype="dotted")+

geom_hline(aes(yintercept=0),linetype="dotted")+

theme(axis.text.x=element_text(size=18,colour="black"),

axis.text.y=element_text(size=18,colour="black"),

axis.title.x=element_text(size=20),

axis.title.y=element_text(size=20))

PCA_DHW_ITS2 ### Supplementary Figure 7C

**References**

1 Taberlet, P. *et al.* Power and limitations of the chloroplast trnL (UAA) intron for plant DNA barcoding. *Nucleic Acids Res* **35**, e14, doi:10.1093/nar/gkl938 (2007).

2 Shilin, C. *et al.* Validation of the ITS2 region as a novel DNA barcode for identifying medicinal plant species. *PLoS One* **5**, e8613, doi:10.1371/journal.pone.0008613 (2010).

3 Beeck, M. O. D. *et al.* Comparison and Validation of Some ITS Primer Pairs Useful for Fungal Metabarcoding Studies. *PLoS One* **9**, e97629, doi:10.1371/journal.pone.0097629 (2014).
